# Identifying loci under selection via explicit demographic models

**DOI:** 10.1111/1755-0998.13415

**Published:** 2021-06-03

**Authors:** Hirzi Luqman, Alex Widmer, Simone Fior, Daniel Wegmann

**Affiliations:** ^1^ Institute of Integrative Biology ETH Zurich Zürich Switzerland; ^2^ Department of Biology University of Fribourg Fribourg Switzerland; ^3^ Swiss Institute of Bioinformatics Fribourg Switzerland

**Keywords:** approximate Bayesian computation, demography, genetic trade‐offs, genome scan, local adaptation, selection

## Abstract

Adaptive genetic variation is a function of both selective and neutral forces. To accurately identify adaptive loci, it is thus critical to account for demographic history. Theory suggests that signatures of selection can be inferred using the coalescent, following the premise that genealogies of selected loci deviate from neutral expectations. Here, we build on this theory to develop an analytical framework to identify loci under selection via explicit demographic models (LSD). Under this framework, signatures of selection are inferred through deviations in demographic parameters, rather than through summary statistics directly, and demographic history is accounted for explicitly. Leveraging the property of demographic models to incorporate directionality, we show that LSD can provide information on the environment in which selection acts on a population. This can prove useful in elucidating the selective processes underlying local adaptation, by characterizing genetic trade‐offs and extending the concepts of antagonistic pleiotropy and conditional neutrality from ecological theory to practical application in genomic data. We implement LSD via approximate Bayesian computation and demonstrate, via simulations, that LSD (a) has high power to identify selected loci across a large range of demographic‐selection regimes, (b) outperforms commonly applied genome‐scan methods under complex demographies and (c) accurately infers the directionality of selection for identified candidates. Using the same simulations, we further characterize the behaviour of isolation‐with‐migration models conducive to the study of local adaptation under regimes of selection. Finally, we demonstrate an application of LSD by detecting loci and characterizing genetic trade‐offs underlying flower colour in *Antirrhinum majus*.

## 1 INTRODUCTION

Elucidating the genetic basis of adaptation and identifying genetic determinants of population and species divergence are key foci in evolutionary biology. In natural systems, genetic variation is shaped by the demographic history (driven by the neutral processes of mutation, migration and drift) together with natural selection on loci underlying adaptive traits. While all gene genealogies are constrained by the demographic history of the population, the genealogies of loci affected by selection are perturbed and may differ in key characteristics compared to those evolving under neutrality, though converging patterns can arise (Bierne et al., 2011; Edmonds et al., 2004; Excoffier, Foll, et al., 2009; Li et al., 2012; Slatkin & Excoffier, 2012). Disentangling the genomic signatures generated by these two processes (i.e., correctly identifying adaptive loci) remains a prevailing challenge in the field of population genetics (Biswas & Akey, 2006; Horscroft et al., 2019; Luikart et al., 2003).

A multitude of methods have been developed that identify loci under selection as those whose summary statistics deviate from the genome‐wide distribution. These “outlier” approaches can generally be grouped into three classes: those that (a) detect regions of elevated differentiation between populations (via e.g. *F*_ST_‐related statistics), (b) detect regions of perturbed site frequency spectrum (SFS) via diversity or diversity‐related estimators (e.g., *π*, Tajima's *D*) and (c) detect regions of extensive linkage disequilibrium (LD) via haplotype statistics (e.g., extended haplotype homozygosity [EHH], integrated haplotype score) (Beaumont & Nichols, 1996; Biswas & Akey, 2006; Luikart et al., 2003; Oleksyk et al., 2010; Sabeti et al., 2002; Vitti et al., 2013). While in empirical studies inference of selection is often achieved through corroboratory evidence from multiple measures, the choice of a particular class and hence summary statistic is motivated by the type of selection one aims to infer; with the first geared towards divergent selection between populations and the others towards footprints of selection within single populations.

Under the premise that adaptive genetic variation is a function of both selective and neutral forces, accounting for the demographic history of the study system is critical for the correct identification of selected loci (Excoffier, Hofer, et al., 2009; François et al., 2016; Hoban et al., 2016; Hofer et al., 2009). This is commonly achieved by contrasting locus‐specific statistics against an estimate of the expected distribution of these statistics under demography alone, with the power of such an approach being a function of both the summary statistics used and the accuracy with which the neutral distribution is inferred. In the context of identifying local adaptation, the canonical statistics employed is *F*_ST_, and the first methods to infer its neutral distribution used simulations under an island model calibrated by matching the observed heterozygosity at each locus (Beaumont & Nichols, 1996; Excoffier, Foll, et al., 2009). Under island models, the distribution of sample allele frequencies is also well captured by Pólya distributions (Balding & Nichols, 1994; Rannala & Hartigan, 1996), which can be learned using likelihood‐based methods that jointly classify loci into neutral and selected classes (Beaumont & Balding, 2004; Foll & Gaggiotti, 2008; Galimberti et al., 2020). While generally powerful, these methods suffer from high false‐positive rates in the case of asymmetric divergence between populations (Galimberti et al., 2020; Lotterhos & Whitlock, 2014; Luu et al., 2017), which violates a key assumption of island models. This can be alleviated by using hierarchical island models (Foll et al., 2014; Galimberti et al., 2020) or a more flexible distribution to capture neutral allele frequencies (e.g., principal components analysis [PCA], Luu et al., 2017). For some natural systems, however, these approaches may still be insufficient to capture the demographic history of the population and a (potentially complex) demographic model should be used. Williamson et al. (2005), for instance, inferred such a model from putatively neutral loci and then identified loci under selection as those for which an additional selection parameter is required.

Rather than modelling selection explicitly, loci under selection may also be identified under pure demographic models through locus‐specific demographic parameters, under the premise that the demographic parameters of selected loci are expected to deviate from neutral expectations (Barton & Bengtsson, 1986; Charlesworth, 2009; Charlesworth et al., 1997; Fusco & Uyenoyama, 2011; Galtier et al., 2000; Gossmann et al., 2011; Petry, 1983; Sousa et al., 2013). Under coalescent theory, demographic models are parametrized by the effective sizes (*N*_E_) of each population and the effective rates of migration (*M*_E_) between them, which respectively describe the level of drift and gene flow within and between populations (Charlesworth, 2009; Petry, 1983). Importantly, both *N*_E_ and *M*_E_ may change through time. Different modes of selection and adaptive processes can be expected to alter these demographic parameters in different ways. In a single population, a selective sweep is expected to reduce *N*_E_ at selected and linked sites while diversifying selection is expected to increase it (Galtier et al., 2000; Gossmann et al., 2011). In the case of two or more populations connected by gene flow, balancing selection and adaptive introgression are expected to increase *M*_E_ at selected and linked sites, while divergent selection is expected to reduce *M*_E_ at those sites (Charlesworth et al., 1997; Geraldes et al., 2006; Petry, 1983; Won et al., 2005).

Notably, locus‐specific demographic parameters are not just informative about the strength (i.e., the magnitude of variation in *N*_E_ or *M*_E_) and mode of selection (i.e., a reduction or elevation of *N*_E_ or *M*_E_), but may also identify the population (or environment) in which selection acts (e.g., a reduction in *M*_E_ in one but not the other direction). Directional selection modulates fitness in natural populations by purging maladaptive alleles via extrinsic barriers such as hybrid or immigrant inviability, or lower fecundity (Naisbit et al., 2001; Nosil et al., 2005; Rundle & Whitlock, 2001; Schluter, 2000). This effectively reduces *M*_E_ at selected loci proportionally to the strength of selection (Petry, 1983). Under local adaptation, alternate alleles may confer higher fitness in their respective local environment but reduced fitness in the foreign environment (i.e., antagonistic pleiotropy [AP]), or an allele may confer higher fitness in its local environment but have no differential effect relative to the alternate allele in the foreign environment (i.e., conditional neutrality [CN]) (Anderson et al., 2013; Kawecki & Ebert, 2004; Savolainen et al., 2013). To date, such genetic trade‐offs have only been characterized in a handful of cases, primarily through demanding experiments involving the transplanting of alternate genotypes in their reciprocal environments (Anderson et al., ,^2013^, ^2014^; Oakley et al., 2014; Troth et al., 2018). Characterizing the nature of such trade‐offs directly from genomic data presents a promising complementary approach, applicable to natural populations, to investigate the genetic basis of local adaptation, a key concept in ecological genetics.

Inferring demographic parameters using coalescent theory is, however, computationally challenging, as the underlying but unknown genealogies must be integrated out numerically (Hey & Nielsen, 2007). As a result, there exists only a single likelihood implementation to infer locus‐specific and global demographic parameters jointly: an Markov chain Monte Carlo (MCMC) sampler that attributes loci to different classes (e.g., selected and neutral) and jointly infers the demographic parameters of a two‐population isolation‐with‐migration (IM) model for each group (Sousa et al., 2013). To extend this approach to more complex models, simulation‐based techniques such as approximate Bayesian computation (ABC) (Beaumont et al., 2002; Marjoram & Tavaré, 2006; Sisson et al., 2018) may be employed. The use of ABC to infer genome‐wide demographic parameters has a long tradition (Beaumont et al., 2002; Dussex et al., 2014; Sisson et al., 2018; Tavaré et al., 1997; Wegmann & Excoffier, 2010) and it may also be used to infer locus‐specific parameters in a hierarchical setting, but it is computationally challenging. Indeed, the dimensionality of a genome‐wide model is prohibitively large for any naïve Monte‐Carlo scheme as the probability that a simulation matches the data at all loci is virtually zero. When inferring genome‐wide parameters, loci are exchangeable and this problem is easily overcome by using summary statistics that are functions of all loci such as the (scaled) moments of the distribution of locus‐specific statistics (Tavaré et al., 1997; Wegmann et al., 2009). To benefit from this in a hierarchical setting, Bazin et al. (2010) proposed a two‐step algorithm in which hierarchical parameters are inferred based on moments of locus‐specific summary statistics, and locus‐specific parameters are then inferred with simulations of a single locus conducted with parameters drawn from the posterior distribution of the hierarchical parameters. As recently shown (Kousathanas et al., 2016), this approach can be generalized to arbitrary parameter dependencies when using an ABC‐MCMC setting that also eliminates the need for a two‐step approach. These approaches were successfully used to infer locus‐specific (Bazin et al., 2010) and cluster‐specific (Aeschbacher et al., 2013) migration rates, as well as locus‐specific selection coefficients (Foll, Poh, et al., 2014; Kousathanas et al., 2016) for up to several hundred loci. Scaling such inference up to whole genomes, however, remains difficult due to the requirement to simulate all loci.

In this paper, we introduce LSD, a framework for identifying loci under selection via explicit demographic models that scales to genomic data. Similar to the approach by Bazin et al. (2010), LSD works in two steps. However, rather than inferring hierarchical parameters for all loci, LSD first obtains point estimates of demographic parameters for neutral loci, which is then compared against per‐locus estimates to identify selected loci. This has the benefit of requiring only simulations of a single locus, which can be efficiently recycled (Thalmann et al., 2011). As a downside, the approach requires a priori knowledge on putative neutral sites. However, as we show with simulations, the approach is very robust to mis‐specifications.

While LSD is flexible regarding the choice of demographic model and can in principle accommodate any discrete population model (including single population and stepping‐stone models) as well as detect different modes of selection, we demonstrate here LSD’s utility in studies of local adaptation by focusing on the detection of loci under divergent selection between populations under IM models. We validate and assess the performance of LSD via extensive simulations, provide general insights into the properties of IM models in relation to the power of LSD and other widely applied genome scan methods, and demonstrate an application of the method to the detection of functionally validated loci underlying flower colour in two parapatric subspecies of *Antirrhinum majus* (common snapdragon) (Schwinn et al., 2006; Tavares et al., 2018).

## 2 MATERIALS AND METHODS

### 2.1 Model

We begin by outlining the conceptual framework underlying LSD. Consider a demographic model M, parameterized by demographic parameters θ, that generates genetic data D. To quantify deviations from neutrality, LSD first estimates the demographic parameters $\hat{\theta }$ from a collection of loci assumed to be neutral (Figure S1). In a second step, LSD performs demographic inference on all loci and determines the posterior distribution $\pi_{l}\left(\theta \right)=\pi \left(\theta |D_{l}\right)$ for each locus. Finally, LSD assesses the concordance of $\hat{\theta }$ with $\pi_{l}\left(\theta \right)$ by determining $h_{l}$, the highest posterior density interval (HPDI) of $\pi_{l}\left(\theta \right)$ that contains $\hat{\theta }$, and uses $p_{l}=1-h_{l}$ as a metric to identify locus l as incompatible with $\hat{\theta }$. For loci with parameters $\hat{\theta }$, $p_{l}$ follows a uniform distribution (the coverage property) and is interpreted as a *p*‐value to reject $\hat{\theta }$ for locus l. The joint posterior distribution $\pi_{l}\left(\theta \right)$ may further provide information on the magnitude and directionality of selection.

**FIGURE 1:**
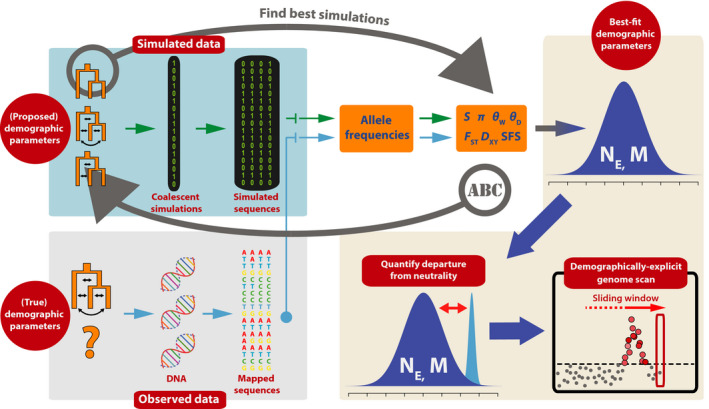
Analytical framework to identify loci under selection via explicit demographic models (LSD). LSD identifies loci under selection by first estimating demographic parameters and then quantifying the departure of these parameters from neutral expectations. Our specific implementation of LSD employs approximate Bayesian computation (ABC) for parameter estimation, and is performed in a genome scan approach

Given that the evaluation of the likelihood is nontrivial and may be intractable under more complex models, we resort to an approximate approach (Marjoram & Tavaré, 2006) (Figure 1). Under an ABC framework, the likelihood is approximated by simulations, the outcomes of which are compared with observed data in terms of summary statistics. That is, we find the set of parameters θ that minimize the distance between the observed data D and the simulated data $D^{\prime }$. To efficiently evaluate this, we reduce the dimensionality of the data via summarizing them into a set of lower‐dimensional summary statistics S and $S^{\prime }$, which are selected to capture the relevant information in D and $D^{\prime }$, respectively (Beaumont et al., 2002; Joyce & Marjoram, 2008; Sisson et al., 2018).

An appropriate model for generating simulated genetic data is provided by coalescent theory (Kingman, 1982; Wakeley, 2001), parametrized by population demographic parameters $\theta =\{N_{E},M_{E},µ\}$, where $N_{E}$ refers to the vector of effective population sizes, $M_{E}$ to the vector of effective migration rates and µ to the mutation rate. We stress that population sizes and migration rates may vary through time.

### 2.2 Implementation

We implemented the framework described above as shown in Figure 1 and detailed below.

#### 2.2.1 Simulations

While the framework is readily used for any type of locus, we consider here a locus to consist of a genomic window with a shared genealogy. This effectively implies that recombination is free between loci and absent within. We simulate genealogies using msms (Ewing & Hermisson, 2010), under a user‐defined demographic model. The processing, format and final output of observed genetic data will often differ from that of coalescent simulations, given that observed genetic data may be subject to various presequencing (e.g., pooling), sequencing (e.g., sequencing errors, stochastic sampling of reads) and postsequencing (e.g., filters) events that perturb and reformat the data from the original source. We thus implement two complementary programs that interface with coalescent simulators to replicate observed sequencing pipelines and generate simulated sequencing data: lsd‐high can accommodate and simulate both individual and pooled data and assumes mid‐ to high coverage (>10×) data, while lsd‐low accepts individual data and can additionally accommodate low coverage (>2×) data by utilizing genotype likelihoods via mstoglf and angsd (Korneliussen et al., 2014). A suite of summary statistics is then calculated for the simulated and observed data via the same programs. Summary statistics currently implemented include the number of segregating sites (*S*), private *S*, nucleotide diversity (*π*), Watterson's estimator (*θ*_W_), Tajima's *D* (*θ*_D_), relative divergence (*F*_ST_), absolute divergence (*D*_XY_) and site frequencies. In principle any summary statistic can be included, contingent on the data and appropriate additions to the programs’ scripts. To account for potential correlation between summary statistics and to retain only their informative components, we apply a partial least squares transformation (Wegmann et al., 2009).

#### 2.2.2 ABC inference

The estimation of demographic parameters is performed with abctoolbox (Wegmann et al., 2010), via the ABC‐GLM algorithm using the subset of *n* simulations closest to the observed summary statistics. In a first step, LSD infers demographic parameters of putatively neutral loci to obtain point estimates $\hat{\theta }$. We do so based on simulations of a single locus. In contrast to classic ABC regression approaches, ABC‐GLM can readily use such simulations to accurately infer posterior distributions from many loci as it approximates the likelihood function, rather than the posterior distribution (Thalmann et al., 2011). However, we propose a slightly different approach that does not characterize the full posterior distribution, but we found to result in point estimates $\hat{\theta }$ that are more accurate (Text S4, Figures S1 and S2) and robust to misidentification of putatively neutral loci (Text S5, Figure S3). Specifically, we first infer locus‐specific posterior distributions $\pi_{l}\left(\theta \right)$ for each putatively neutral locus with ABC‐GLM, then calculate the product of these densities $\pi \left(\theta \right)=\prod_{l}\pi_{l}\left(\theta \right)$, and identify $\hat{\theta }=\underset{\theta }{\mathrm{argmax}}\pi (\theta )$.

In a second step, LSD infers locus‐specific posterior distributions using ABC‐GLM on all loci, either using the same set of simulations as in the first step, or from simulations of a single locus conducted under the parameters $\hat{\theta }$, except for the parameters affected by selection (e.g., *M*_E_).

### 2.3 Simulations

To test the performance of the LSD implementation, we simulated pseudo‐observed genomes using the program msms under different demographic and selection parameter values, focusing on IM models relevant for the characterization of local adaptation. We assumed a diploid system, a common mutation rate *µ* = 5 × 10^−7^ per bp per generation, and all loci to be biallelic with ancestral allele *a* and derived allele *A*. Each simulated pseudogenome represented a unique demographic‐selection regime and comprised $n_{n}$ = 1000 neutral loci and $n_{s}$ = 50 selected loci of 5 kb length, for a total (pseudogenome) size of 5.25 Mb. We assume no within‐locus recombination.

#### 2.3.1 Demography

**FIGURE 2:**
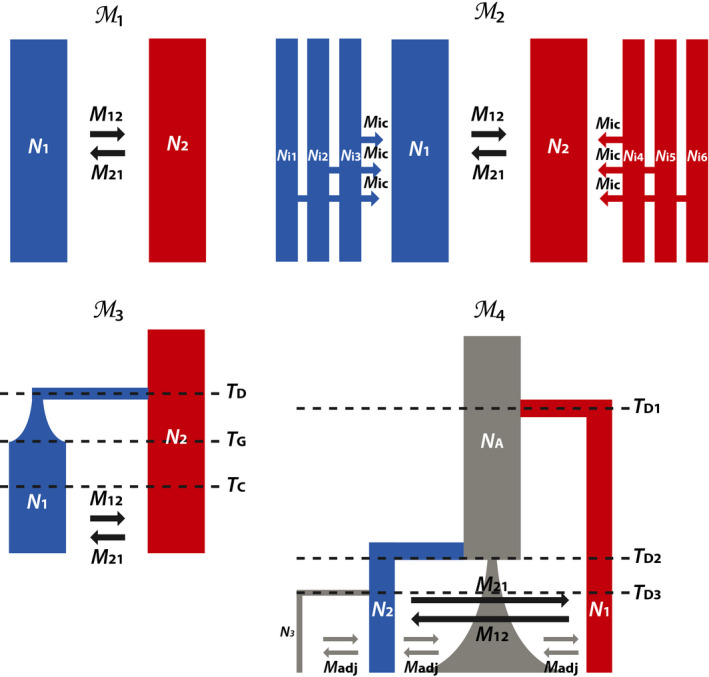
Models used in the simulations and case study. Model $M_{1}$ represents a simple two‐deme isolation‐with‐migration (IM) model with reciprocal migration. Model $M_{2}$ represents a six‐deme island–continent model where common differences between environments are modelled by connecting the sampled demes (i.e., islands) to respective meta‐population continents via gene flow. Model $M_{3}$ represents a two‐deme divergence with bottleneck and exponential growth model. Model $M_{4}$ represents a four‐deme hierarchical divergence model with sequential founder events, bottleneck and exponential growth. Red and blue demes reflect contrasting environments, while grey demes reflect neutral environments where no selection acts. In all models, selection is inferred from the deviation from neutrality of the reciprocal migration rates between the two contrasting environments ($M_{12}$, $M_{21}$)

We simulated four models representing different levels of complexity in terms of population structure and demographic history (Figure 2; Text S1). In all models, selection is inferred from the reciprocal scaled migration rates between two contrasting environments ($M_{12}$, $M_{21}$; where $M_{E}=Nm_{E}$). We used neutral migration rates $M_{12}$ = $M_{21}$ = M = 0.5, 5 and 50 migrants per generation and inferred selection as deviations from these rates. We use model $M_{1}$ to represent a simplified, generalized model of local adaptation, model $M_{2}$ to represent a more complex case of local adaptation comprising multiple, structured populations, model $M_{3}$ to reflect a scenario typical of glacial‐induced divergence and secondary contact population dynamics and model $M_{4}$ to represent a case of hierarchical divergence with complex demography. The specific parameter choices for all models are given in Text S1.

#### 2.3.2 Selection

To simulate genetic trade‐offs, selection was simulated on alternate alleles in the contrasting environments, on top of the demographic model. Specifically, we assumed the beneficial alleles to be dominant such that the relative fitness was 1 + *s*_1_, 1, 1 and 1, 1, 1 + *s*_2_ for the three genotypes *AA*, *Aa* and *aa* in the demes or metapopulations occupying the two environments, respectively. For the selection coefficients $s_{1}$ and $s_{2}$, we used all combinations of 0, 0.001, 0.01 and 0.1 and thus included cases of CN, in which either $s_{1}>0$, $s_{2}=0$ or $s_{1}=0$, $s_{2}>0$, as well as cases of AP with $s_{1}>0$, $s_{2}>0$. CN regimes are by definition always asymmetric, while AP regimes can be either symmetric ($s_{1}=s_{2}$) or asymmetric ($s_{1}\neq s_{2}$). We further varied the time of the onset of selection from $T_{S}$ = 400, 4000, 40,000 and 400,000 generations ago.

For all models, we considered selection on standing variation with the initial frequency of the derived allele at $f_{1}=f_{2}=0.1$ in all demes. For model $M_{1}$, we additionally investigated the case of de novo mutations with initial frequencies $f_{1}=\frac{1}{2N_{1}}$ and $f_{2}=0$. These two cases represent the often‐considered starting points for local adaptation (Peter et al., 2012). Depending on the selection regime and due to the stochasticity of drift, the derived allele *A* may sometimes be lost and hence be absent in the simulation of selected loci (especially in the de novo case). Because such a scenario contains no signal for detection of selection, we excluded such simulations (via the ‐SFC parameter in msms).

#### 2.3.3 Assessing accuracy

**FIGURE 3:**
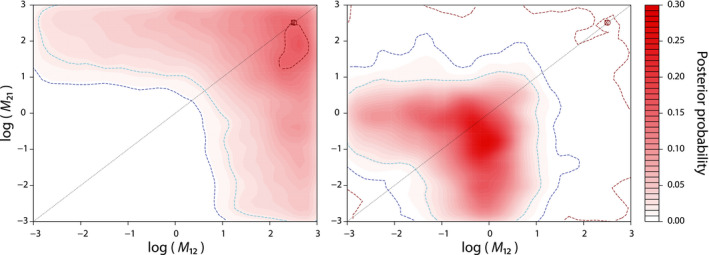
Exemplary joint posterior distribution of reciprocal migration parameters, $M_{12}$ and $M_{21}$. The neutral joint parameter estimate, as informed by the global posterior distribution of all neutral regions (Figure S1), is indicated by the red dot in the top right corner. The red contours represent the joint posterior distribution of a genomic region (i.e., window), with the blue contours representing the 95% (light blue) and 99% (dark blue) highest density region (HDR) credible intervals. Left: a window not significantly divergent from the neutral estimate; right: a window significantly divergent from the neutral estimate, and with slightly higher relative reduction in $M_{12}$ than in $M_{21}$

We inferred selection by contrasting the locus‐specific migration rates $M_{12}$ and $M_{21}$ against their neutral estimates ${\hat{M}}_{12}$ and ${\hat{M}}_{21}$ (Figure 3). We evaluated the performance of our LSD implementation at identifying selected loci under these simulations by plotting the true positive rate (TPR) against the false positive rate (FPR) under the choice of HPDI thresholds from 0 to 1, and reporting the area under the curve (AUC) of the resultant receiver operating characteristic (ROC) curve (approximated by the Mann–Whitney *U* test; Delong et al., 1988). An AUC value of 0.5 reflects random assignment while that of 1 reflects perfect classification. To evaluate the accuracy of the inferred symmetry of the joint posterior (of reciprocal migration rates), we compared it to the true underlying selection coefficients, under the expectation that deviations from symmetry in the joint posterior should reflect asymmetry in selection regimes. Specifically, we determined for each locus l the posterior mass

$$\sigma_{l}=\int \text{Ind}\left(\frac{M_{21}}{M_{12}}<\frac{{\hat{M}}_{21}}{{\hat{M}}_{12}}\right)\pi_{l}\left(\theta \right)d_{\theta },$$

where the indicator function $\text{Ind}\left(\cdot \right)$ limits the integral to cases in which the deviation of one of the migration rates has reduced more than the reciprocal migration rate compared to a proportional deviation of both migration rates from their neutral estimates ${\hat{M}}_{12}$ and ${\hat{M}}_{21}$. From this, we calculate the asymmetry as

$$a=\log\frac{\sigma }{1‐\sigma },$$

where $\sigma =\frac{1}{n_{s}}\sum \sigma_{l}$ across loci simulated under selection.

AUC and asymmetry are reported for each simulated pseudogenome, each representative of a unique demographic‐selection regime.

#### 2.3.4 ABC parametrization

Migration rates were drawn from ${\text{log}}_{10}M_{12}$, ${\text{log}}_{10}M_{21}\sim U$[−4,3] in all cases, while all other parameters were fixed to their true values (“fixed” parametrization). We do this to assess the sensitivity and accuracy of LSD under ideal conditions, and to avoid confounding with model and parameter mis‐specifications. For ABC parameter estimation, we retained the 1% closest simulations of 250,000 total simulations.

#### 2.3.5 Comparison with other methods

We compared the performance of LSD against that of two widely employed genome scan methods, on the simulated pseudogenomes. pcadapt (Luu et al., 2017) identifies candidate single nucleotide polymorphisms (SNPs) as outliers with respect to population structure, ascertained via PCA, while outflank (Whitlock & Lotterhos, 2015) infers candidate SNPs by testing against a null model inferred from a highly revised Lewontin–Krakauer model extended to account for nonindependent sampling of populations and sampling errors. Given that these approaches are SNP‐based (whereas LSD is window‐based), we consider a specific genomic window to be an outlier if at least one SNP within that window is called significant. This may reduce false negatives at the expense of inflating false positives, but reflects typical usage of such genome scans. For both methods, the true number of simulated populations was specified and SNPs were thinned to accommodate the particular LD structure of our simulated pseudogenomes when computing PCs and calibrating the *F*_ST_ null distribution, before running on the full SNP dataset.

For a more realistic comparison where model parameters may not be known with confidence, we also considered cases of $M_{1}$ and $M_{4}$ in which all demographic parameters were unknown (“free” parametrization) and drawn from large, uniform priors ${\text{log}}_{10}N_{i}\sim U$[2,7], *i* = 1,2 for $M_{1}$ and ${\text{log}}_{10}N_{i}\sim U$[1.5,5], *i* = 1, …, 4, ${\text{log}}_{10}T_{\mathrm{Dj}}\sim U$[4.1,5.6], *j* = 1,2,3, ${\text{log}}_{10}\alpha \sim U$[0,1] and ${\text{log}}_{10}M_{\mathrm{adj}}\sim U$[0,1] for model $M_{4}$. Priors for $M_{12}$, $M_{21}$ remained the same as before. We used the closest 10,000 out of 1,000,000 simulations both to infer $\hat{\theta }$ and to identify outlier loci. For added realism, we inferred $\hat{\theta }$ from a full pseudogenome of 1050 loci, of which 1000 were neutral but 50 were mis‐specified and weakly selected with $s_{1}$, $s_{2}$ = 0.001; $T_{s}=4000$, $M_{12}=M_{21}=M$.

### 2.4 Case study

To evaluate the performance of LSD on real data, we applied it to the detection of loci underlying floral colour in two parapatric subspecies of *Antirrhinum majus*. *A. majus* is an herbaceous, perennial, flowering plant native to the western Mediterranean. Owing to its diploid inheritance, relatively short generation time, ability for both self‐ and cross‐pollination, and rich and varied flower morphology, *A. majus* has lent itself as a model organism for over a century, with several key floral genes being first identified within this genus (Schwarz‐Sommer et al., 2003; Schwinn et al., 2006). Two subspecies, *A*. *m*. *striatum* and *Antirrhinum majus pseudomajus*, differ in the flower colouration that signposts the pollinator entry point, and form a natural hybrid zone in the Pyrenees that constitutes a benchmark example of divergent selection (Whibley et al., 2006). Several genetic loci have been shown to control the differences in these floral patterns (Bradley et al., 2017; Schwinn et al., 2006), and recently, Tavares et al. (2018) produced evidence of genomic signatures of selection at the ROSEA (ROS) and ELUTA (EL) loci, of which the former was functionally validated. Here, we apply LSD to sequencing data from this study to isolate the ROS and EL loci and to characterize their underlying selection signal. We filtered the data as in the original study, but mapped on a more recent version of the *A*. *majus* reference (version 3.0; Li et al., 2019).

We modelled this study system via a simple representation (model $M_{1}$) of one population on either side of the hybrid zone (YP1 [*A*. *m*. *striatum*] vs. MP2 [*A. m. pseudomajus*]; populations 2.5 km apart) and via a more inclusive island–continent model (model $M_{2}$) comprising three (distant) populations each per subspecies (CAM, ML, YP1 [*A*.* m*. *striatum*] vs. MP2, CHI, CIN] *A. m. pseudomajus*]; Figure S4), using an estimate of *µ* = 1.7 × 10^−8^ per bp per generation (Tavares et al., 2018) and allowing all *N* and *M* parameters to be free with priors ${\text{log}}_{10}N_{j}\sim U$[1,7], *j* = 1,2 and ${\text{log}}_{10}M_{12}$, ${\text{log}}_{10}M_{21}\sim U$[−4,4] for $M_{1}$ and ${\text{log}}_{10}N_{j}\sim U$[3,7], *j* = 1,2 and ${\text{log}}_{10}N_{\mathrm{ik}}\sim U$[1,6], *k* = 1, …, 6, ${\text{log}}_{10}M_{12}$, ${\text{log}}_{10}M_{21}\sim U$[−4,4] and ${\text{log}}_{10}M_{\mathrm{ic}}\sim U$[0,5] for $M_{2}$. In line with the available pool‐seq data for this study, we simulated pooling of individuals in silico by pooling twice the amount of msms coalescent (haploid) samples as (diploid) individuals in the pooled populations via lsd‐high. Reads were then drawn from a parametric (negative binomial) distribution fitted to the empirical coverage distribution using the “*fitdistrplus*” package (Delignette‐Muller & Dutang, 2015) and lsd‐high. We focused our analysis on chromosome 6 on which the ROS and EL loci lie. To acquire empirical estimates of neutral demographic parameters, we excluded all genomic regions present in the structural annotation plus 10‐kb flanking regions to generate a subset of putatively neutral regions on that chromosome. ${\hat{M}}_{12}$ and ${\hat{M}}_{21}$ were then estimated via 10‐kb windows from these neutral regions by retaining the closest 10,000 out of 1,000,000 simulations, as outlined above. To identify selected loci in the second step, we used sliding windows of size 10 kb and a 1‐kb step‐size, and retained the closest 5000 out of the same 1,000,000 simulations. Window size was identical to that of Tavares et al. (2018) and was chosen to reflect a compromise between statistical power and resolution (genomic signatures of selection were previously found to be in the range of ~20–40 kb; Tavares et al., 2018), as well as to be compatible with the system's LD, which is expected to decay rapidly in outcrossing populations of *A*. *majus*.

## 3 RESULTS

### 3.1 Two‐deme IM case (model $M_{1}$)

#### 3.1.1 Power to identify selected loci

**FIGURE 4:**
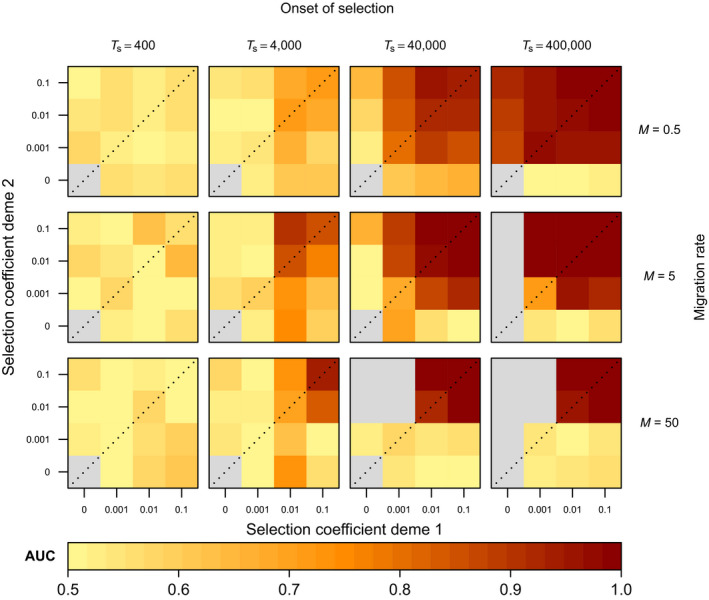
Simulation results showing the effect of migration rate, time of onset of selection and deme‐specific selection coefficients on LSD diagnostic performance (AUC), for the two‐deme IM model (model $M_{1}$; standing genetic variation case). Each cell represents a pseudogenome simulated under a specific selection regime. The cell colours reflect the AUC calculated by the correct discrimination of 1000 neutral loci and 50 selected loci in the 1050 loci simulated pseudogenomes. Grey cells indicate selection regimes where the derived allele is always lost

While our LSD implementation exhibited conservative *p_l_* values (Figure S5), it demonstrated a high diagnostic ability to discriminate between neutral and selected loci (AUC > 0.8) across a large range of migration‐selection regimes (Figure 4). Notably, our results point towards an optimal, intermediate rate of migration (M=5) at which selection is best detectable with high AUC values across a large set of selection coefficients. As migration rates increase (M=50), migration from the foreign deme where selection acts on the alternate allele increasingly inhibits the build‐up of beneficial polymorphisms in the local deme, in which case the power to detect selected loci becomes limited to scenarios under longer regimes of strong selection. At lower migration rates (M=0.5), long regimes of selection permit the detection of loci under the lowest selection coefficients, but power decreases for younger times compared to scenarios simulated under intermediate migration rates. This owes to LSD relying on the reduction of effective migration relative to neutral or genome‐wide expectations, which in this case is already at a low level.

The power to detect selection increased with increasing selection coefficients when these were similar ($s_{1}\approx s_{2}$, cells along diagonal of subpanels in Figure 4). In such cases, stronger selection coefficients on alternate alleles increasingly polarize and ultimately maintain larger allele frequency differences between the two environments. In tandem, the power to detect selection also generally increased with the time since the onset of selection $T_{S}$. In contrast, when $s_{1}\gg s_{2}$ or $s_{1}\ll s_{2}$, one of the two alleles may proceed to fixation, in which case the power to detect selection decays or is lost (e.g., grey cells in left‐most column of subpanels when derived allele *A* is lost, and AUC values and inferred (a)symmetries tending toward 0.5 and 0 respectively in bottom row cells when ancestral allele *a* is lost; Figure 4). This is particularly evident when the onset of selection is more distant in the past.

#### 3.1.2 Power to characterize (a)symmetry

**FIGURE 5:**
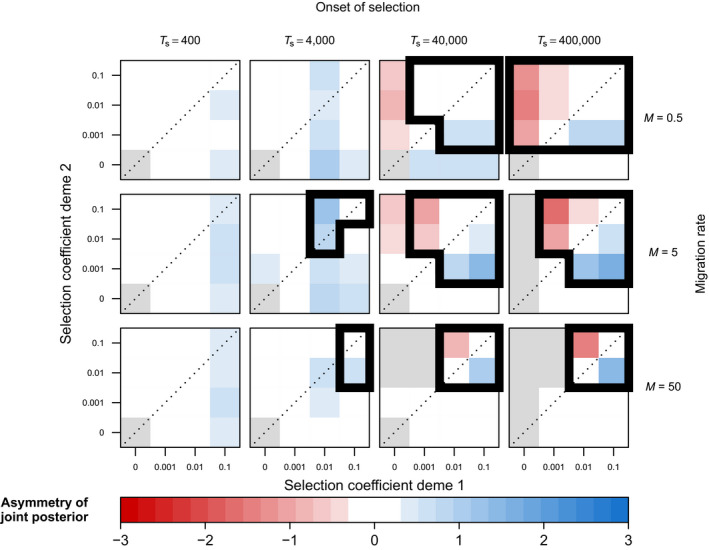
Simulation results showing the effect of migration rate, time of onset of selection and deme‐specific selection coefficients on LSD‐inferred (a)symmetry of selection, for the two‐deme IM model (model $M_{1}$; standing genetic variation case). Each cell represents a pseudogenome simulated under a specific selection regime. The cell colours reflect the (a)symmetry values inferred by LSD, where a value of 0 reflects perfect symmetry of the joint posterior while values divergent from this reflect asymmetry. Cells surrounded by thick lines indicate the values of (a)symmetry for regimes expected to generate meaningful signal (AUC > 0.8 in Figure 4). Grey cells indicate selection regimes where the derived allele is always lost

A benefit of LSD over classical outlier approaches is that it can provide insight into genetic trade‐offs underlying local adaptation, by identifying cases in which selection acts at equal strength in the two demes or metapopulations (symmetric AP), or whether selection coefficients differ considerably (CN or asymmetric AP). As shown in Figure 5, the inferred (a)symmetry generally reflected the true (a)symmetry of the underlying selection coefficients well, particularly for regimes with high power to correctly identify selected loci (Figure 4). In lower powered regimes, we observe some cases where the inferred asymmetry does not reflect the underlying asymmetry of the selection coefficients accurately (e.g., blue cells along diagonals in subpanels at $T_{S}=4000$; Figure 5; Figure S6B). We interpret these results further in the discussion.

#### 3.1.3 Standing variation vs. de novo

A lower initial frequency of the derived allele may be expected to affect LSD’s power to identify selected loci and its power to capture the underlying (a)symmetry of selection coefficients. However, we find that results for simulations building on selection from the de novo and standing variation cases showed generally very similar patterns (Figures 4 and 5; Figure S6). One notable exception however was the inaccurate inference of (a)symmetry in a few regimes with high power (AUC > 0.8) in the de novo case (e.g., blue cells along diagonals in subpanels at $T_{S}=4000$ in Figure S6B). This we attribute to the lower initial frequency of the derived allele *A* and consequently longer time needed to approach drift–migration–selection equilibrium for the de novo cases. This is explored further in the discussion.

### 3.2 More complex cases (models $M_{2}$, $M_{3}$ and $M_{4}$)

**FIGURE 6:**
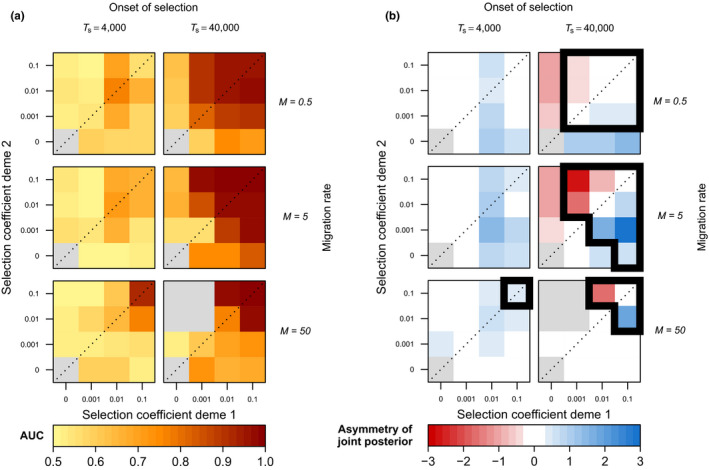
Simulation results for a two‐deme divergence with bottleneck and exponential growth model (model $M_{3}$; standing genetic variation case) showing the effect of migration rate, time of onset of selection and deme‐specific selection coefficients on (a) LSD diagnostic performance (AUC) and (b) LSD‐inferred (a)symmetry of selection. Divergence time of the two populations, $T_{D}$, is 200,000 generations ago. Each coloured cell represents a pseudogenome simulated under a specific selection regime. Grey cells indicate selection regimes where the derived allele is always lost. (b) Cells surrounded by thick lines indicate the values of (a)symmetry for regimes expected to generate meaningful signal (AUC > 0.8 in Figure 6a)

A key feature of LSD is its potential to explicitly accommodate complex demographies, which can lead to an inflation in false positives when not properly accounted for (Foll & Gaggiotti, 2008; Lotterhos & Whitlock, 2014; De Villemereuil et al., 2014). Despite the added complexity of models $M_{2}$ and $M_{3}$, results were generally very similar to that of model $M_{1}$, with high power to identify selected loci (AUC > 0.8) across a large range of migration–selection regimes, an optimal migration rate at an intermediate value (M=5), a similar dependence of power to detect selection on $s_{1}$, $s_{2}$ and $T_{S}$, and inferences of (a)symmetry that reflected well the underlying (a)symmetry of selection coefficients (Figure 6; Figures S7 and S8). One notable difference between these models was that model $M_{2}$ generally required a longer time to generate power to detect selection when compared to models $M_{1}$, $M_{3}$ and $M_{4}$, which we attribute to the larger metapopulation $N_{E}$ in model $M_{2}$. For model $M_{4}$, selection coefficients needed to be slightly higher ($s_{1}$, $s_{2}\geq 0.01$; Figure S8) to attain high power to identify selected loci, and to accurately identify asymmetry.

### 3.3 Robustness to mis‐specification of the neutral set

**FIGURE 7:**
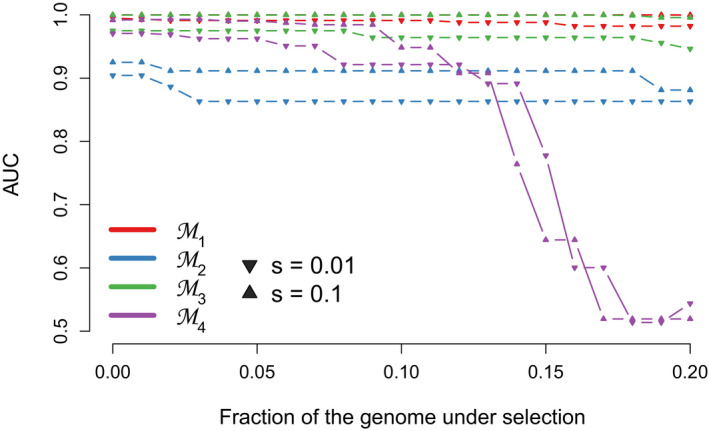
Effect of increasing fraction of mis‐specified (=selected) windows among the neutral set on LSD’s power to detect selection, for all four models and intermediate and high selection coefficients (*s*_1_ = *s*_2_ = *s* = 0.01, 0.1; *T_S_* = 40,000), and under neutral migration rates *M*_12_, *M*_21_ = 5. Here the neutral set of 1000 loci comprise a fraction *f* of selected loci and a fraction 1 − *f* neutral loci, with 0.0 ≤ *f* ≤ 0.2. The selected loci comprising the pseudogenome under scan are under the same selection regime as those included as mis‐specifications in the neutral set

As shown for all models and a subset of the regimes (M=5; $T_{S}=40,000$; $s_{1}=s_{2}=0.01, 0.1$), LSD is highly robust to the inclusion of selected loci in the neutral set, with negligible reduction in power (AUC) at up to a 13% mis‐specification for model $M_{4}$ and up to 20% for models $M_{1}$, $M_{2}$ and $M_{3}$ (Text S5; Figure 7).

### 3.4 Comparison to other methods

**FIGURE 8:**
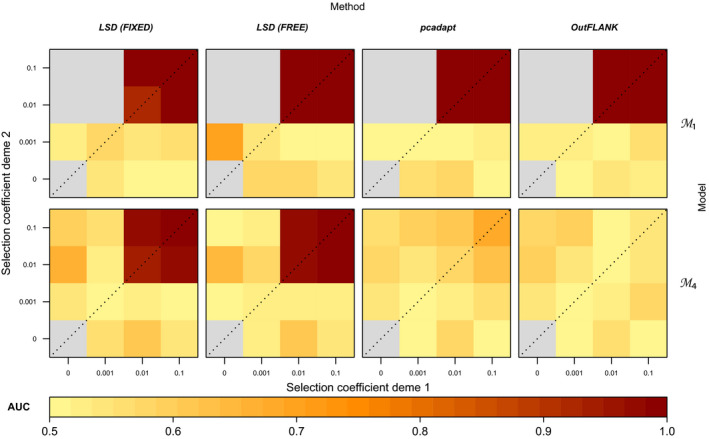
Comparison of power to detect selection (AUC) between LSD, pcadapt and outflank. Simulation results are shown for a simple ($M_{1}$) and a complex ($M_{4}$) demographic model and for a subset of the tested migration–selection regimes at *T_S_* = 40,000 and *M* = 50 (full results in Supporting Information). For LSD, two parametrizations are performed: (i) assuming non‐*M* demographic parameters and neutral $\hat{M}$ to be fixed to the true values (LSD FIXED) and (ii) allowing non‐*M* demographic parameters to be drawn from large prior ranges and using neutral $\hat{M}$ estimated from the pseudogenomes (LSD FREE). Grey cells indicate selection regimes where the derived allele is always lost

The performance of LSD is comparable to that of pcadapt and outflank under model $M_{1}$, with minimal difference between the methods across the range of migration–selection regimes (Figure 8; Figure S9). For the complex model $M_{4}$, however, both pcadapt and outflank have little to no power to correctly identify selected loci (AUCs ~ 0.5; Figure 8; Figure S10), while LSD exhibits high AUC scores (> 0.8) in similar migration–selection regimes as it does under models $M_{1}$, $M_{2}$ and $M_{3}$. Importantly, the power of LSD to identify loci under selection was very similar when fixing ${\hat{M}}_{12}$ and ${\hat{M}}_{21}$ and all demographic parameters not affected by selection to their true value (“fixed” parametrization), as when estimating ${\hat{M}}_{12}$ and ${\hat{M}}_{21}$ (Figures S11 and S12) and keeping all other parameters free (“free” parametrization) (Figures 4 and 8; Figures S8A–S10A), implying that LSD is robust to parameter specification.

### 3.5 Case study results

**FIGURE 9:**
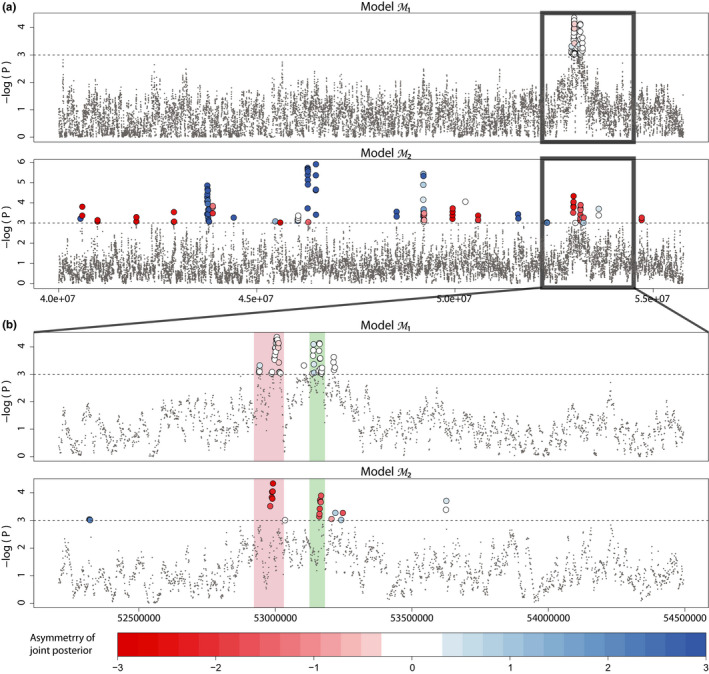
Manhattan plot for the LSD scan of the *Antirrhinum majus striatum–A. m. pseudomajus* system. The *p*‐values for loci being divergent from neutrality for 10‐kb windows (1‐kb step‐size) are plotted for (a) a 16‐Mb region of chromosome 6, under models $M_{1}$ and $M_{2}$, and (b) a 2‐Mb zoomed‐in region of chromosome 6 focusing on the ROS–EL region, under the same two models. The horizontal dashed line indicates a 99.9% posterior probability of deviating from neutral expectations. Colour for loci above this threshold denotes the joint (*M*_12_, *M*_21_) posterior (a)symmetry, and reflects the relative strengths of selection in the two divergent demes or subspecies. A large divergent peak centred around the ROS–EL region (a) is composed of a set of smaller peaks (b), consistent with the ROS (red) and EL (green) loci

We identified a region of reduced effective migration between 52.9 and 53.2 Mb on chromosome 6 (Figure 9), consistent with the location of the ROS and EL loci (Tavares et al., 2018). Under model $M_{1}$, this region is characterized by a set of smaller, multiple peaks ($p_{l}<0.01$) reflecting signatures identified by previous authors, with the left‐most peaks corresponding to ROS1 and ROS2 (shaded in red) and the right peaks to EL (shaded in green; Figure 9b). The joint posterior probability distributions reveal symmetric selection acting on both regions, implying that selection acts with similar strength in the two populations. Under model $M_{2}$, we find fewer outliers in the ROS–EL region than in model $M_{1}$, with the left‐most peak in this region corresponding to ROS2 and the right peaks consistent with EL. ROS1 appears to be less of an outlier than in model $M_{1}$ ($p_{l}\approx 0.02$). In contrast to model $M_{1}$, the ROS2 and EL peaks in model $M_{2}$ are characterized by asymmetry, specifically with stronger selection acting in the populations of *A. m. pseudomajus* than in the populations of *A*.* m*. *striatum*. Estimates for $\hat{\theta }$ for both models are given in Figures S13 and S14.

## 4 DISCUSSION

The trajectories of selected loci depend on demographic and selection parameters that define the system, namely the effective population sizes, effective migration rates, selection coefficients and times of onset of selection, as well as on the intrinsic properties of mutation and recombination. Despite well‐developed theory which relates the effect of population parameters on the trajectories of selected alleles, few methods or empirical studies have combined estimates of differential selection with explicit quantification of migration rates and effective population sizes to examine the conditions under which local adaptation can arise. Here, we introduce such a method, LSD, which identifies candidate loci based on divergent population parameters using explicit demographic models, and demonstrate that under certain demographic‐selection regimes, it can both detect and elucidate the processes underlying signatures of selection. While LSD is flexible regarding the choice of demographic models employed and can be applied to single and multiple populations, we focus here specifically on processes that lead to selection against gene flow, namely local adaptation and extrinsic reproductive barriers, that can be inferred via their expectation to reduce *M*_E_.

### 4.1 Identifying selection

Using simulations, we demonstrate that LSD has high diagnostic power (AUC > 0.8) to identify selected loci across a large range of demographic‐selection regimes. This power relies upon two fundamental aspects that contribute to generating observable patterns. First, selection must effectively be realized (i.e., result in a frequency shift of the beneficial allele). This requires that the strength of selection and initial frequency of the beneficial allele be sufficient to both counter the homogenizing effect of migration (Felsenstein, 1976; Haldane, 1930; Lenormand, 2002; Olson‐Manning et al., 2012; Slatkin, 1973; Yeaman, 2015) and the eroding effect of drift (Wright, 1931). Second, the genomic data must contain signatures of selection that can be detected. In the case of LSD, this requires that the signatures of selection are discernible from the underlying noise (drift and migration) that characterizes the system, which demands sufficient time for said signatures to be reflected in the employed statistics and hence in the inferred parameters *N*_E_ or *M*_E_. A lack of power in LSD must be interpreted considering these two conceptually different aspects. Notably, the lack of discrimination power for high migration rates and low selection coefficients can be attributed to selection failing to realize as a consequence of local, beneficial alleles being swamped by immigrant, maladaptive alleles. In contrast, the lack of signal under low migration rates constitutes a methodological limitation of our implemented model, as it becomes increasingly difficult to detect reductions in effective migration when neutral or genome‐wide migration rates are already at a low level, even when selection is effectively being realized in the demes. This is analogous in effect to the loss of power to detect selection in highly differentiated populations in *F*_ST_ outlier tests (Hoban et al., 2016; Martin et al., 2013). Under the same principle, we argue that the converse expectation can be assumed to hold for loci underlying adaptive introgression or balancing selection. That is, we expect power to detect such loci to be low when populations are minimally differentiated and high in highly divergent systems, as the detection of candidate loci in these cases is informed by increased effective migration.

The power of LSD to correctly identify selected loci generally increases with stronger selection coefficients and longer time since the onset of selection, though with exceptions. Specifically, if selection is of similar or equal strength in both demes or metapopulations, we observe a strong correlation between the power to detect selection and the true underlying selection coefficients. This follows theory which states that the reduction in effective migration is proportional to the strength of selection (Petry, 1983). However, we defer from translating these changes to explicit selection coefficients because in addition to the strength of selection, changes in effective migration are also a function of the recombination rate between linked and selected loci (Cutter & Payseur, 2013; Lotterhos, 2019; Petry, 1983). If selection differs strongly between demes or metapopulations ($s_{i}\gg s_{j}$) or when the onset of selection is sufficiently distant in the past, however, one allele may become fixed in the system. In such a case, the signal to detect selection rapidly decays (Huber et al., 2016; Przeworski, 2002). Moreover, we observed little power to detect very recent selection, intrinsically related to our choice of summary statistics (Hohenlohe et al., 2010). That said, extending LSD to include additional statistics sensitive to linkage disequilibrium such as EHH (Sabeti et al., 2002) or single density score (SDS) (Field et al., 2016) will increase the power to detect more recent selection. Finally, given that LSD relies on localized deviations in effective demographic parameters, we predict that it may miss signals of polygenic selection, as signals of selection become increasingly hard to distinguish from the genomic background the smaller the locus effect sizes are.

From our simulations, we find that the power to detect selection is similar between the de novo and standing genetic variation regimes (model $M_{1}$; Figure 4; Figure S6A), likely as a result of only considering loci for which the derived allele was not lost. This particularly affected the de novo case, under which the derived allele was lost in most simulations. Indeed, this is in line with theoretical expectation (Olson‐Manning et al., 2012) and supports the notion that most empirical cases of local adaptation attribute selection of advantageous alleles to arise from standing variation (Jones et al., 2012; Lai et al., 2019; Reid et al., 2016). To clearly distinguish between these regimes, additional information on the evolutionary history of the system such as allele age, mutation rate or supplementary phylogenetic information is required (Peter et al., 2012).

### 4.2 Comparison to existing methods

LSD’s power to detect selection is comparable with that of pcadapt and outflank under the simple model $M_{1}$, but strongly outperforms these alternatives under the more complex model $M_{4}$. These results appear to hold regardless of whether prior knowledge of model parameters is confidently known for LSD, as LSD exhibits similar performance under the ideal “fixed” and realistic “free” parametrizations. This suggests that the methods pcadapt and outflank utilize to address population structure, namely PCA and an implicitly modelled *F*_ST_ null distribution, respectively, are sufficient to control for the effects of relatively simple demography, but insufficient to capture more complex demographies. By modelling complex demographies explicitly, LSD is less affected by model complexity, though this extra power comes with some costs.

First, a demographic model needs to be specified that appropriately describes the neutral genetic variation of the system, allows for inferences of selection through changes in demographic parameters (e.g., $N_{E}$ or $M_{E}$), and is sufficiently simple to remain computationally tractable. A preliminary analysis of model choice may therefore constitute a prerequisite to successfully recapitulate the signal of complex evolutionary histories in the simulated data. Importantly, the model should always be validated by demonstrating that the observed data can be accurately and sufficiently captured (Figure S15).

Second, LSD remains computationally more demanding than both pcadapt and outflank. Inferring demographic parameters is generally computationally challenging as the underlying genealogies need to be integrated out numerically (Hey & Nielsen, 2007), which for complex models usually requires simulation‐based approaches such as ABC. Existing ABC approaches to infer locus‐specific parameters (Bazin et al., 2010; Kousathanas et al., 2016) are difficult to scale‐up to genome‐wide data as they require the simulation of prohibitively many loci. To circumvent this problem, LSD implements an efficient ABC approach that requires simulations of single loci only, which is possible because LSD neither attempts to infer the hierarchical distribution of locus‐specific parameters nor to obtain posterior estimates on whether a locus is affected by selection. Instead, LSD identifies loci under selection by quantifying whether locus‐specific estimates of demographic parameters are incompatible with those estimated from a set of putatively neutral loci.

The a priori identification of this neutral set constitutes the third requirement. Such a set may be informed by the particular structural or functional class the sites belong to (Williamson et al., 2005) and may for instance consist of genomic regions not linked to structural annotations. Alternatively, a more naïve strategy may rely on the whole genome or a random subset of the genome to reflect neutral diversity. Even if this assumption of neutrality is violated (Begun et al., 2007; Fay et al., 2002; Li & Stephan, 2006), we show that our method to estimate $\hat{\theta }$ is robust to the misidentification of neutral loci, with minimal effect on power even at high percentages of up to 20% mis‐specification. We attribute this to our method of estimating $\hat{\theta }$ as the product of per‐locus posterior densities, which amplifies the signal (density) according to majority rule.

We finally note that most widely applied genome scan methods (e.g., pcadapt and outflank) detect outliers at the SNP level, while our current implementation of LSD focuses on genomic windows. Focusing on genomic windows offers a means to aggregate information across linked loci, thereby potentially increasing power and reducing false positives from spurious signals at individual SNPs (e.g., Galimberti et al., 2020). These benefits are conditional on a choice of window size compatible with the LD decay in the system, as this maximizes the power and accuracy to capture the signal generated by selection. The LSD framework is by no means limited to genomic windows, however, and can be extended to SNP data when using an appropriate simulator and summary statistics calculator.

### 4.3 Revealing trade‐offs underlying selection

**FIGURE 10:**
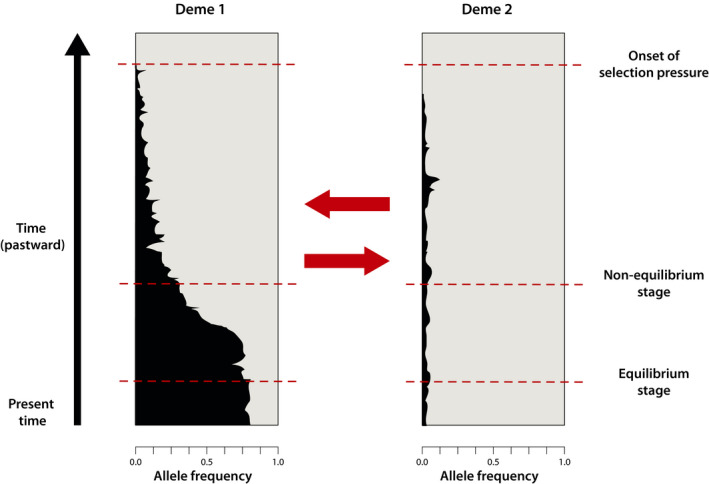
Conceptual illustration of allele frequency trajectories over time in a two‐deme IM model ($M_{1}$), for an example de novo case and antagonistic pleiotropic selection regime. The frequency of derived allele *A* is indicated in black and that of ancestral allele *a* in grey. Red arrows represent migration. Prior to reaching drift–migration–selection equilibrium, estimated asymmetries in effective migration rates are also affected by asymmetry in allele frequencies

LSD can shed light on the directionality of selection by inferring the (a)symmetry in deviations of migration rates between populations. In our simulations, this inferred (a)symmetry accurately reflects the (a)symmetry in the underlying selection coefficients for older onsets of selection, but less so for more recent onsets (Figures 5 and 6B; Figures S6B and S7B). This is because inferred asymmetries in deviations of effective migration rates are also affected by asymmetries in allele frequencies of the beneficial allele. For instance, a new beneficial mutation that arises in one of two demes will initially be rare among migrants. Only as its frequency increases will selection start to act against immigrants in both demes (Figure 10). Hence, the inference of asymmetry in LSD may include cases where selection is effectively asymmetric, but also those in which selection is effectively symmetric but prior to drift–migration–selection equilibrium. A direct link between the inferred (a)symmetry in deviations of migration rates and the (a)symmetry in underlying selection coefficients is only established through time. We note that in practice, however, the interpretation of the results is straightforward, as the inference of directionality is only meaningful for loci identified as under selection (i.e., with low *p_l_* values). Such loci can only be inferred in regimes with high AUC, which generally preclude regimes characterized by recent onsets of selection. For these (high AUC) regimes, we generally find the estimated (a)symmetries to reflect the true (a)symmetry in selection coefficients accurately. Stated succinctly, LSD’s inferred directionality is generally accurate and meaningful for identified candidates, and potentially inaccurate but irrelevant for other loci.

The ability of LSD to infer the directionality of selection directly from genomic data can greatly facilitate investigations of genetic trade‐offs underlying adaptation, which are seldom performed due to the considerable effort required to set up field trials of recombinant lines. As shown above, the inference of symmetry in LSD‐identified candidates accurately reflects cases of AP with equal strength of selection on alternate alleles in the contrasting environments. The inference of asymmetry, on the other hand, can either indicate AP with stronger selection in one environment than the other, or CN. From our simulations, we find that scenarios reflecting AP are generally more readily detected than those reflecting CN. Given that selection acts only upon one of the two alleles in the latter case, fixation becomes likely and the ability to detect selection is transient. This implies that there may be an observation bias between AP and CN, such that the inference of CN may be comparatively under‐represented. This bias appears to contrast with that reported in the ecological literature, where instances of AP are more rarely detected compared to CN due to the additional power required to statistically prove differential fitness concurrently in two environments (Anderson et al., 2013). LSD may further complement field trials as such experiments typically test genetic trade‐offs under contemporary selective environments, which may not reflect past conditions driving the observed adaptive responses, but whose signature may still be inferred from genomic data. Using LSD to formulate expectations about fitness effects and to inform the choice of environmental conditions under which to validate identified candidate genes can thus greatly aid such experiments.

### 4.4 Real‐world application

We demonstrate a real‐world application of LSD by successfully isolating and characterizing the selection signal of loci underlying an extrinsic reproductive barrier in *A*. *majus*. Our results from contrasting a single population (model $M_{1}$) and three populations (model $M_{2}$) per subspecies both identified the ROS and EL loci which were previously reported to underlie differences in floral patterns between these subspecies (Tavares et al., 2018). Interestingly, however, our results characterize selection at these loci as symmetric under model $M_{1}$ and asymmetric with stronger selection acting on *A*. *m. pseudomajus* than in *A*. *m. striatum* under model $M_{2}$. This exemplifies that results of LSD genome scans are conditional on the model and populations used, such that here, model $M_{1}$ uncovers population‐pair‐specific differences at the contact zone (YP1 vs. MP2) while model $M_{2}$ reveals common (global) differences between the two subspecies. We do not necessarily expect these two signals to be identical, and indeed, Tavares et al. (2018) showed different *θ*_W_ and *F*_ST_ estimates between distant and close *A. m. striatum*‐*A. m*. *pseudomajus* population pairs. Consistent with their results, we recovered a larger number of candidate loci in model $M_{2}$ (which comprise multiple, more distant populations), where isolation by distance underlies genome‐wide patterns in which the ROS–EL region no longer stands out exclusively. Given that there is no evident difference in environment or pollinators on opposite sides of the hybrid zone, reproductive barriers in this system have often been proposed to be maintained through selection against hybrids and frequency‐dependent sexual selection mediated by pollinator preference for the dominant flower phenotype on either side of the contact zone (Tavares et al., 2018). However, whether selection on alternate alleles follows the same positive frequency‐dependence across the broader scale, including more distant populations, is currently unknown. The difference in signal between local pairs at the contact zone ($M_{1}$) and the global set ($M_{2}$) may be generated by different frequency‐dependent selection curves for the alternate alleles and potentially loss of AP away from the contact zone (Figure S16).

## 5 CONCLUSION

Loci under selection are predicted to exhibit genealogies with demographic parameters divergent from those of neutral nonlinked regions, leading to heterogeneity in demography across the genome. In this study, we condition the identification of candidate loci on divergent population parameters using explicit demographic models, and demonstrate that under certain conditions of migration, selection strength and onset time, we can both detect and elucidate the underlying processes driving signatures of selection. Incorporating and utilizing the inference of demographic parameters in the identification of candidate loci address some key issues and assumptions that prevail in the discrimination of selected variants, namely (a) the explicit consideration of demography, (b) heterogeneity in drift and gene flow across the genome, (c) information synthesis of multiple, complementary summary statistics and (d) transparency towards underlying driving mechanisms.

Our power analysis using simulations shows that LSD, and our implementation of it, represents a powerful method for detecting selection that is robust to different and complex demographies. Furthermore, given that certain demographic parameters (e.g., migration) are not inherently commutative, we show that the directionality or population‐specificity in selection can be inferred. This can facilitate identifying in which environment selection acts and hence elucidate genetic trade‐offs, bridging an analytical divide between experimental ecology and population genomics. Importantly, the proposed approach as well as our implementation is not limited to the demographic models investigated here, nor the explicit choice of simulation programs or summary statistics used. This flexibility and customizability of LSD can facilitate, for example, more realistic accommodation of recombination (via different coalescent simulators), improved detection of more recent selection (via linkage‐informative statistics), and inference of other modes of selection (e.g., balancing selection) and adaptive introgression by conditioning the detection of selection on, for instance, an increase (rather than reduction) in *M*_E_ or changes in *N*_E_ relative to neutral expectations.

## AUTHOR CONTRIBUTIONS

H.L., D.W., A.W. and S.F. designed the study. H.L. wrote the LSD accessory programs and performed the simulations and analyses. H.L. and D.W. developed the methods. H.L. wrote the manuscript, which all authors critically revised.

## Supporting information

Supplementary Material

Fig S1‐S16

## Data Availability

We provide scripts to perform LSD genome scans at the GitHub repository: https://github.com/hirzi/LSD.
