# Supplementary material for: Identifying loci under selection via explicit demographic models
Source: Mol Ecol Resour. 2021 Jun 3;21(8):2719–37. doi: 10.1111/1755-0998.13415 (PMC8596768; doi:10.1111/1755-0998.13415)
Supplement: Supplementary file 2 — Fig S1‐S16 [file MEN-21-2719-s002.zip › Supplementary Figures/Figure_S8.pdf]

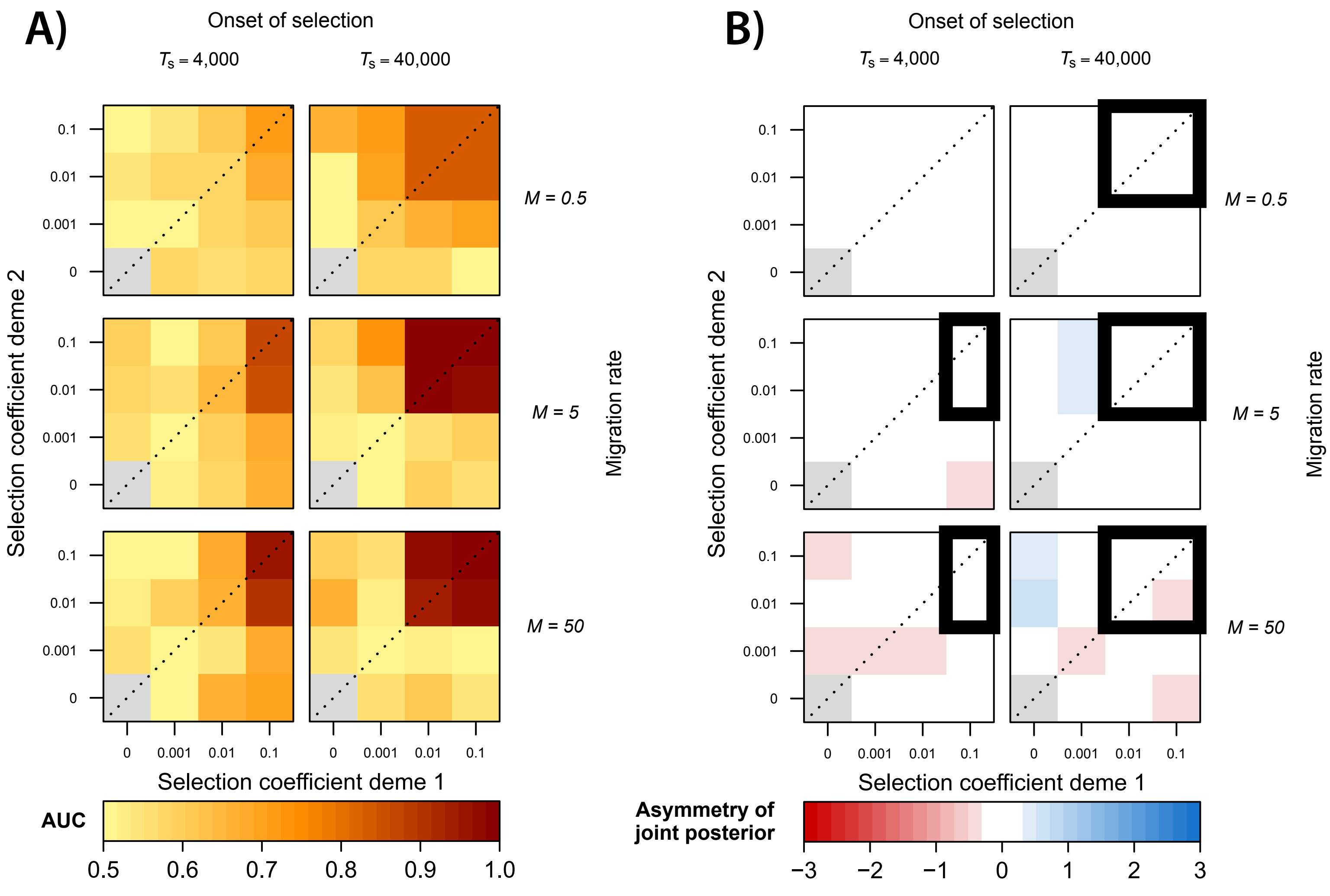

A)
Onset of selection
T_s = 4,000
T_s = 40,000
Selection coefficient deme 2
Selection coefficient deme 1
Migration rate
M = 0.5
M = 5
M = 50
0.1
0.01
0.001
0
AUC
0.5
0.6
0.7
0.8
0.9
1.0
B)
Asymmetry of joint posterior
−3
−2
−1
0
1
2
3
