# Supplementary figures and images for: Identifying loci under selection via explicit demographic models
Source: Mol Ecol Resour. 2021 Jun 3;21(8):2719–37. doi: 10.1111/1755-0998.13415 (PMC8596768; doi:10.1111/1755-0998.13415)

Posterior probability density

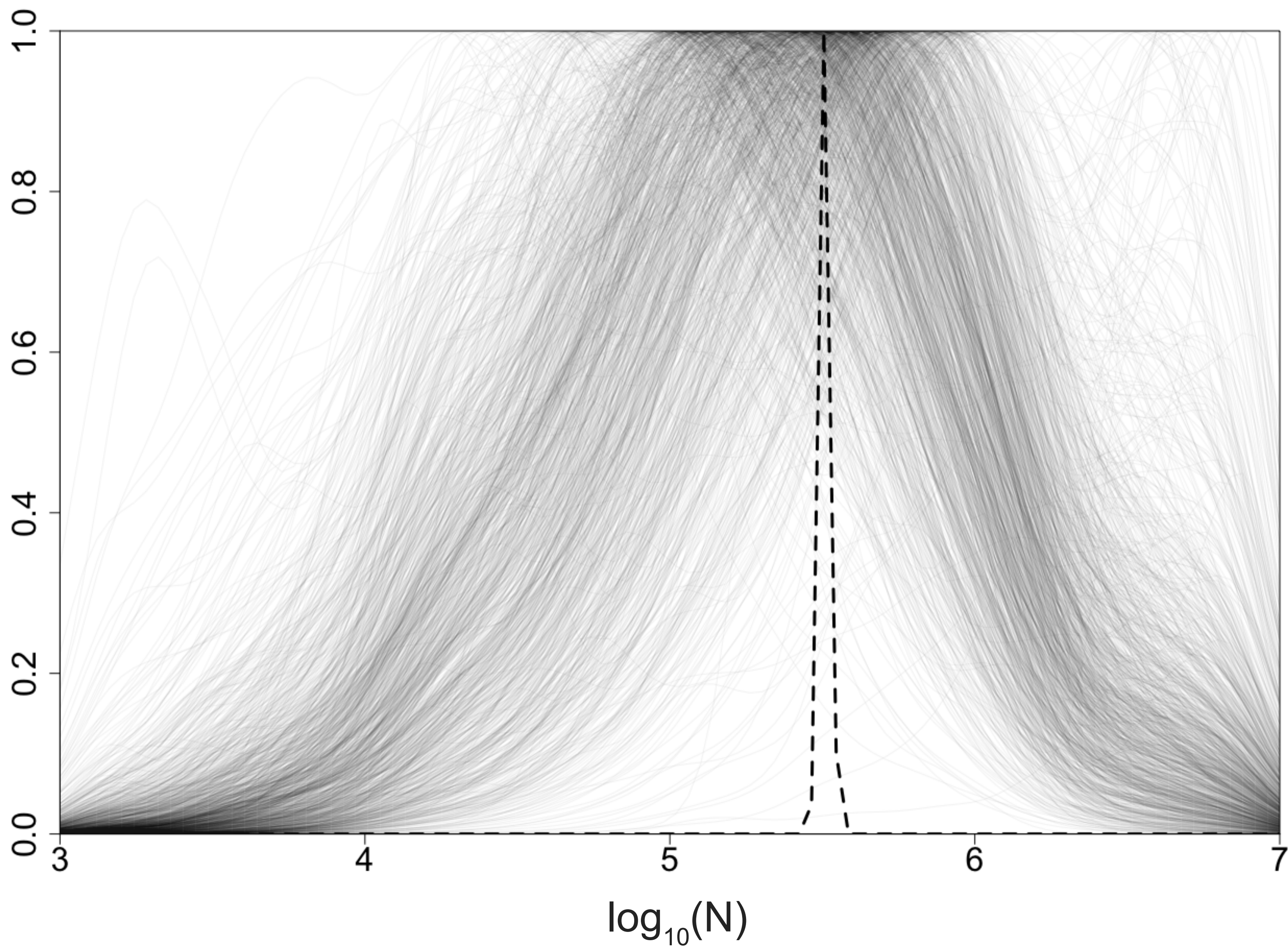

Supplement: Supplementary file 2 — Fig S1‐S16 [file MEN-21-2719-s002.zip › Supplementary Figures/Figure_S1.pdf]

**A)**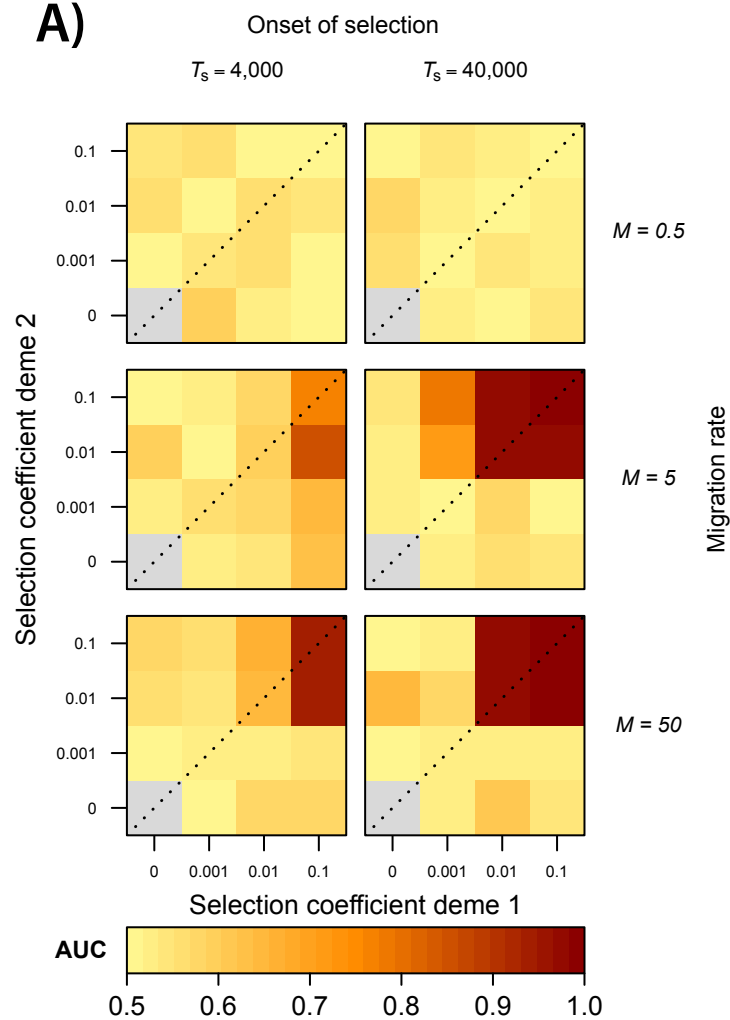**B)**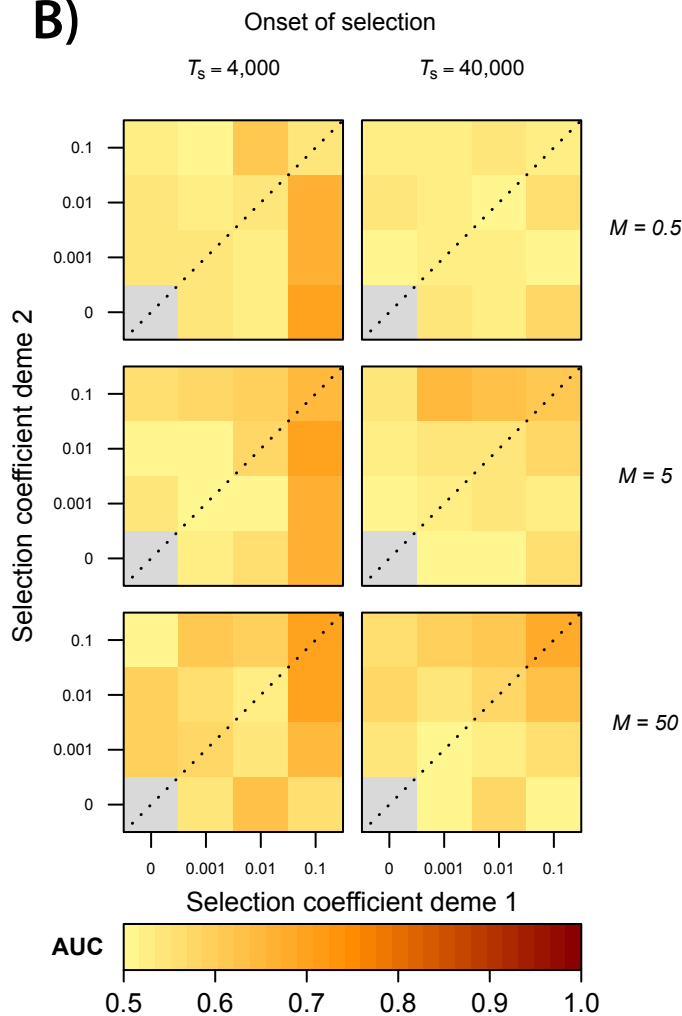**C)**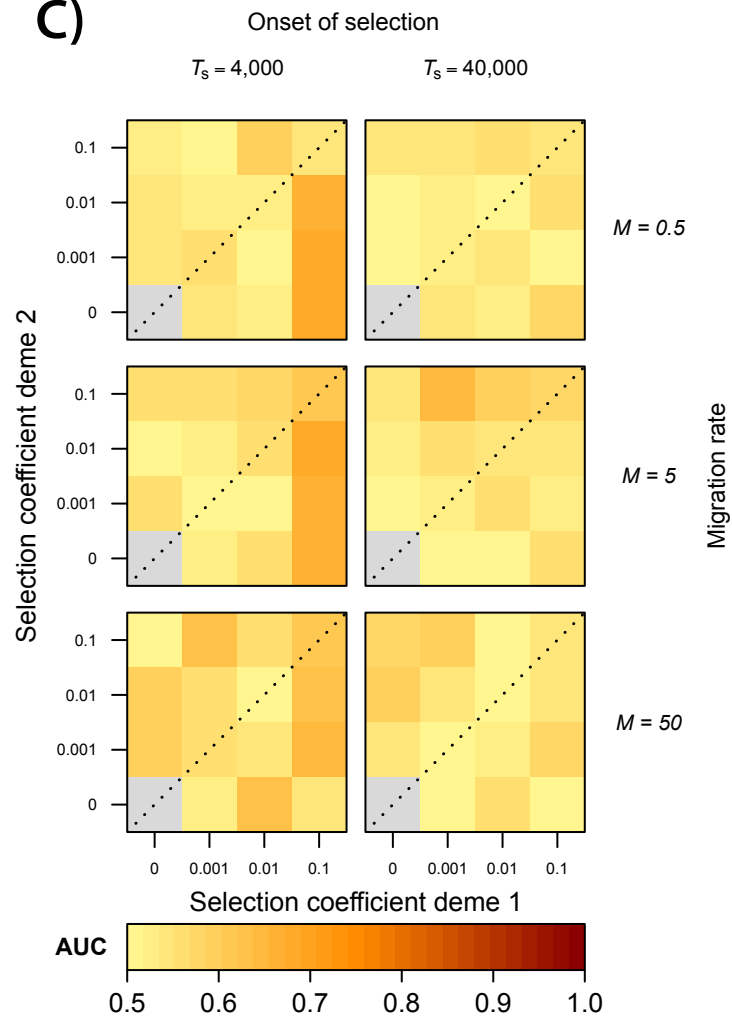

Supplement: Supplementary file 2 — Fig S1‐S16 [file MEN-21-2719-s002.zip › Supplementary Figures/Figure_S10.pdf]

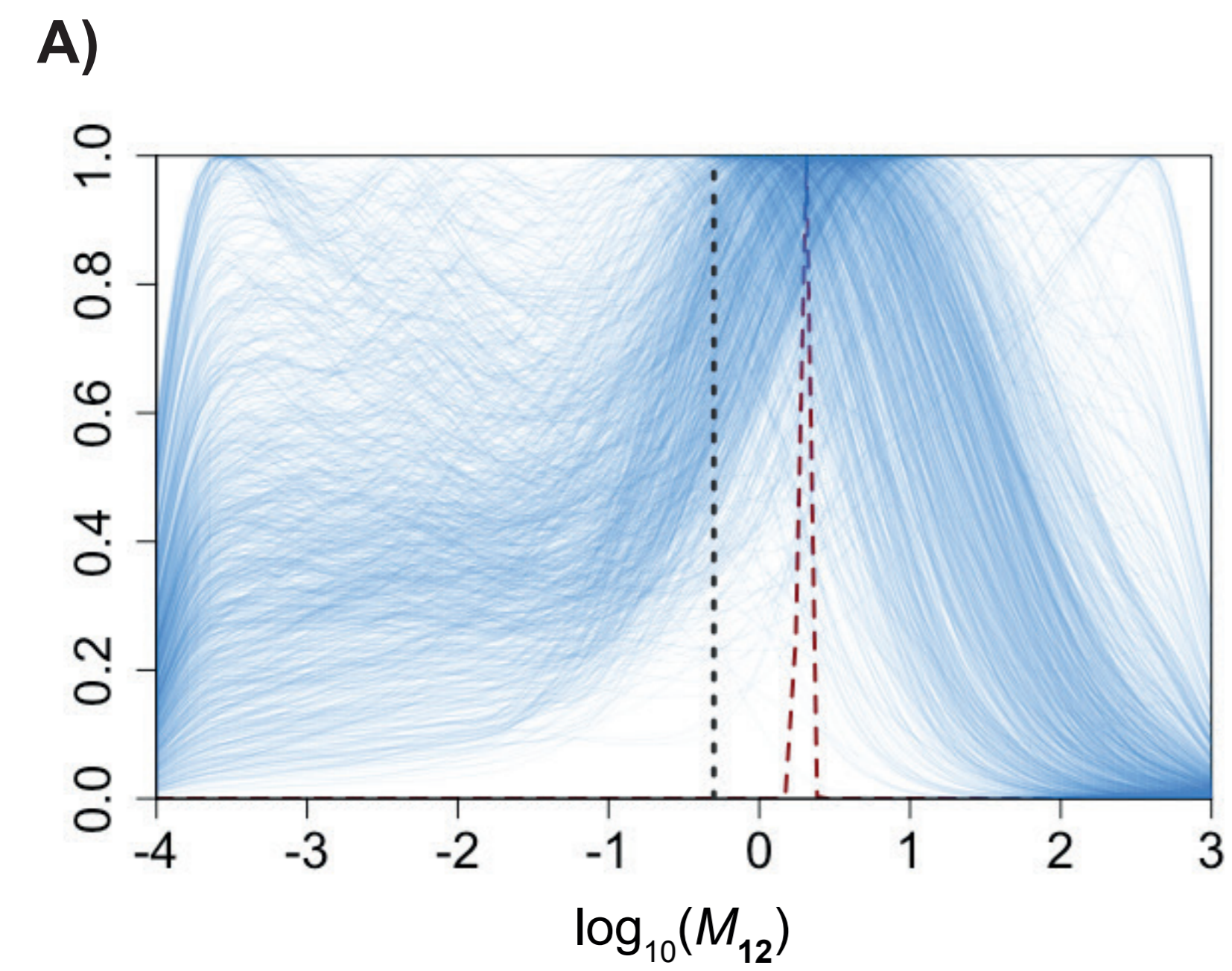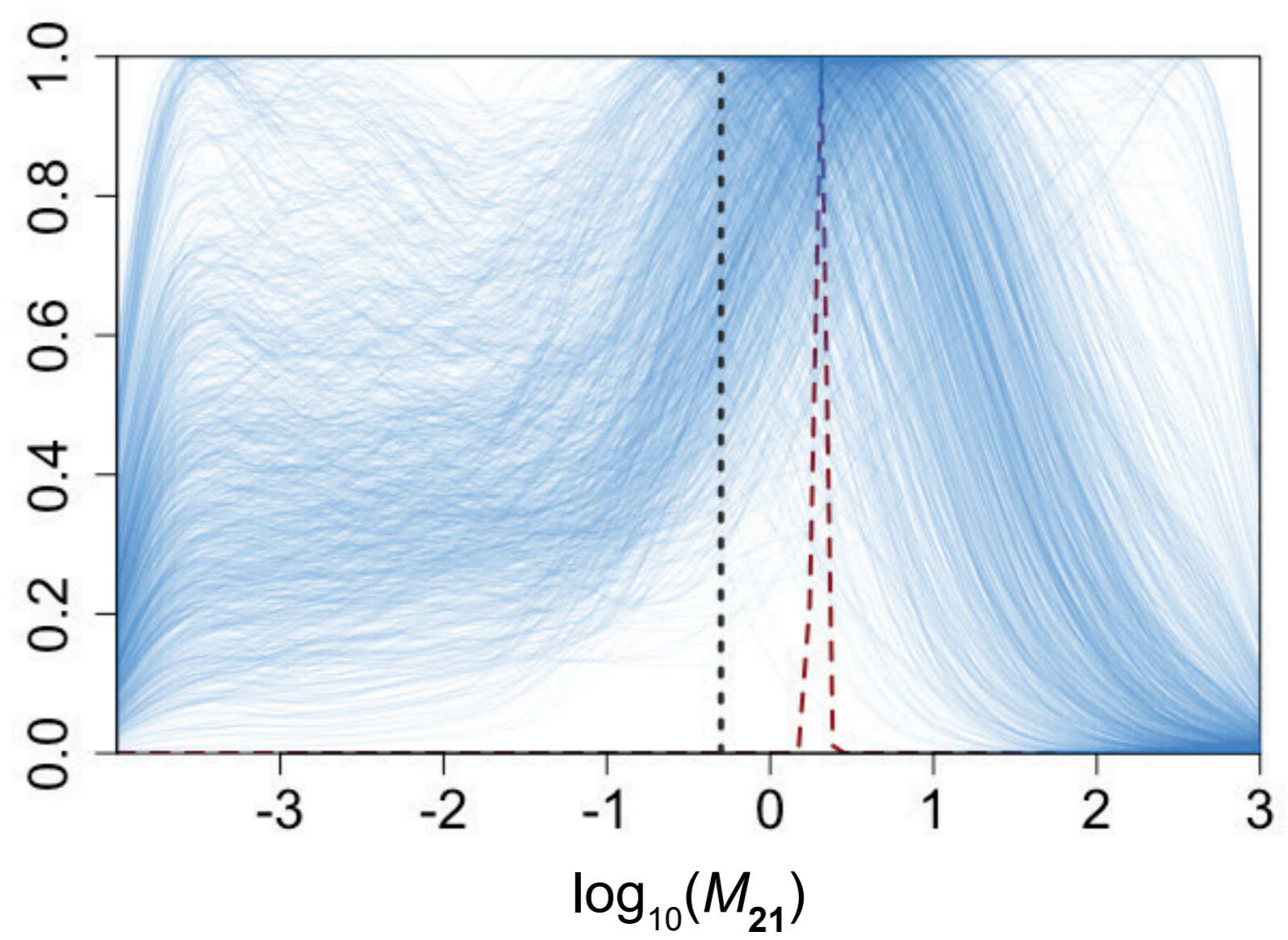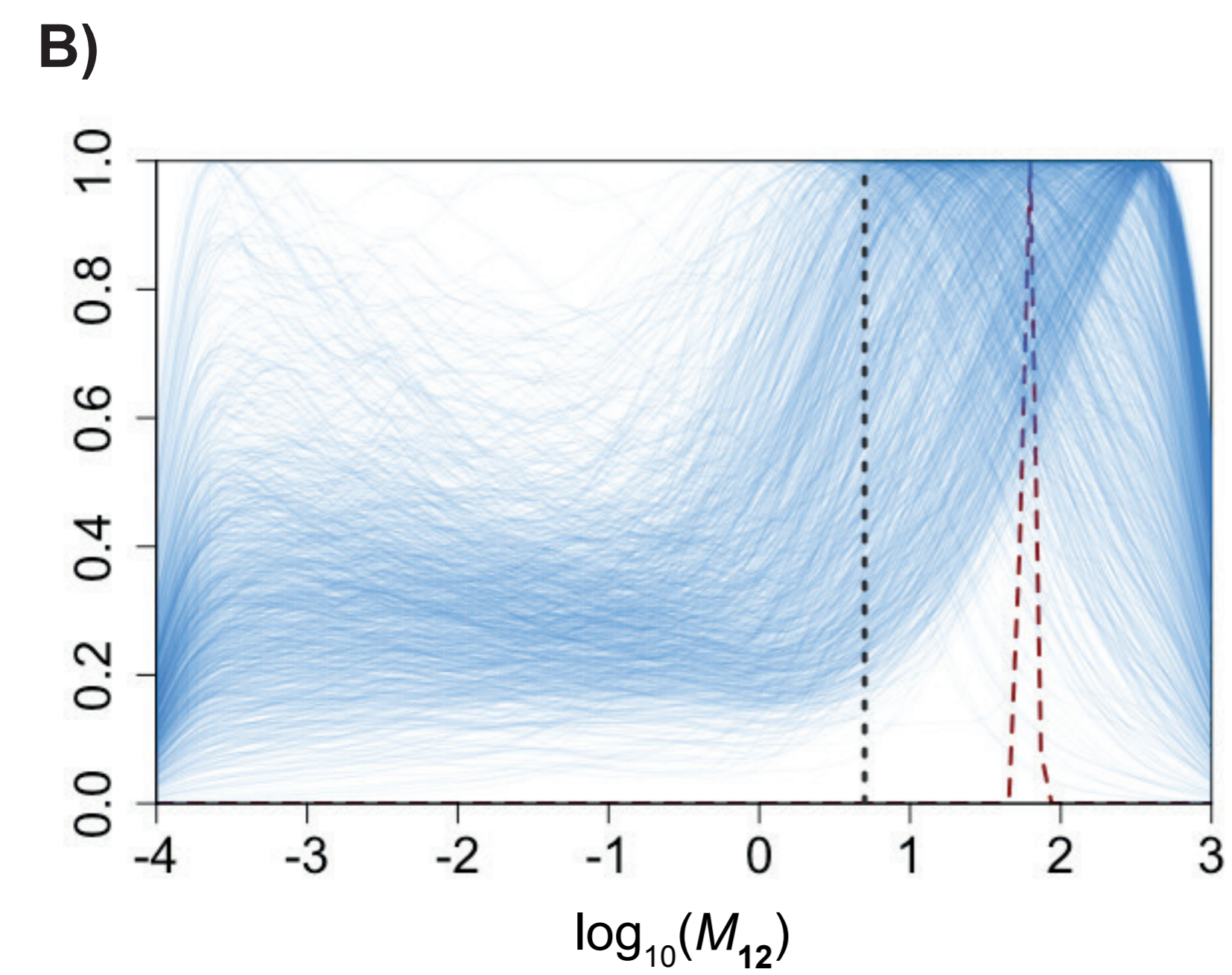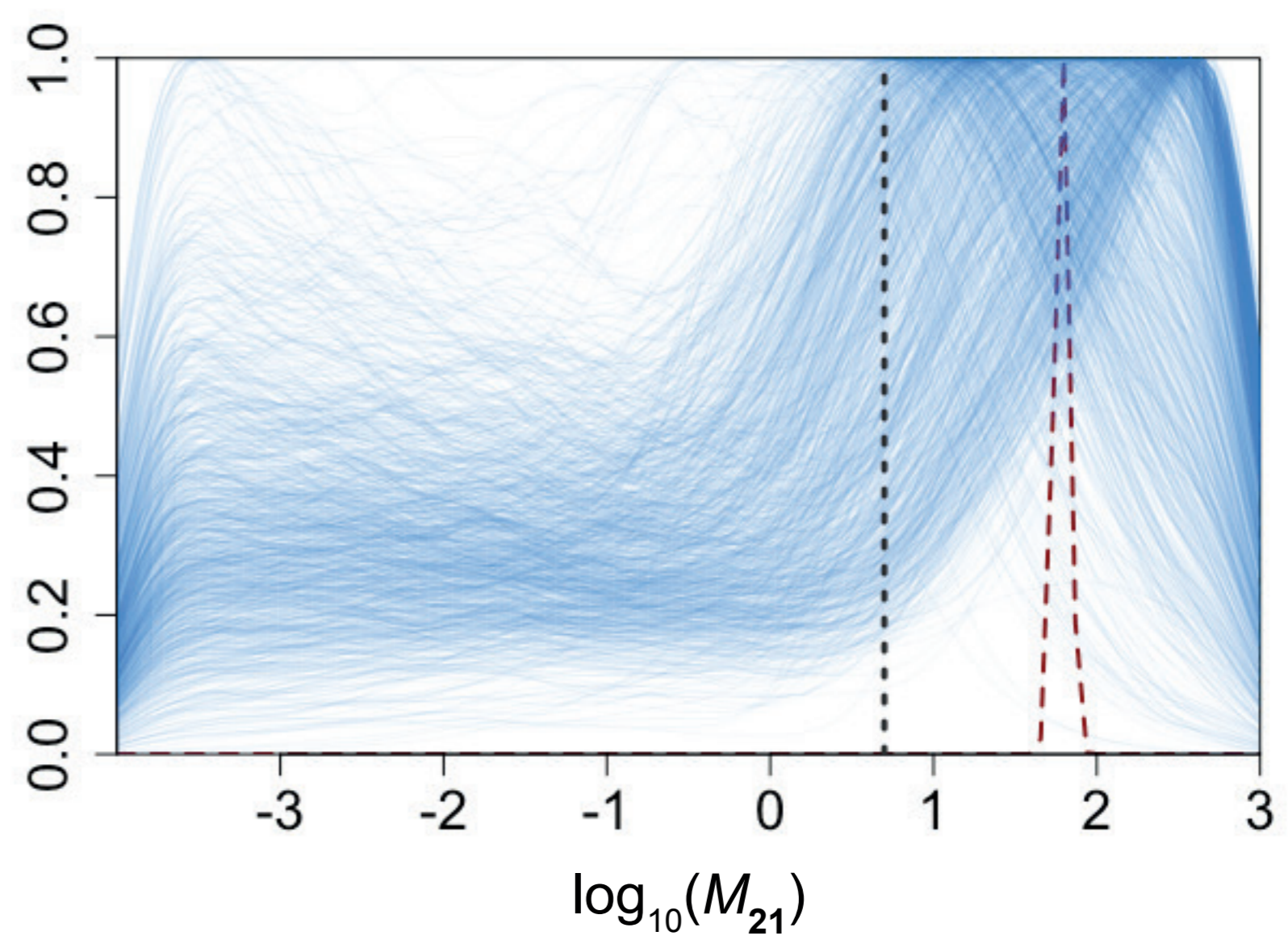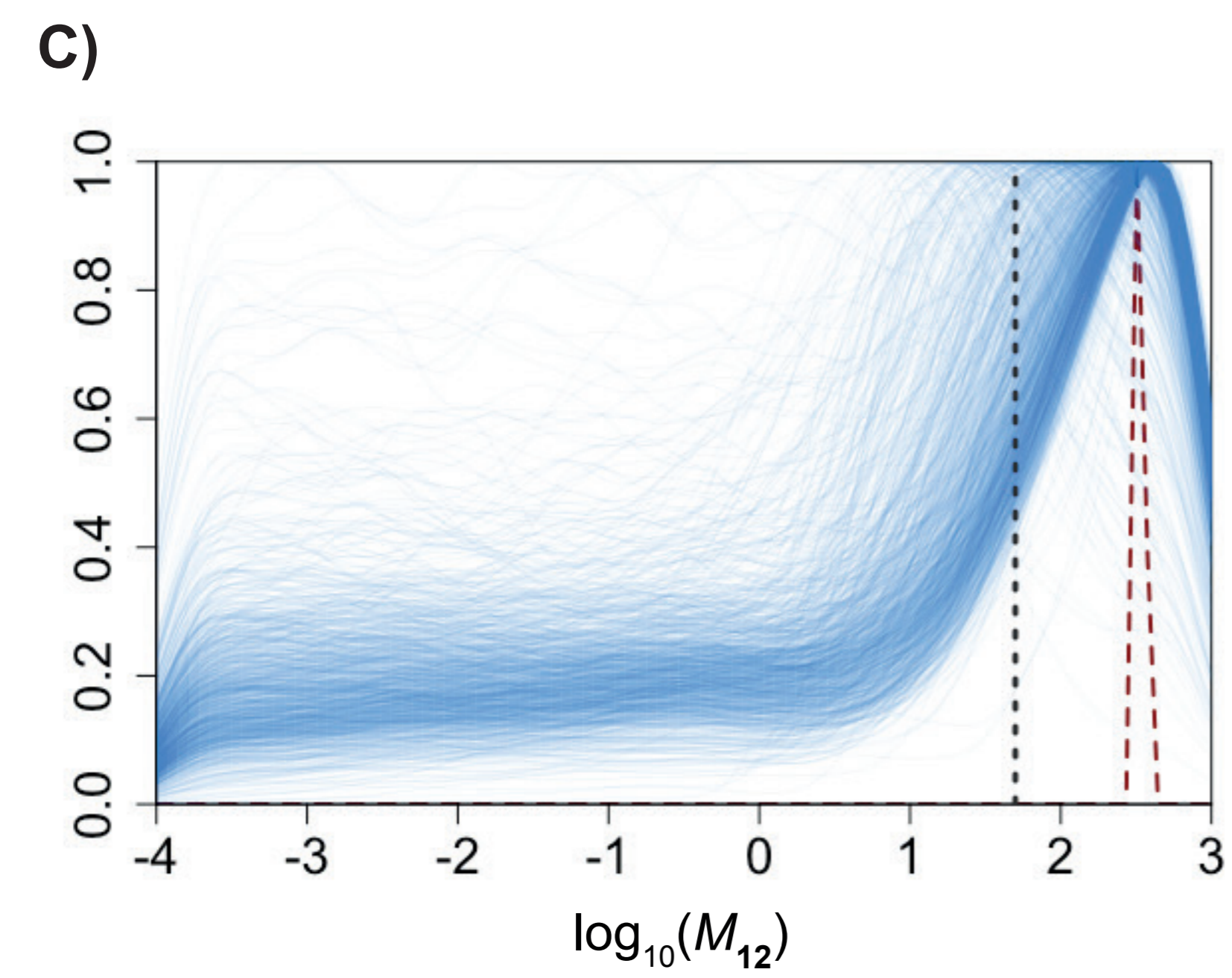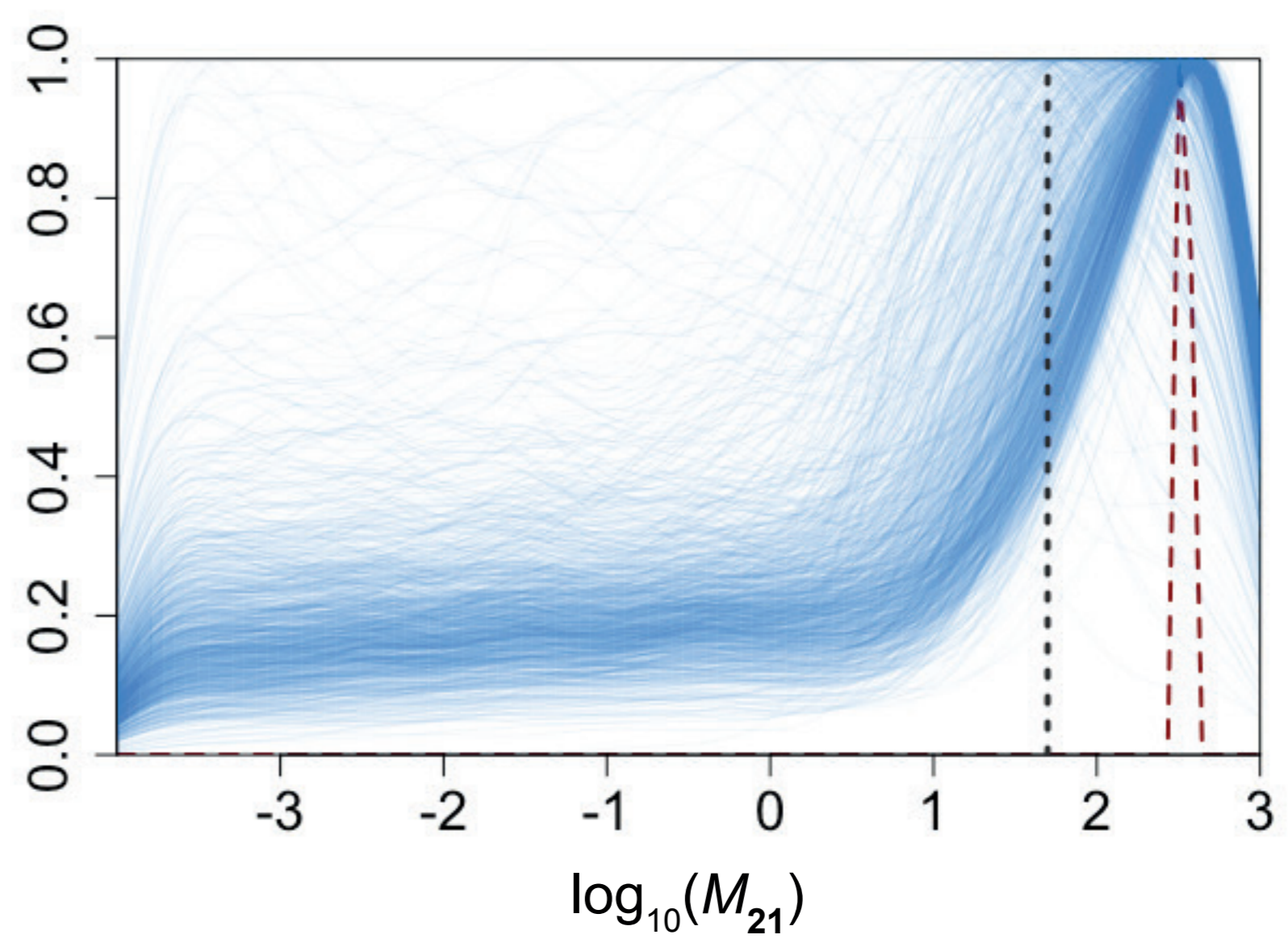

Supplement: Supplementary file 2 — Fig S1‐S16 [file MEN-21-2719-s002.zip › Supplementary Figures/Figure_S11.pdf]

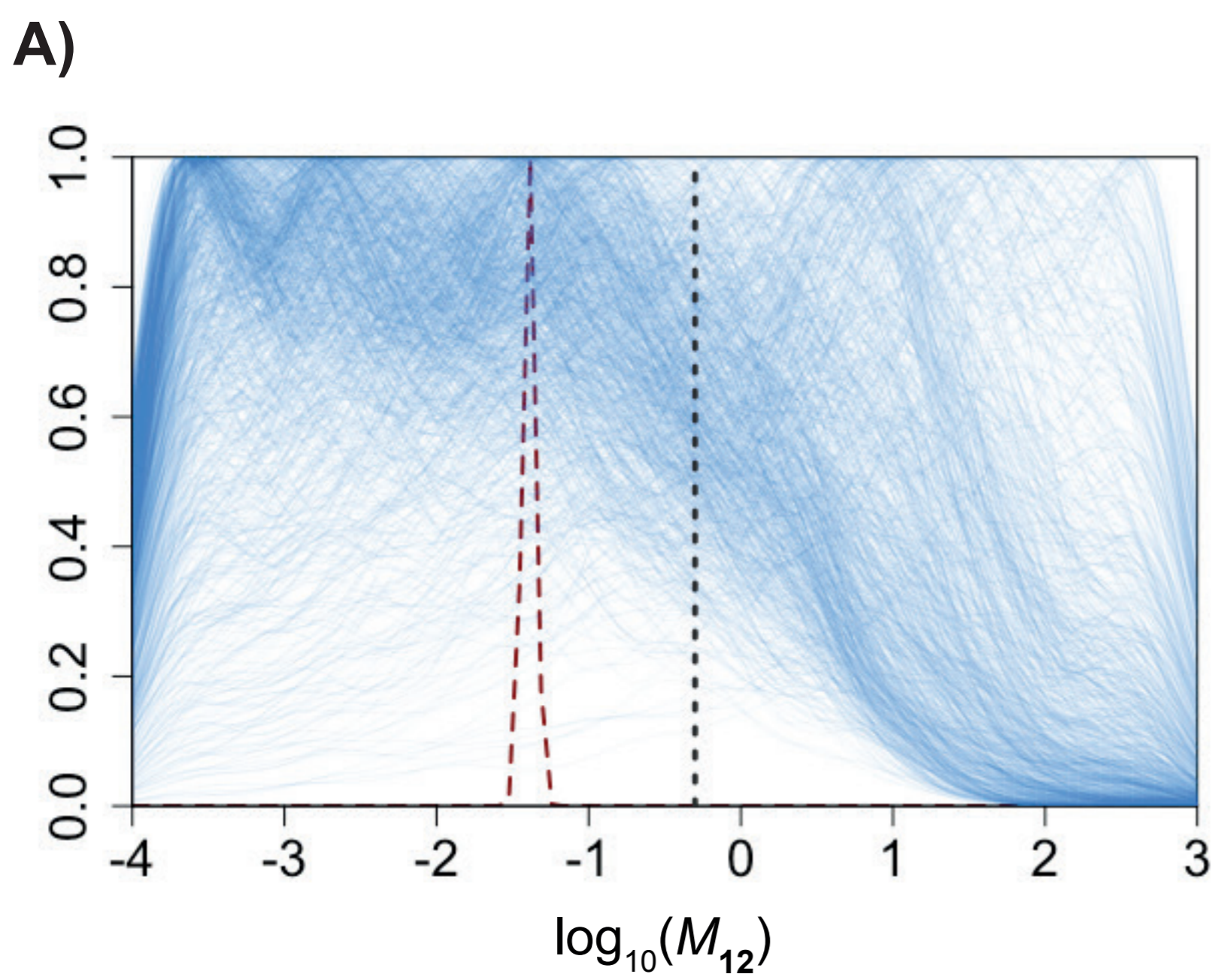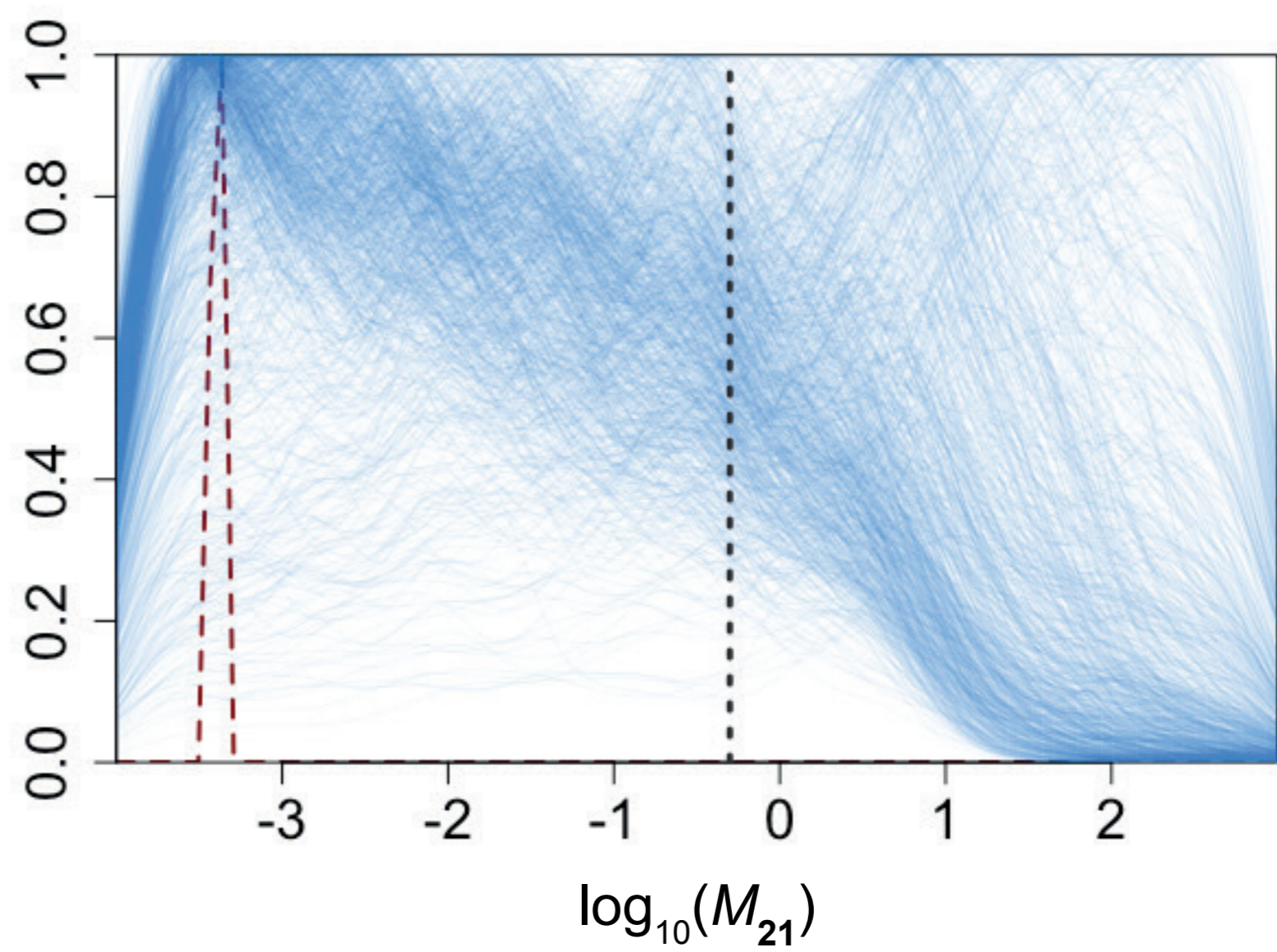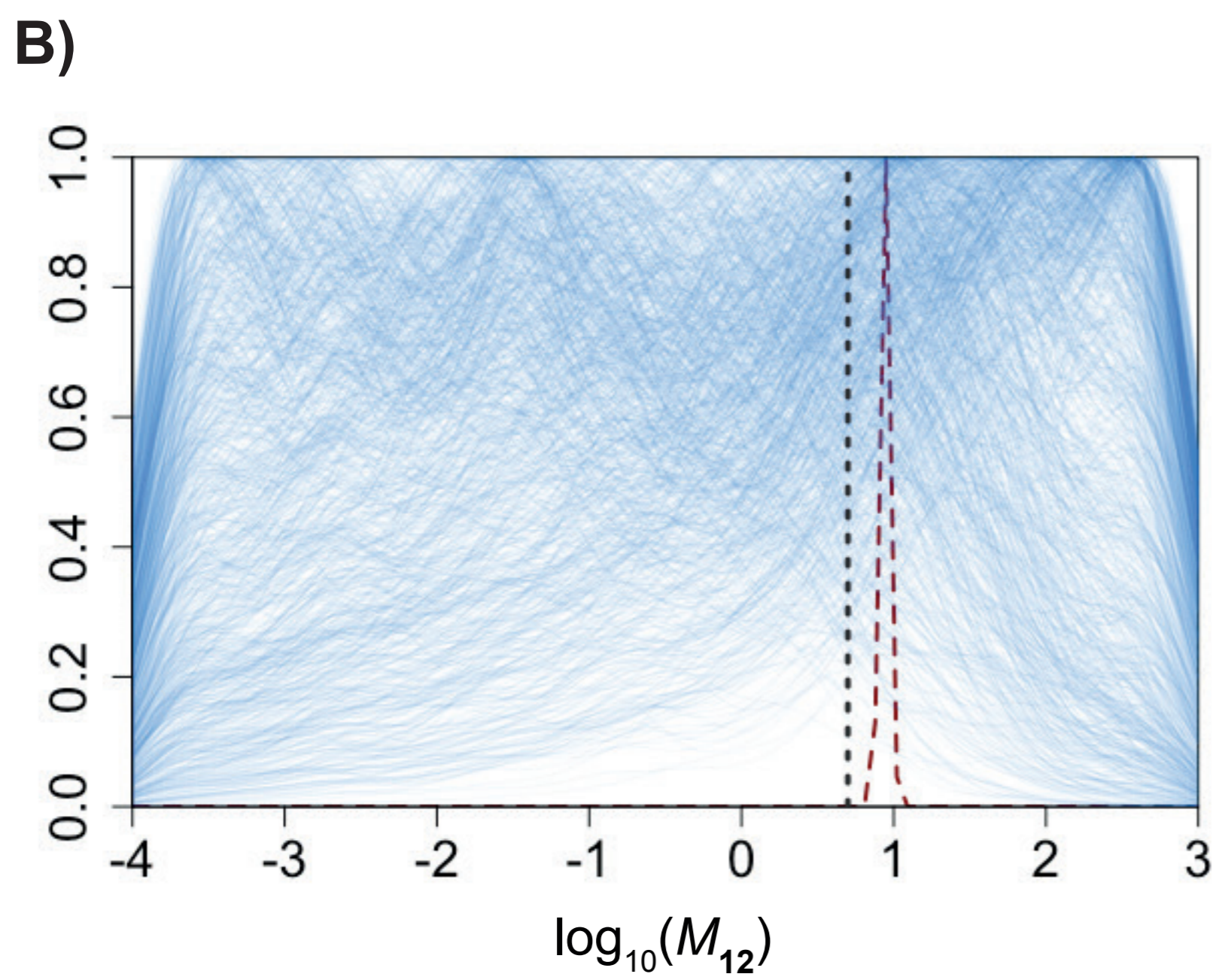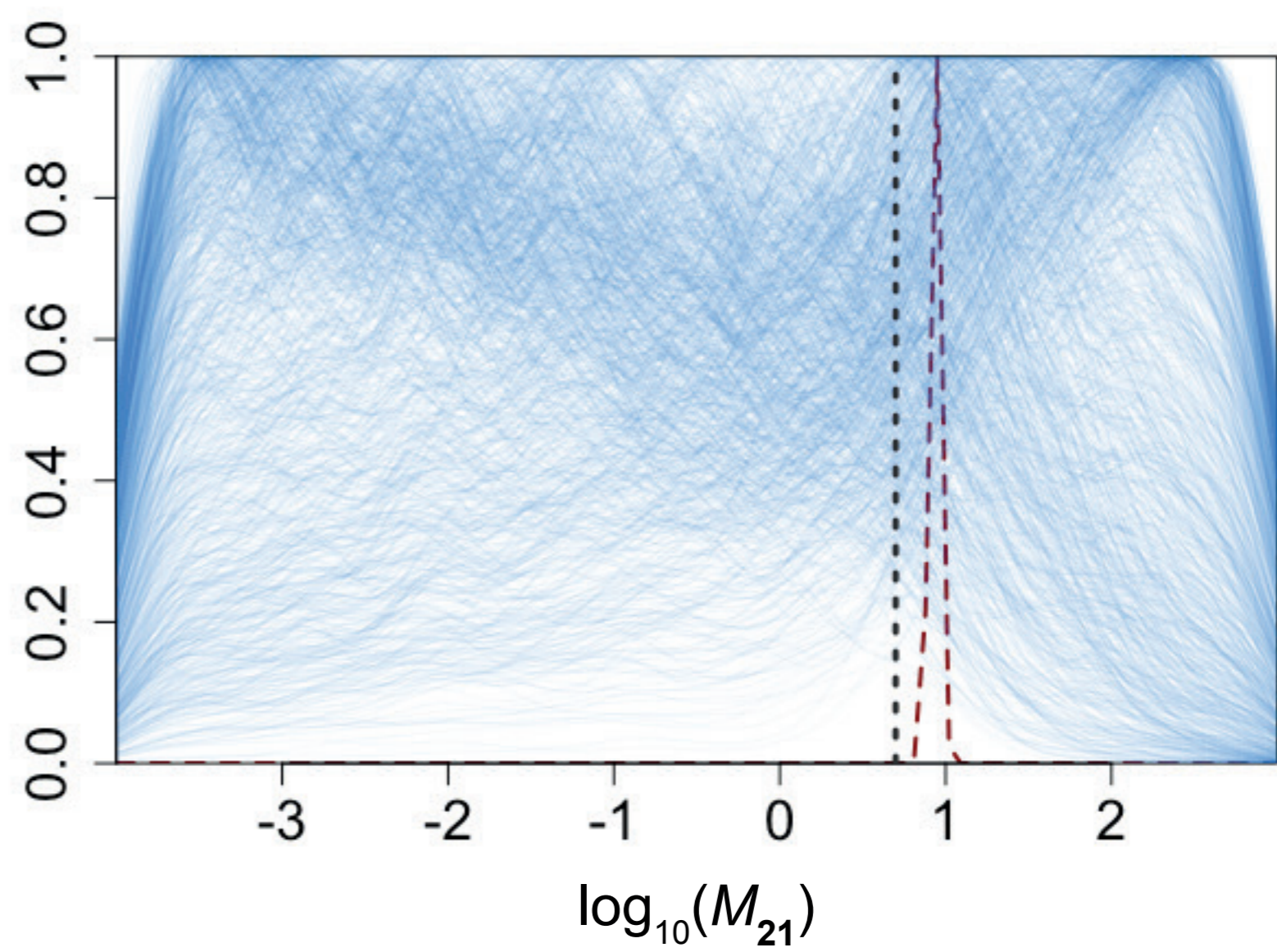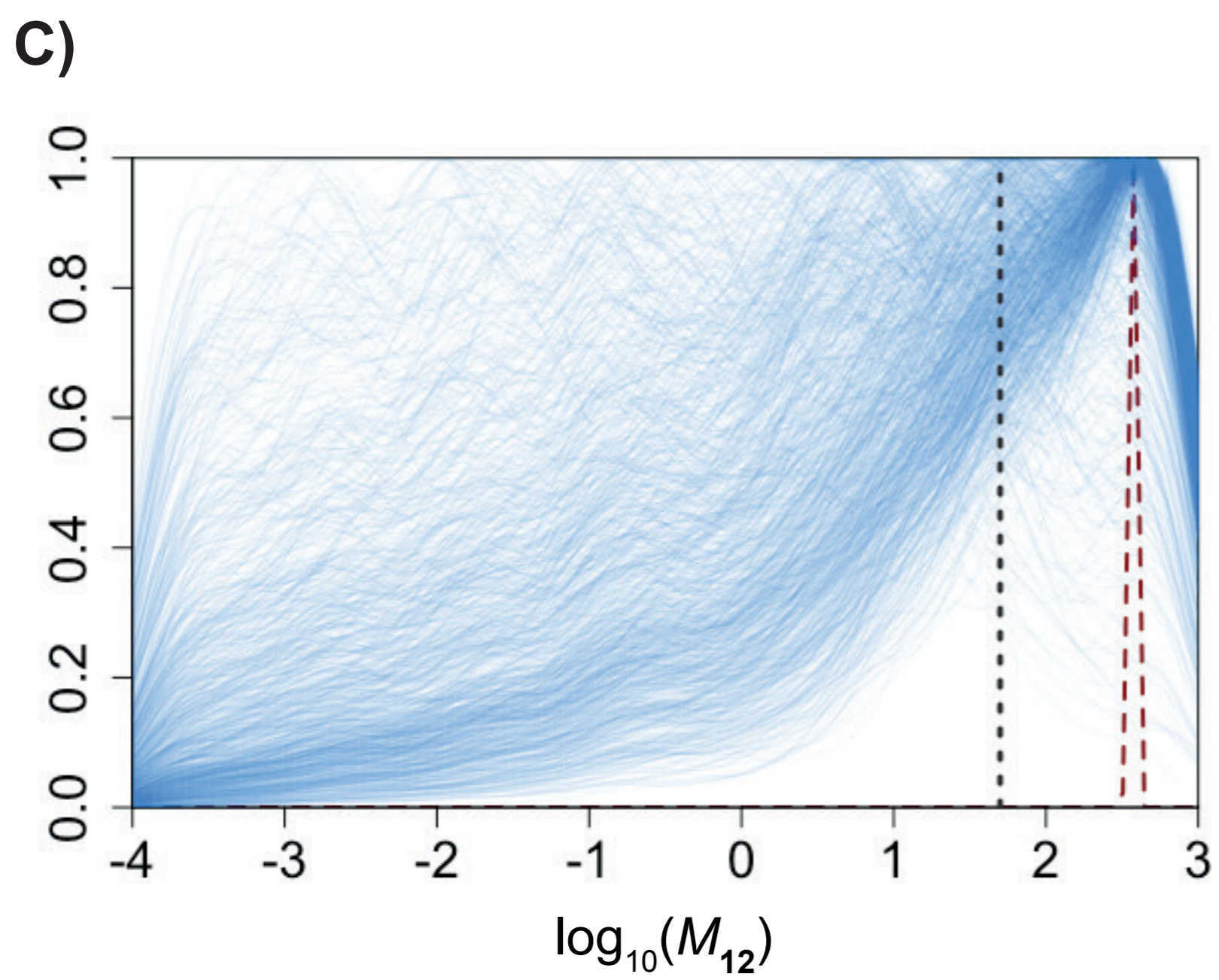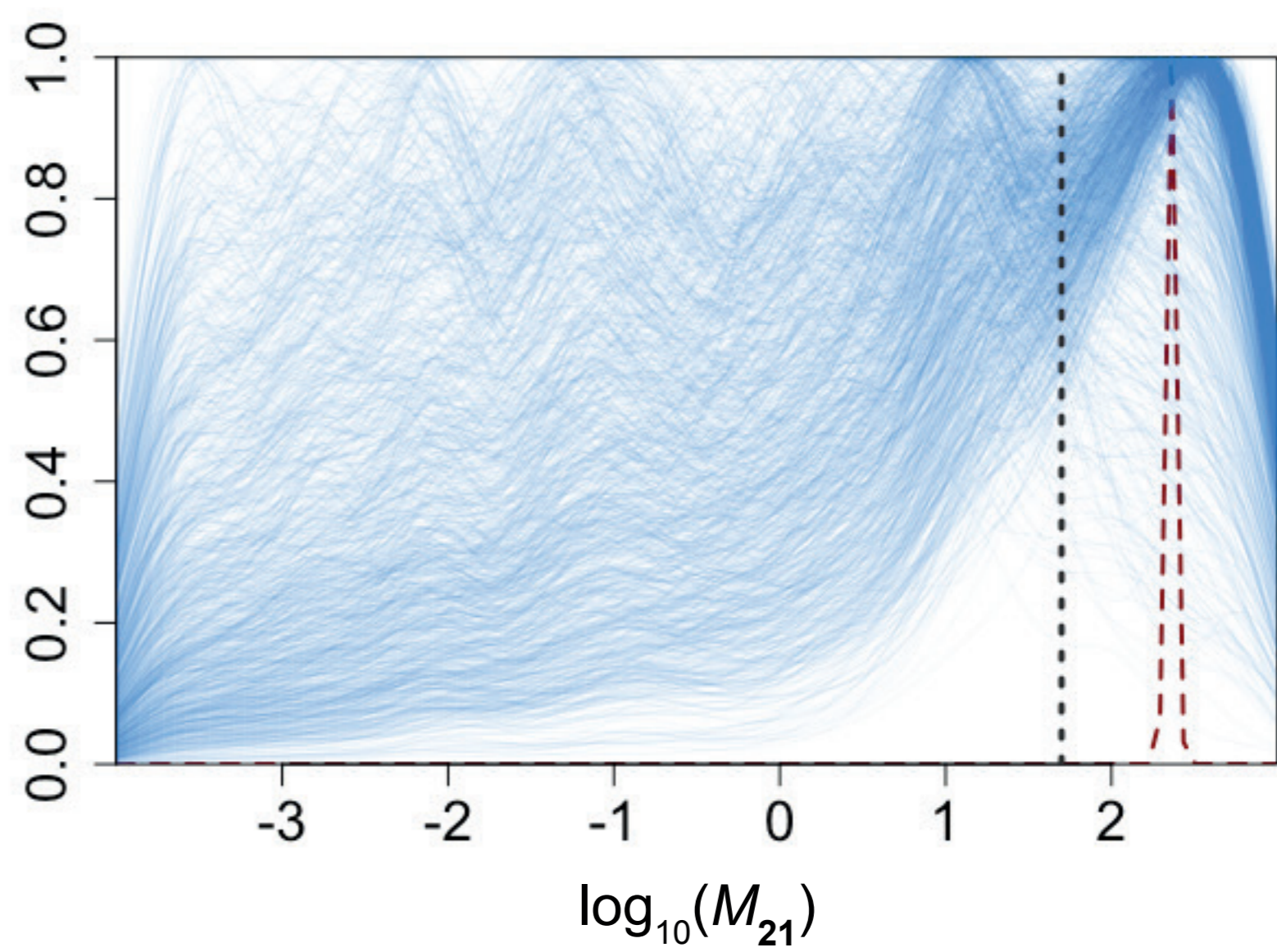

Supplement: Supplementary file 2 — Fig S1‐S16 [file MEN-21-2719-s002.zip › Supplementary Figures/Figure_S12.pdf]

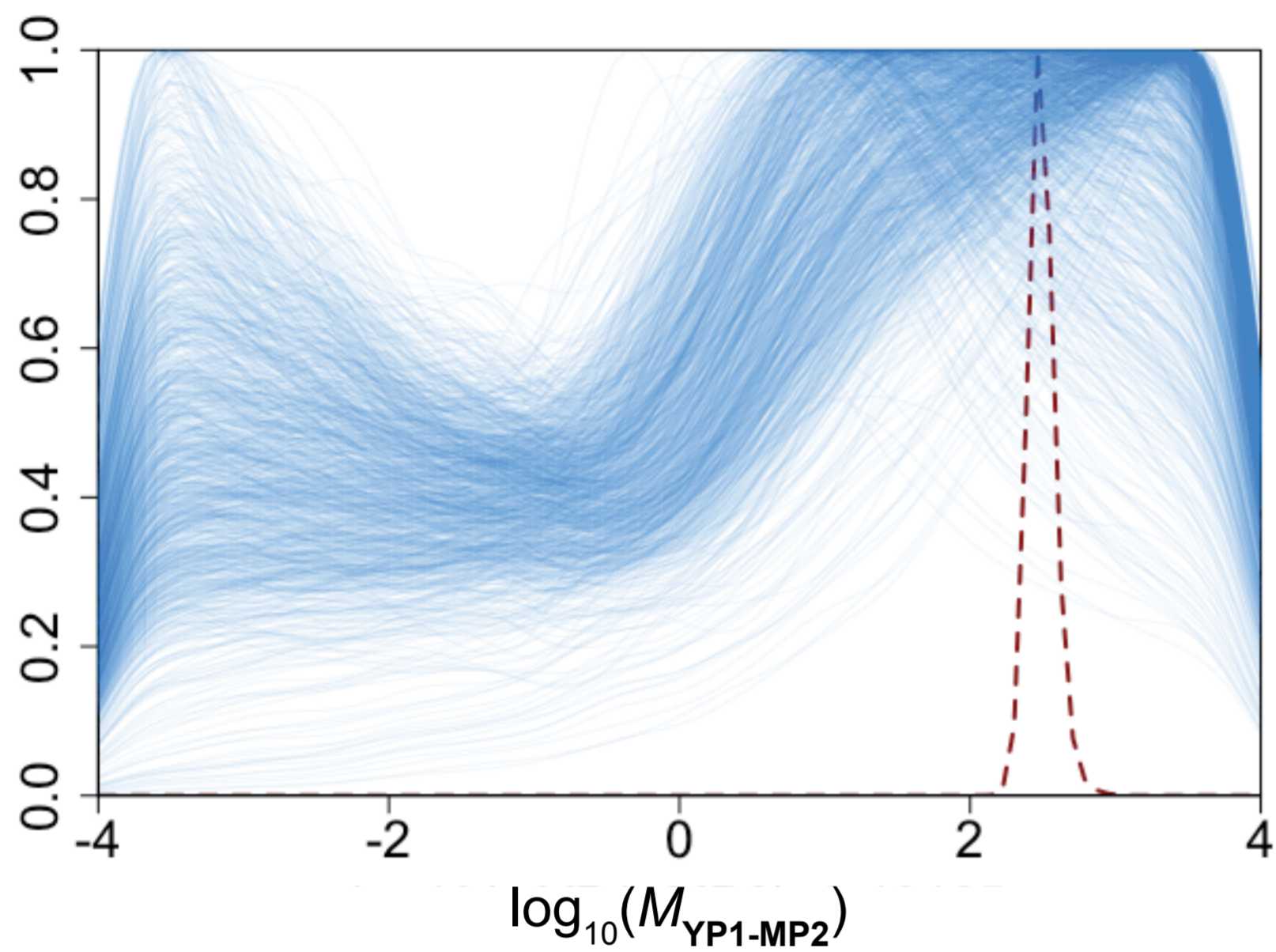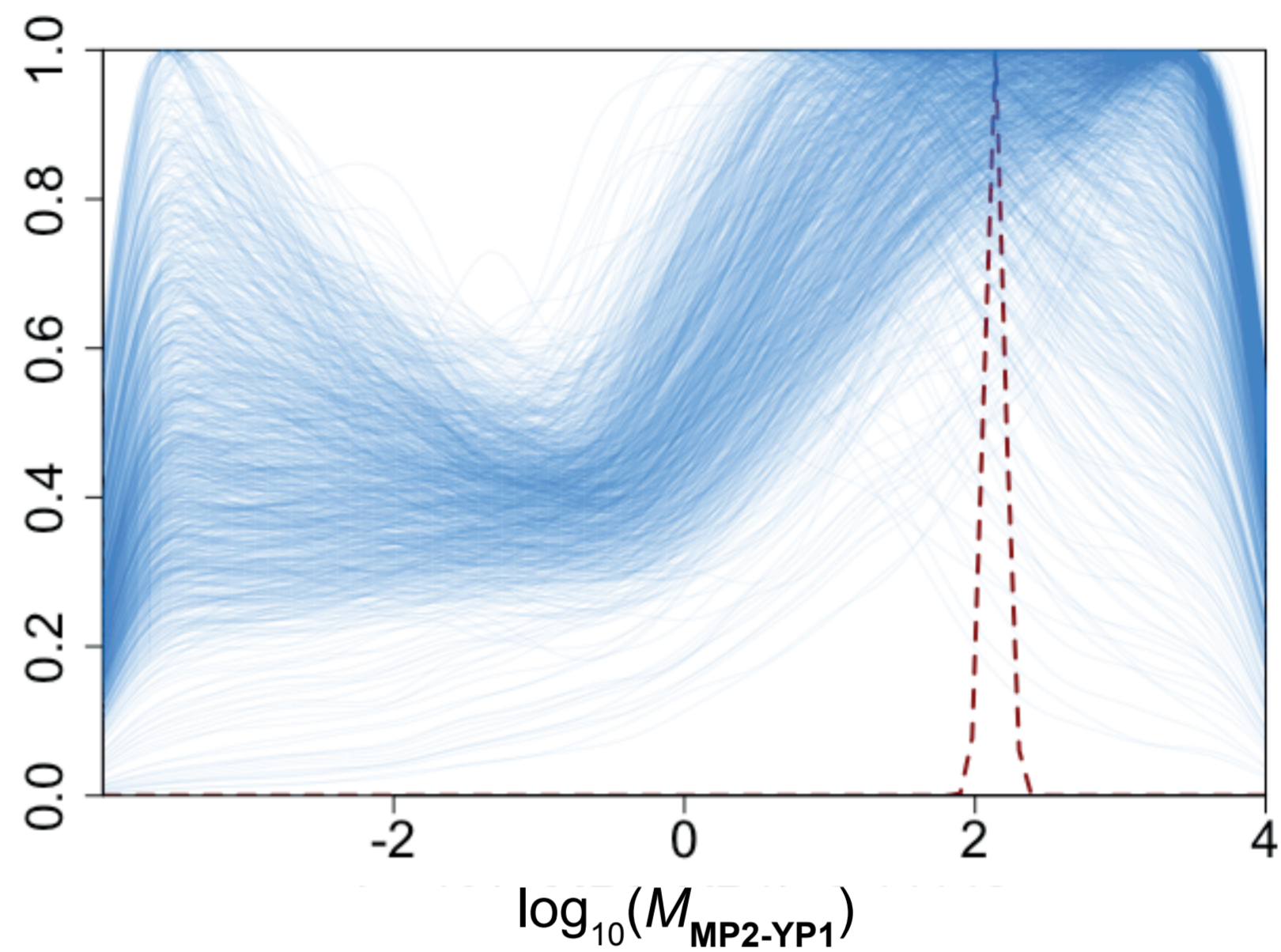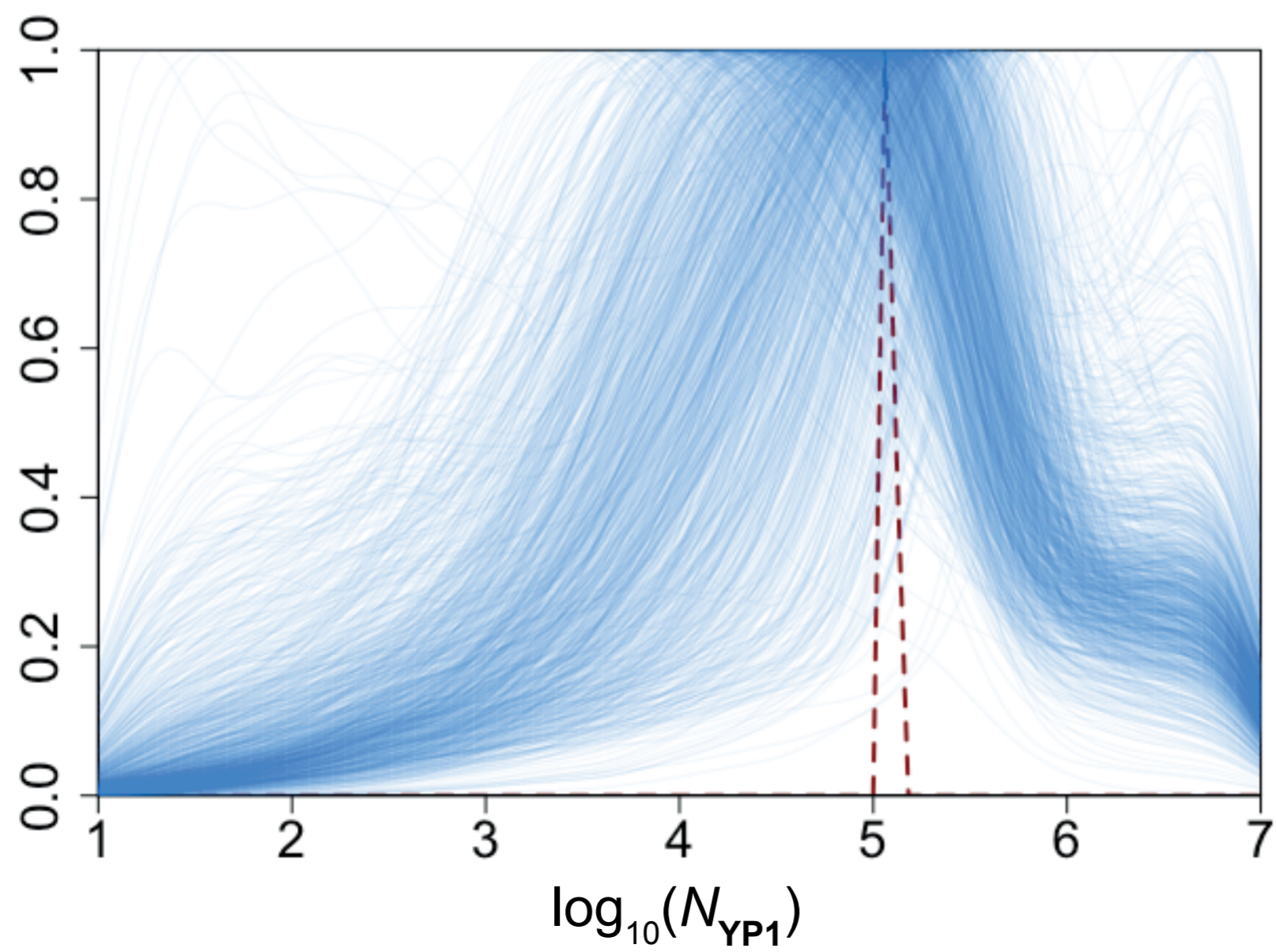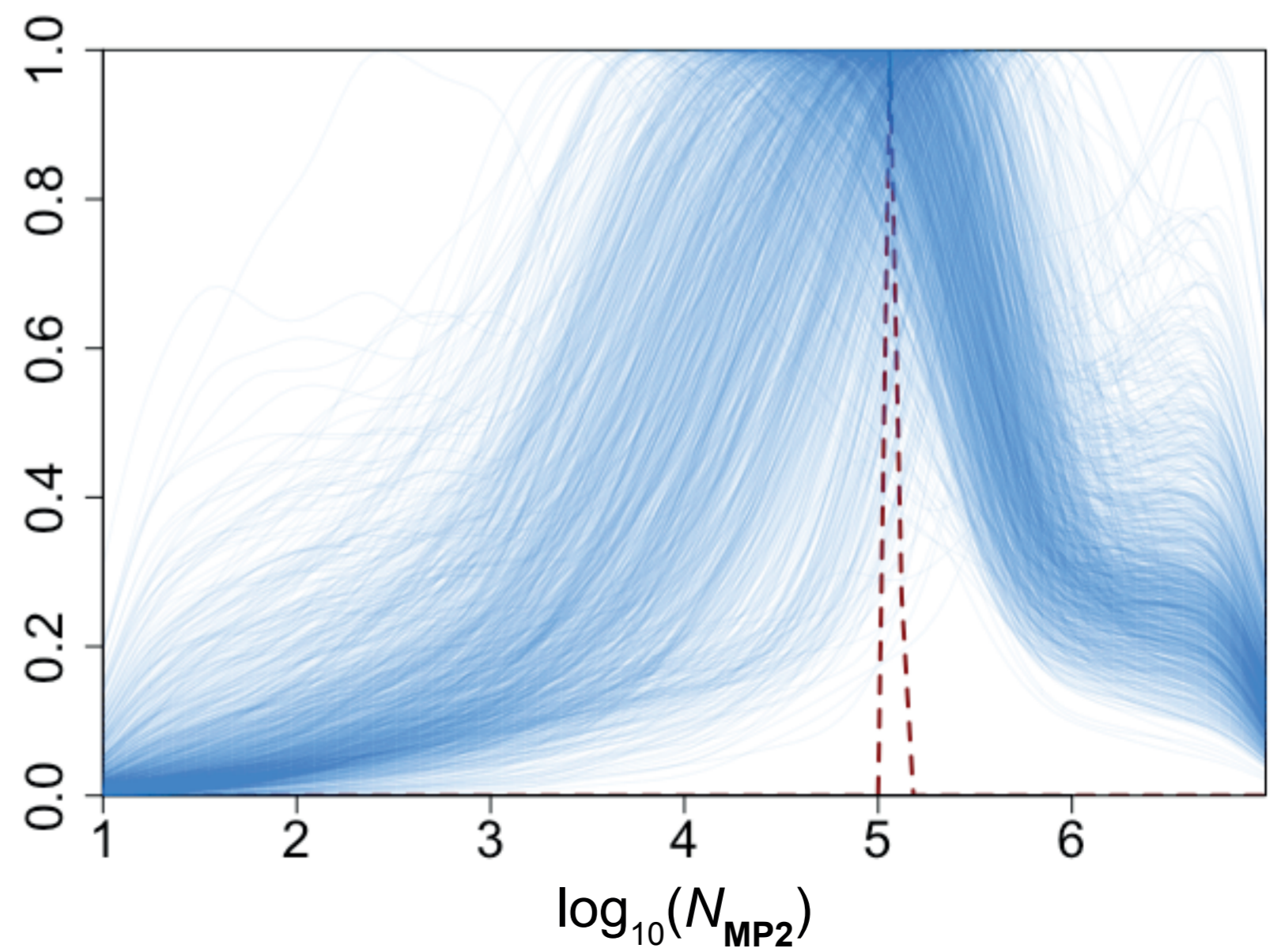

Supplement: Supplementary file 2 — Fig S1‐S16 [file MEN-21-2719-s002.zip › Supplementary Figures/Figure_S13.pdf]

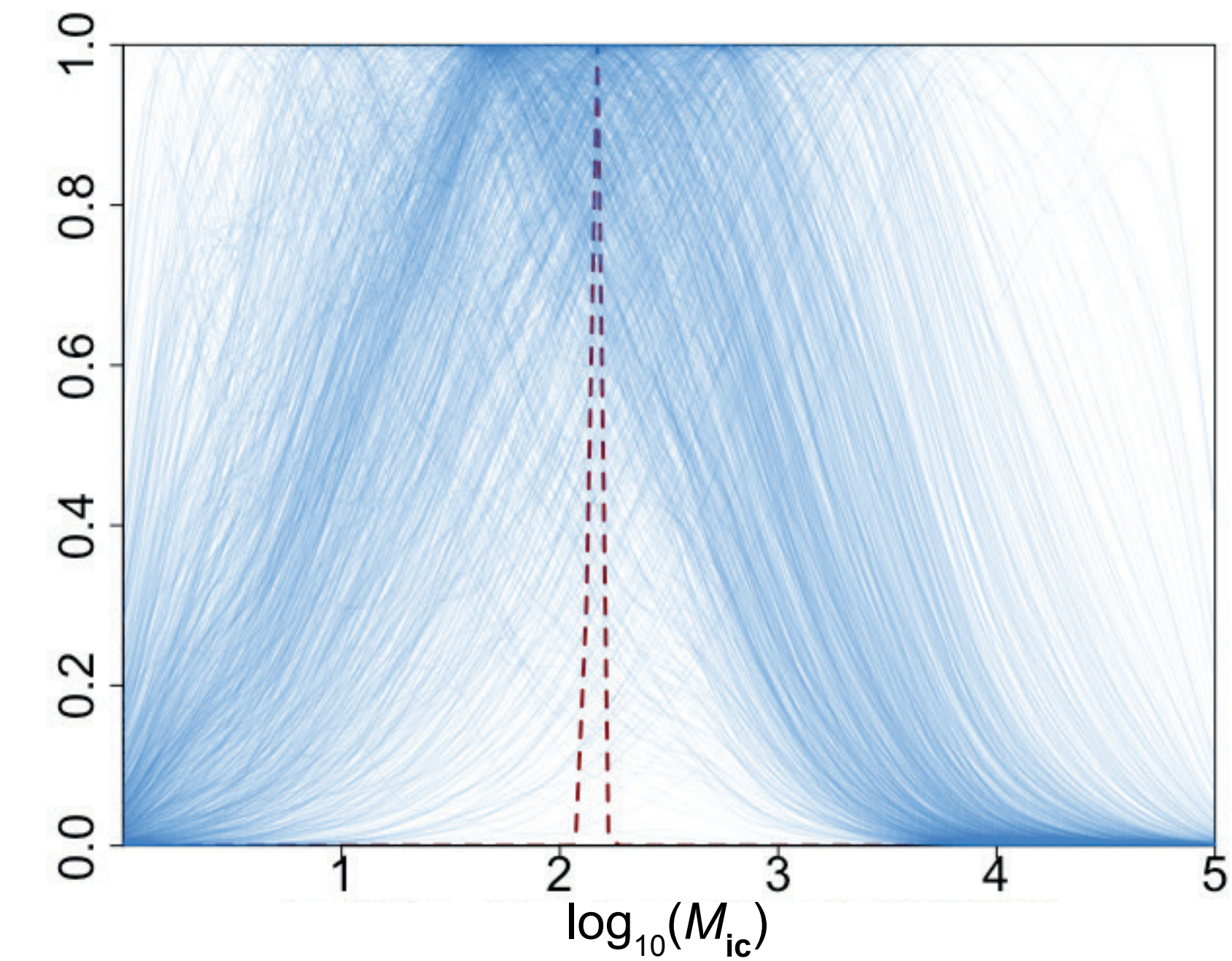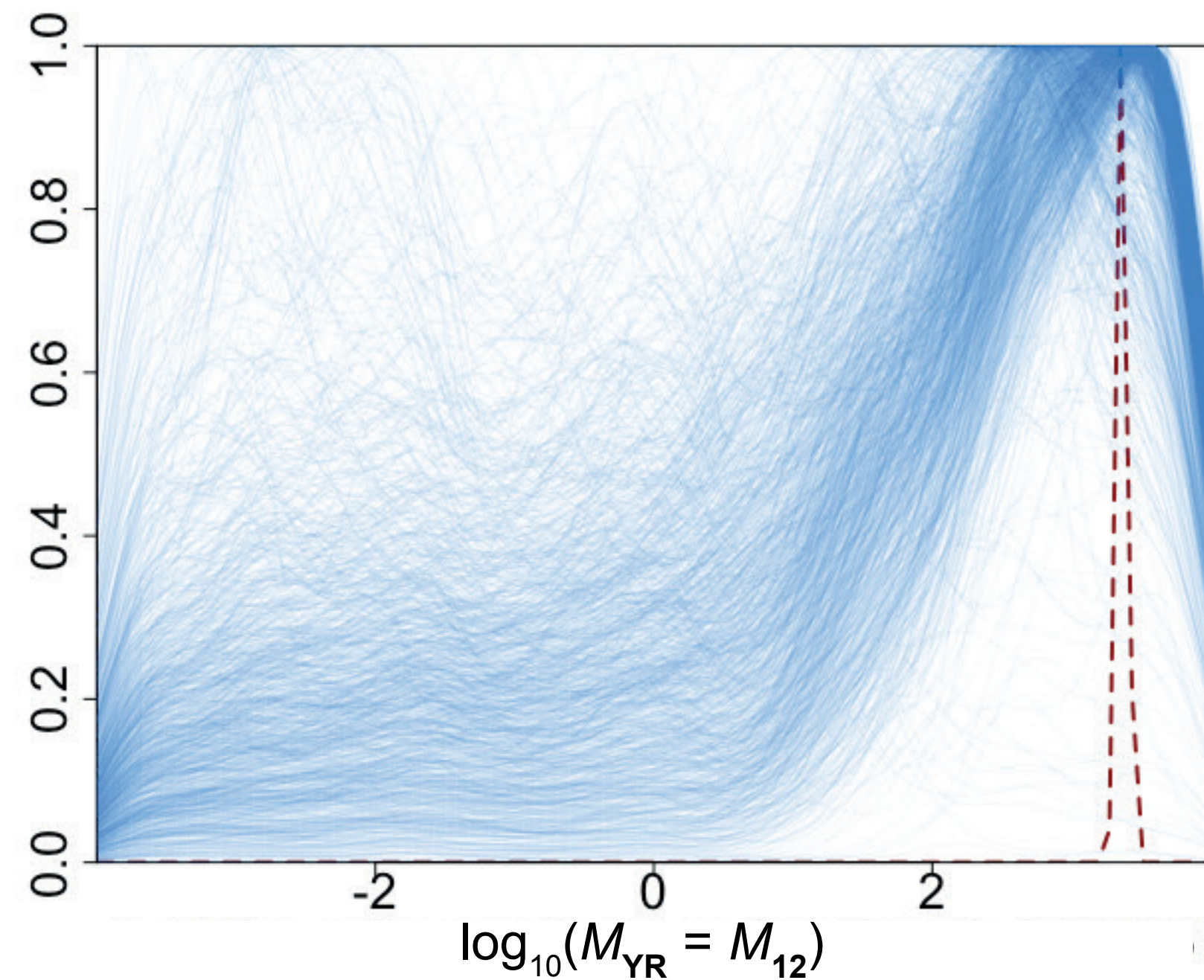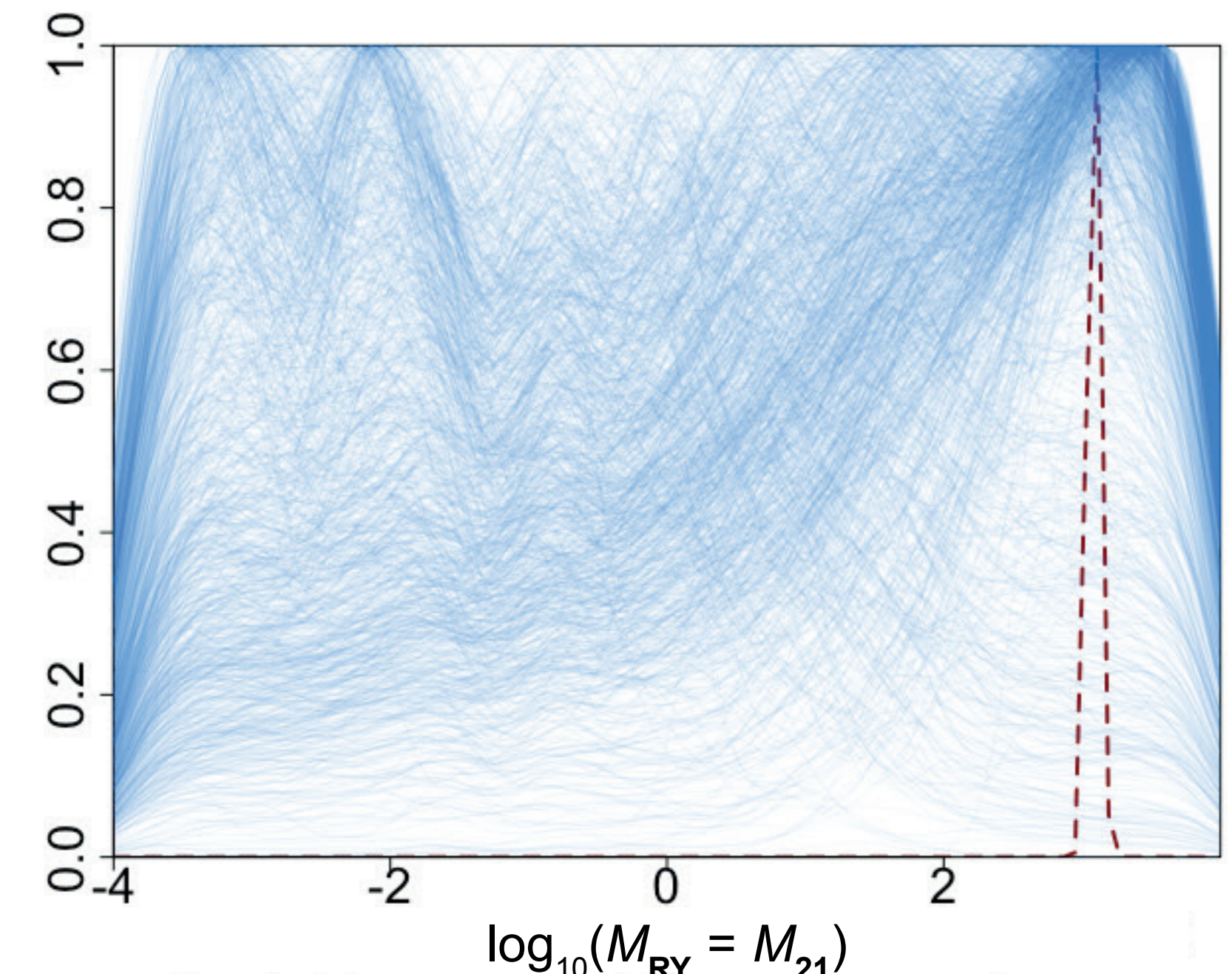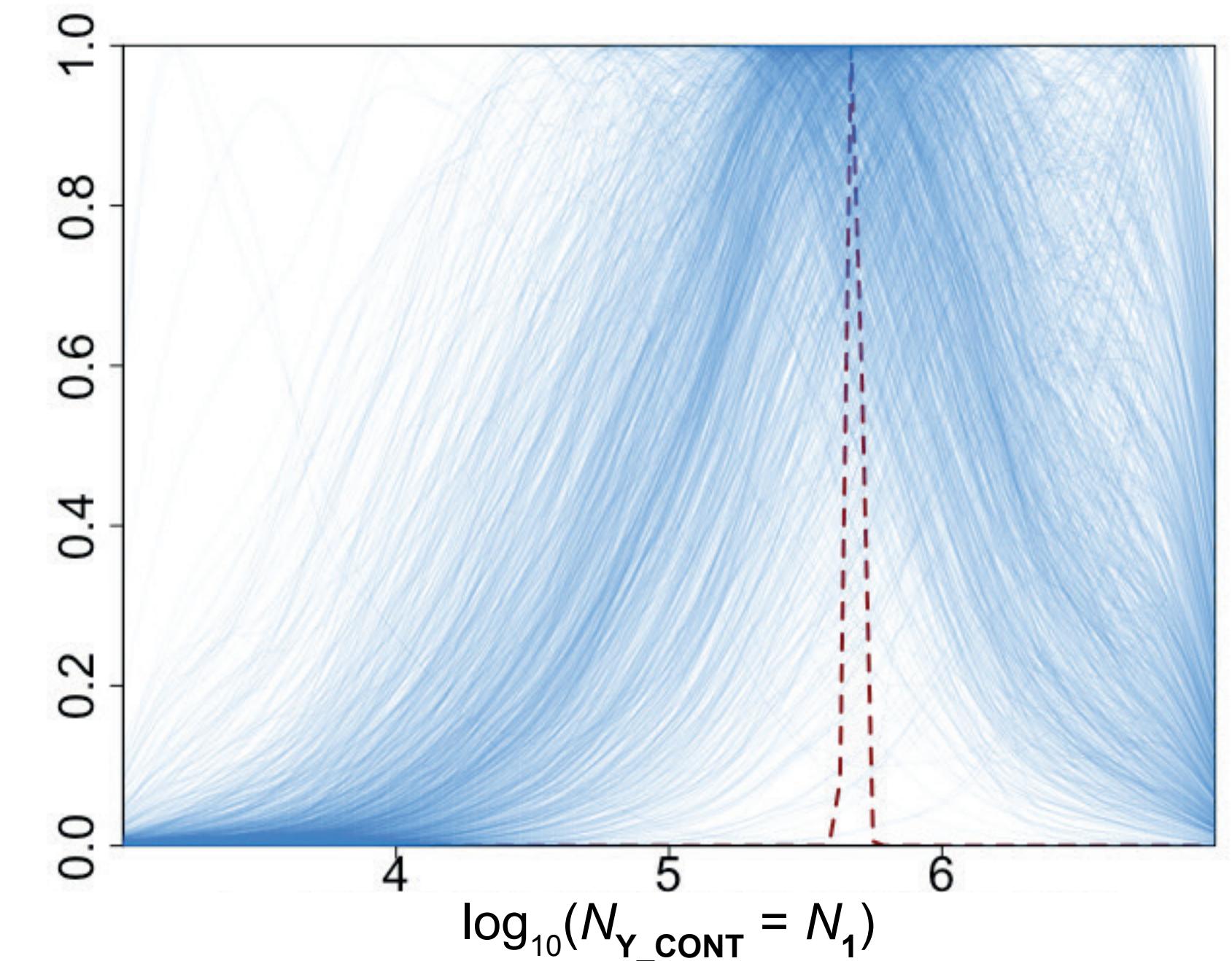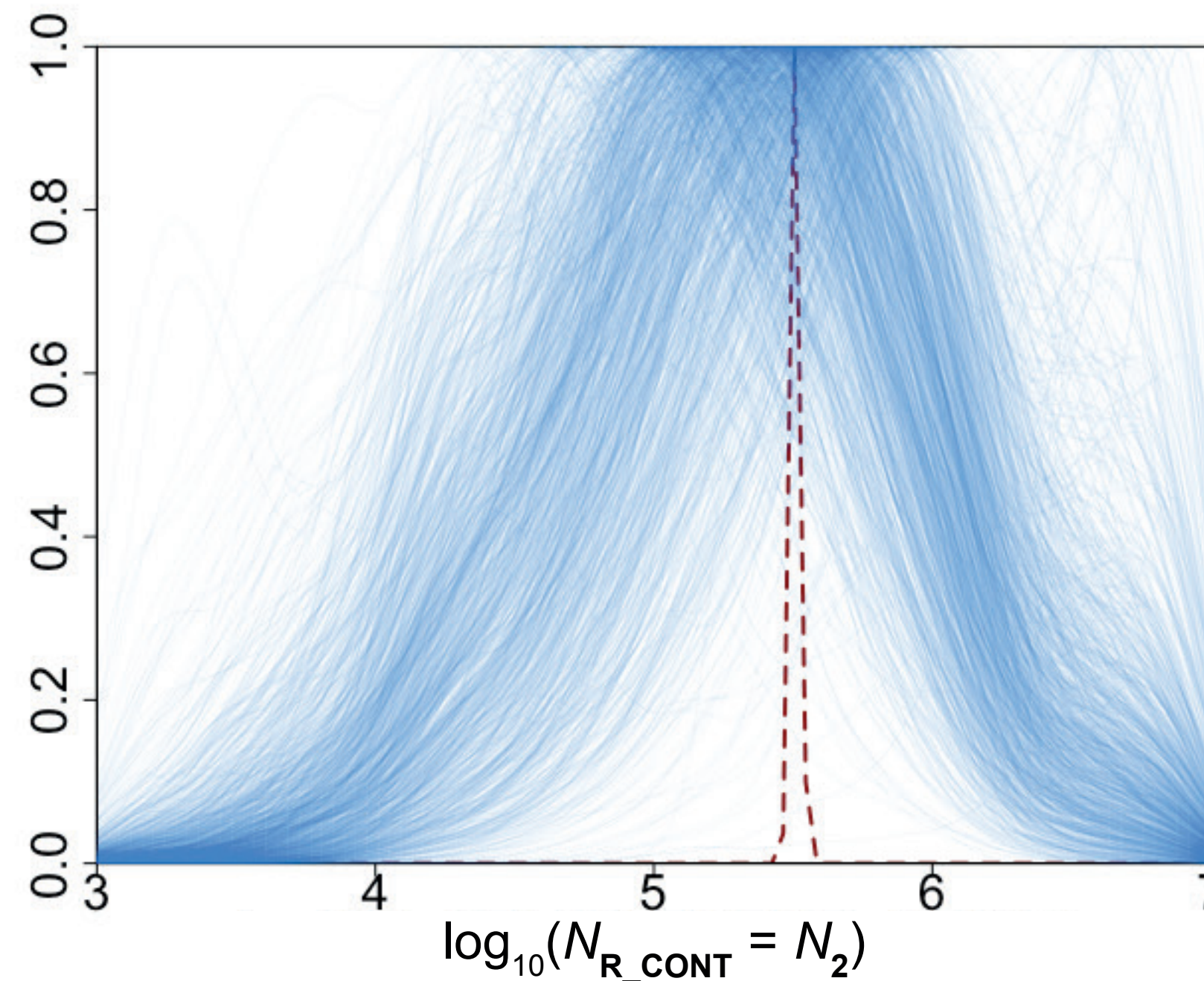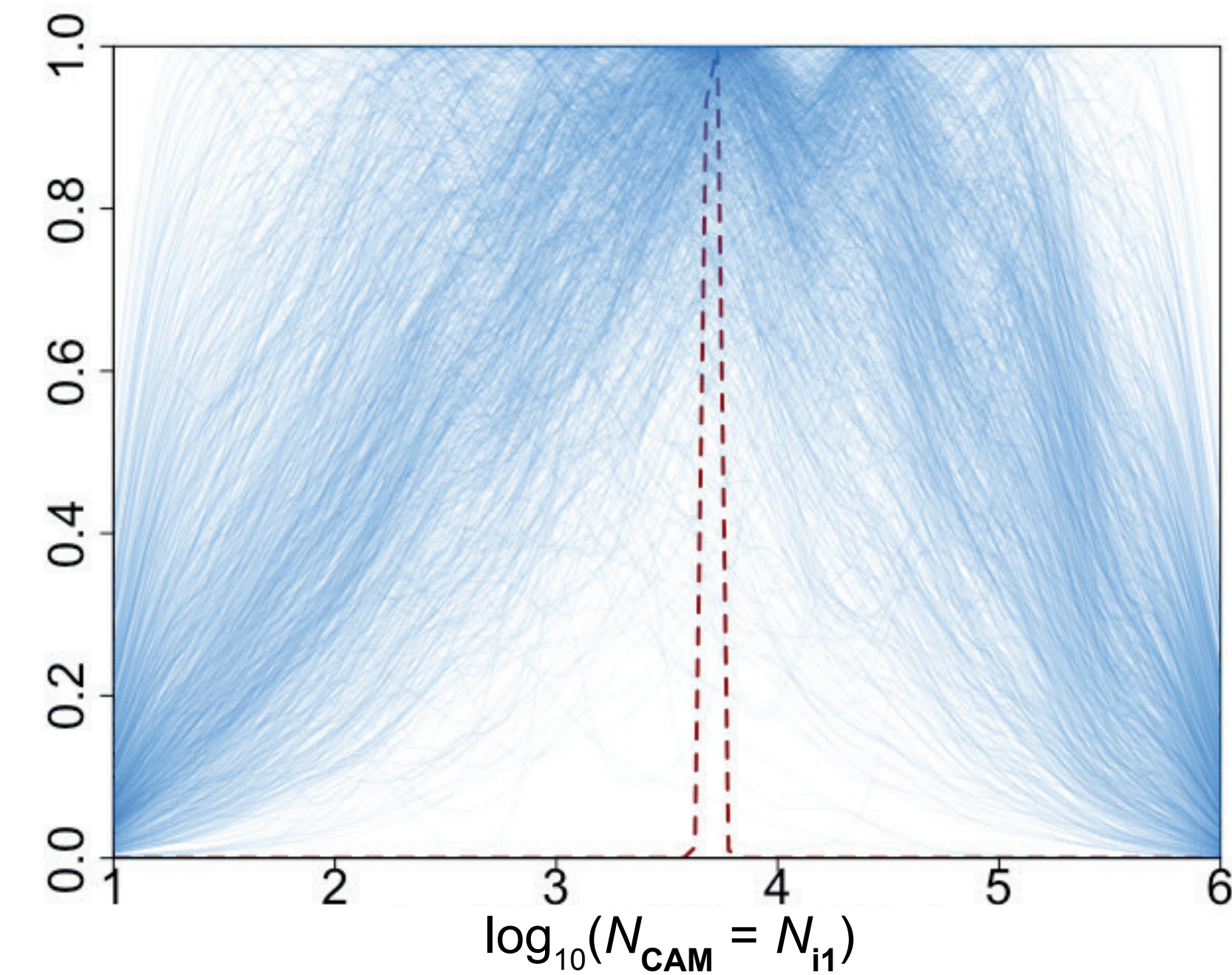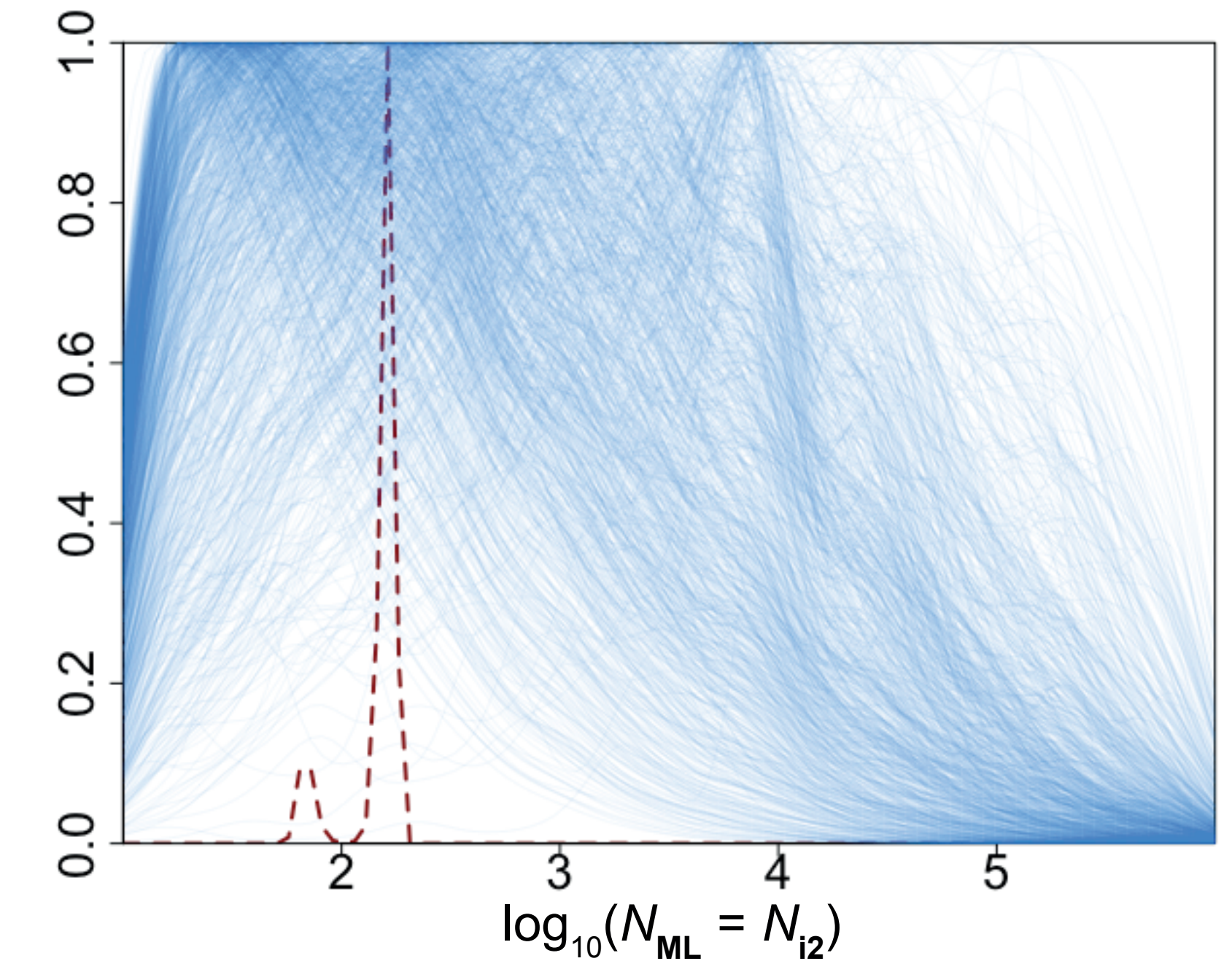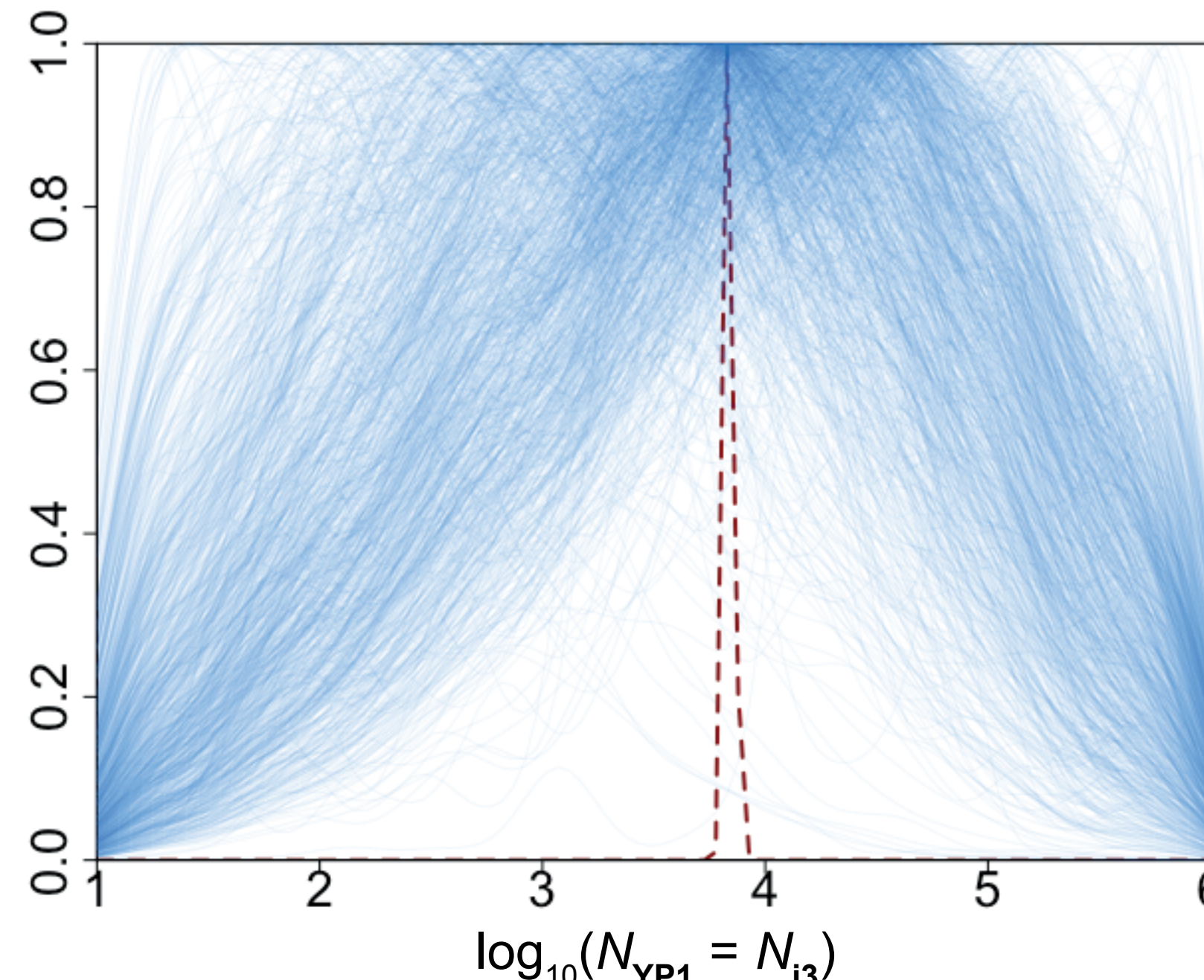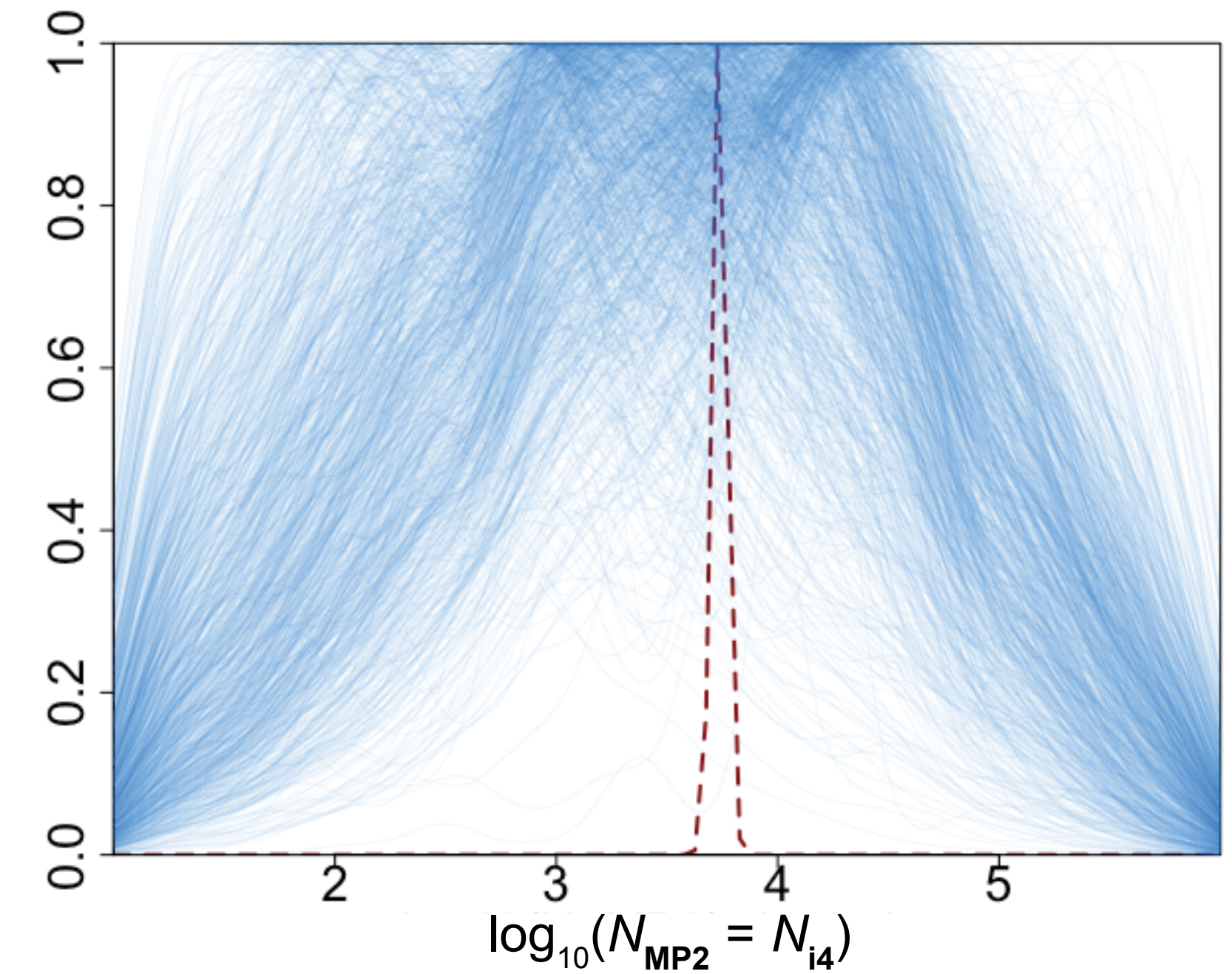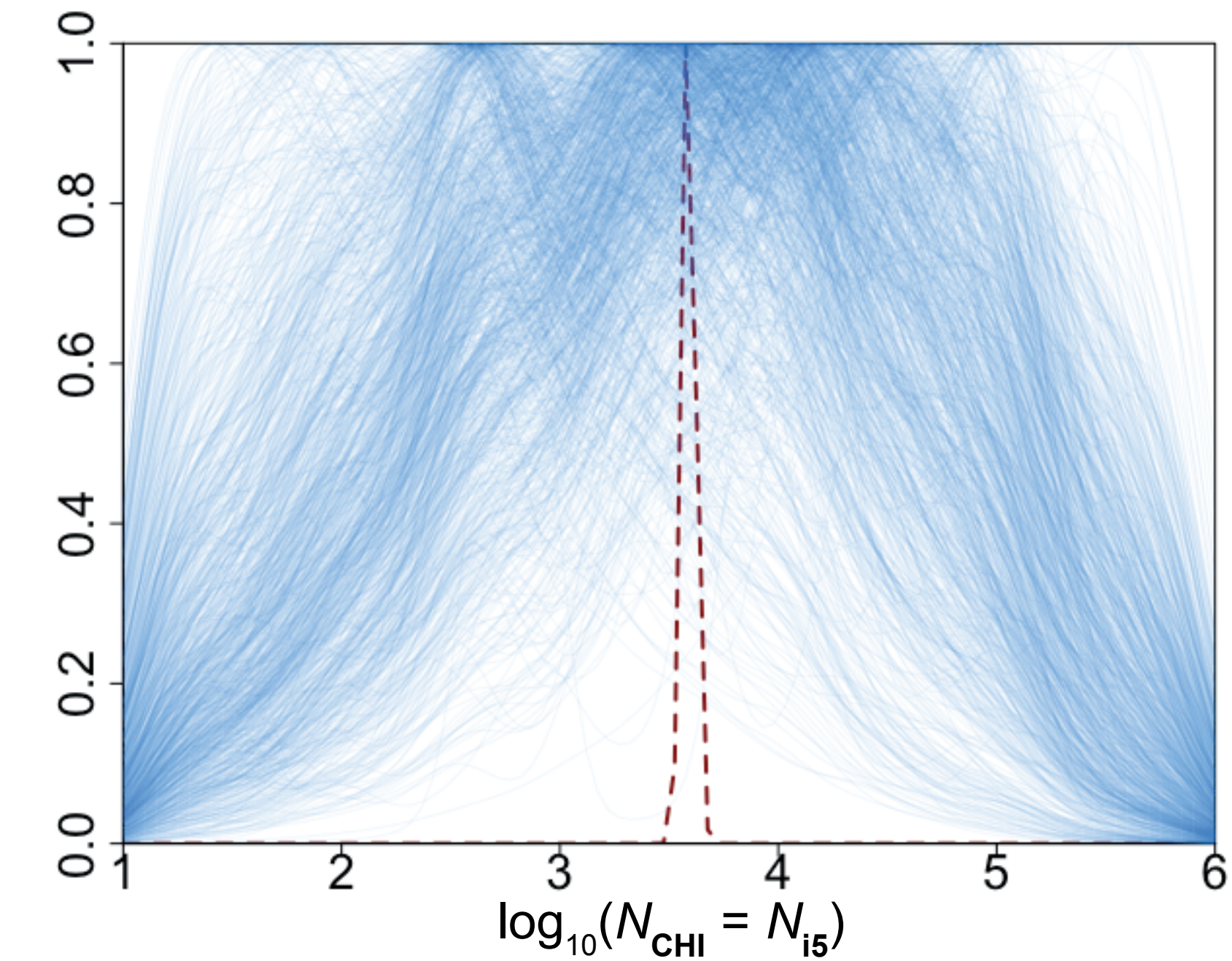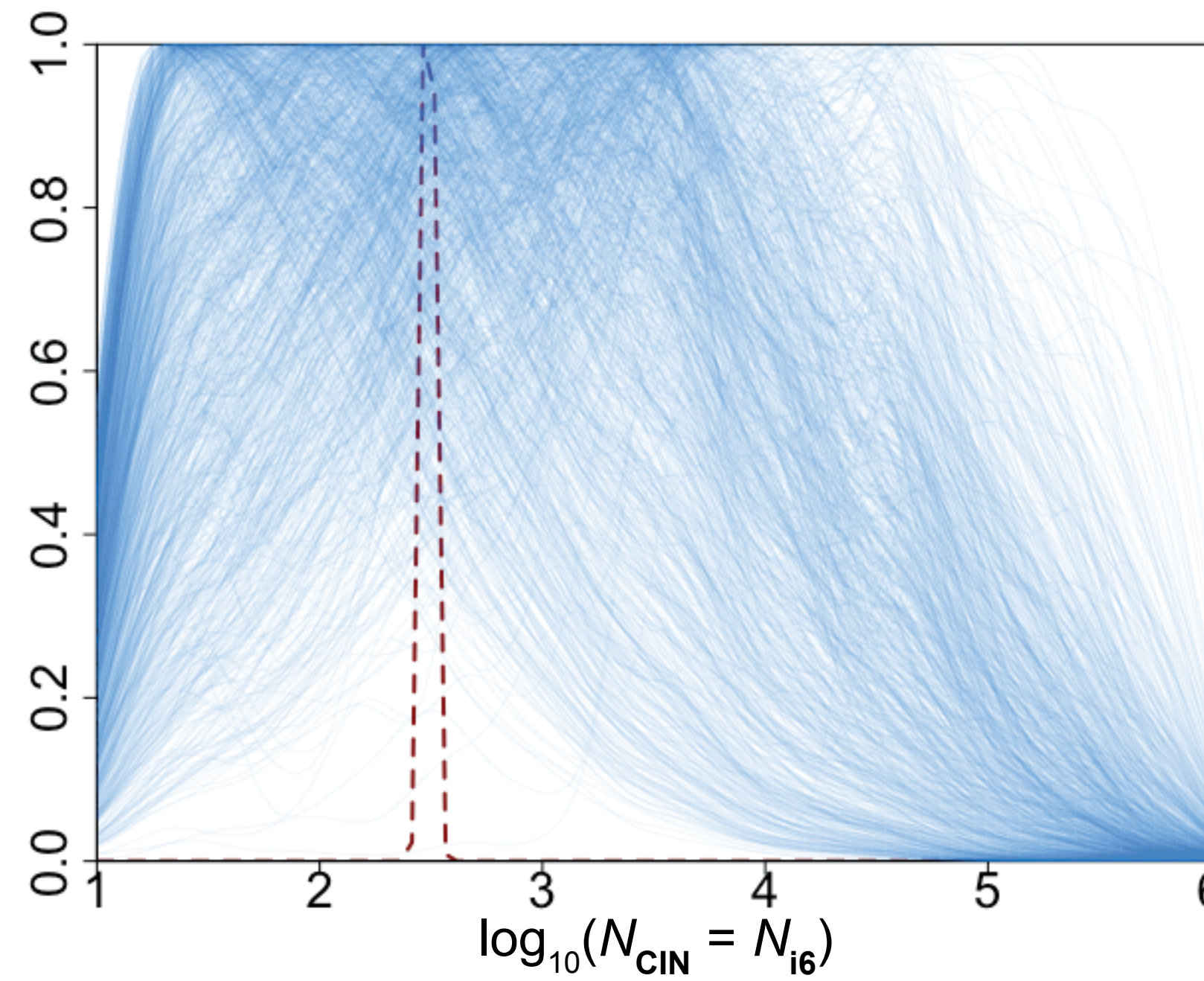

Supplement: Supplementary file 2 — Fig S1‐S16 [file MEN-21-2719-s002.zip › Supplementary Figures/Figure_S14.pdf]

A)

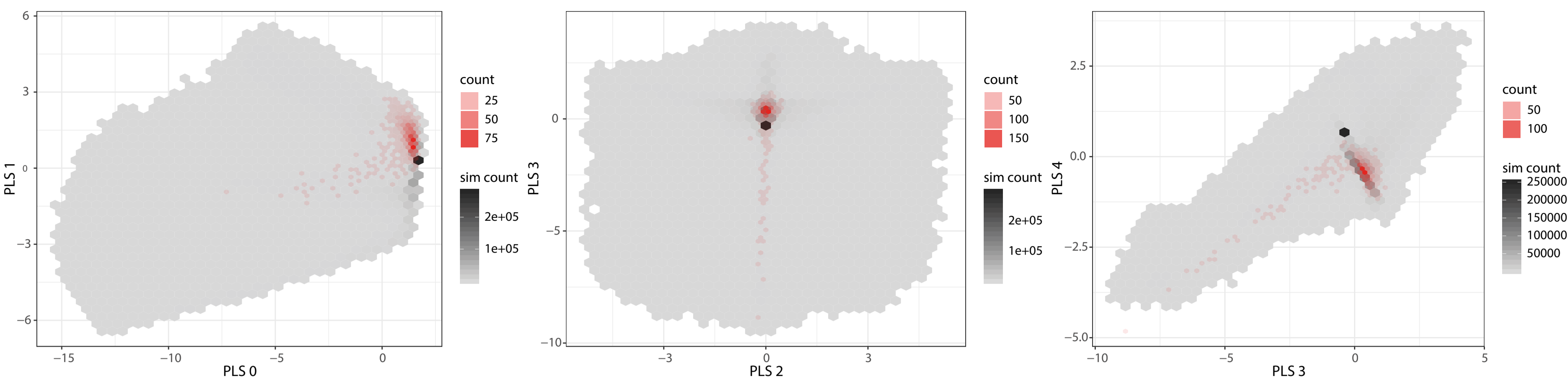

B)

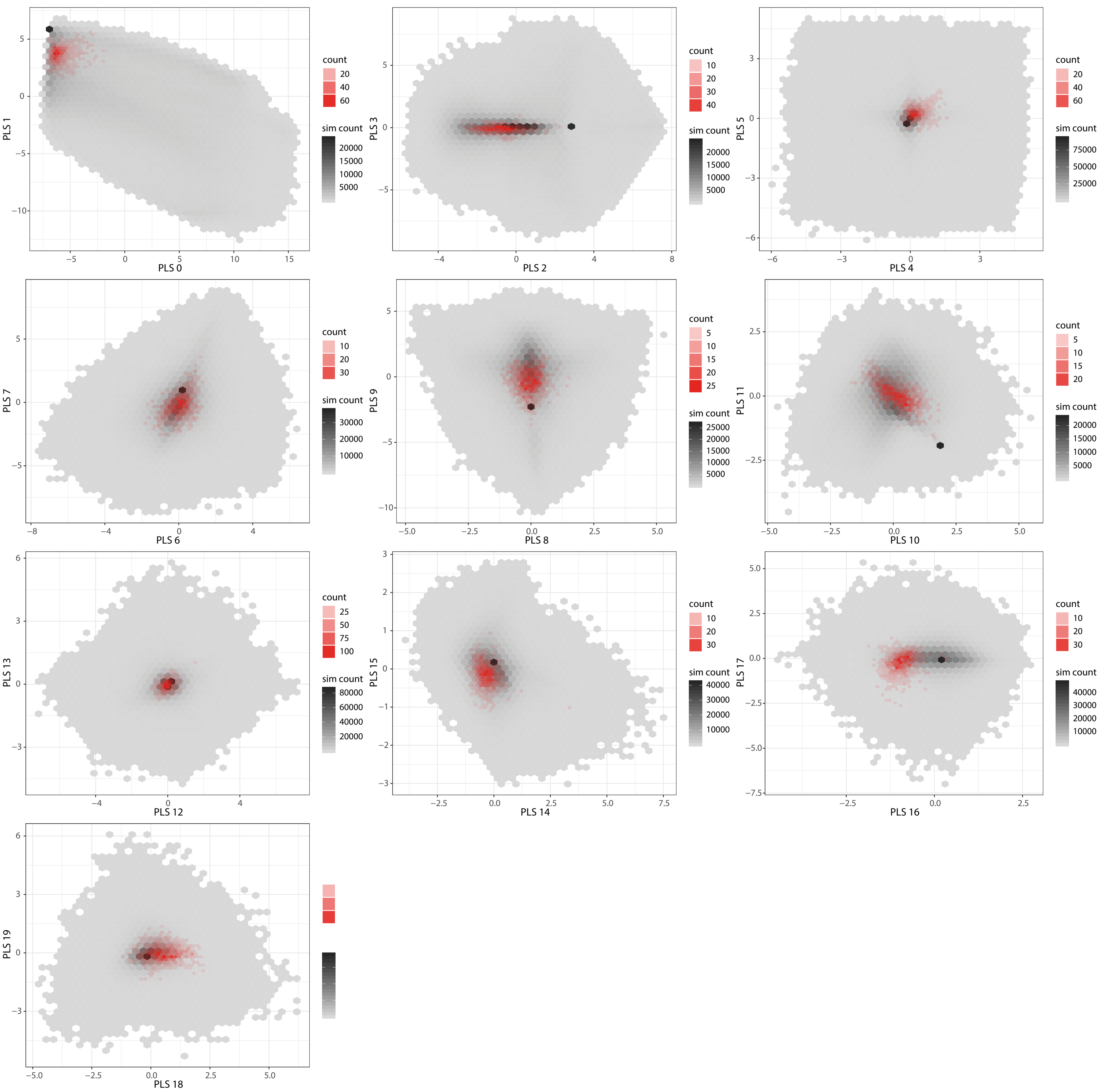

Supplement: Supplementary file 2 — Fig S1‐S16 [file MEN-21-2719-s002.zip › Supplementary Figures/Figure_S15.pdf]

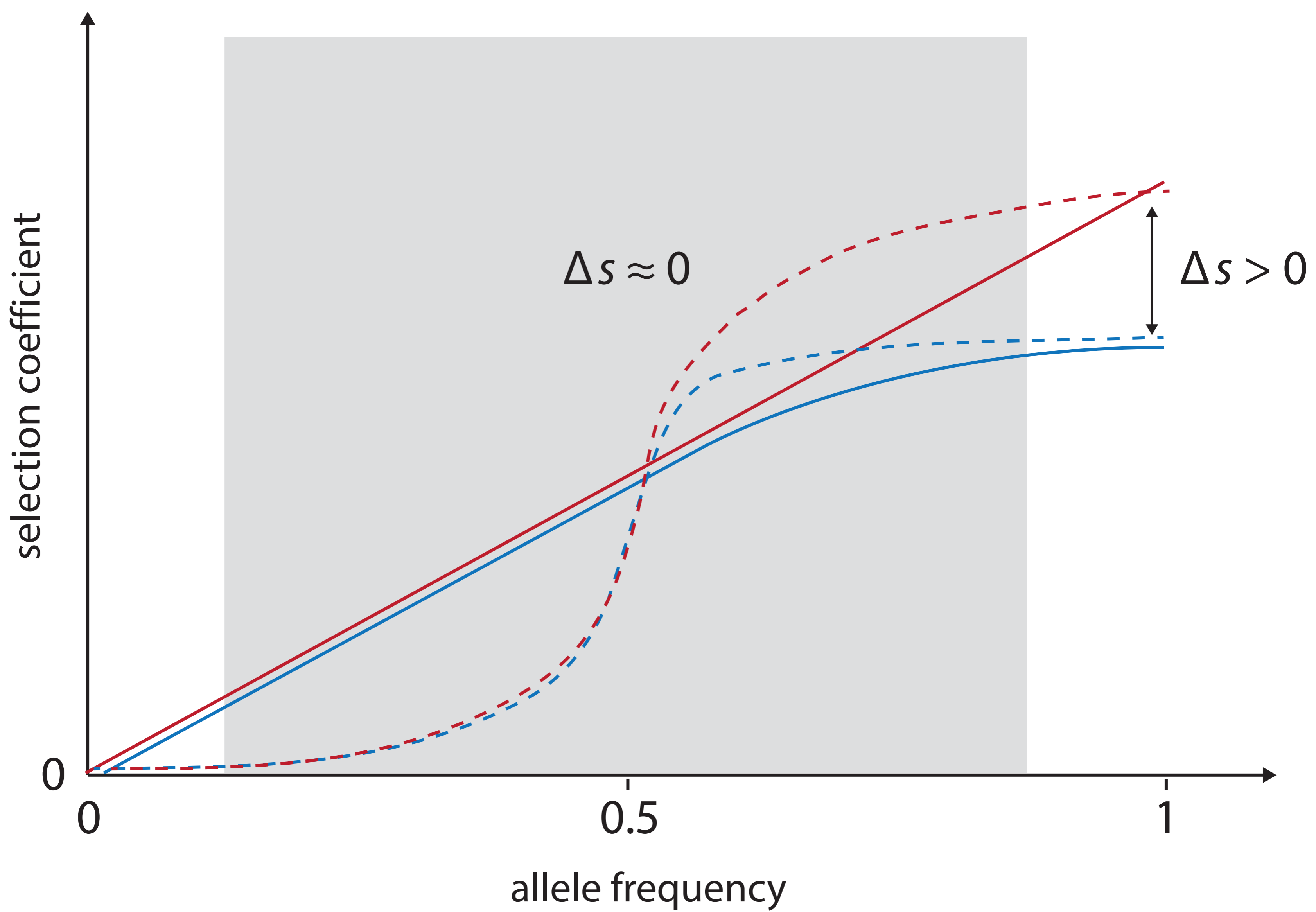

Supplement: Supplementary file 2 — Fig S1‐S16 [file MEN-21-2719-s002.zip › Supplementary Figures/Figure_S16.pdf]

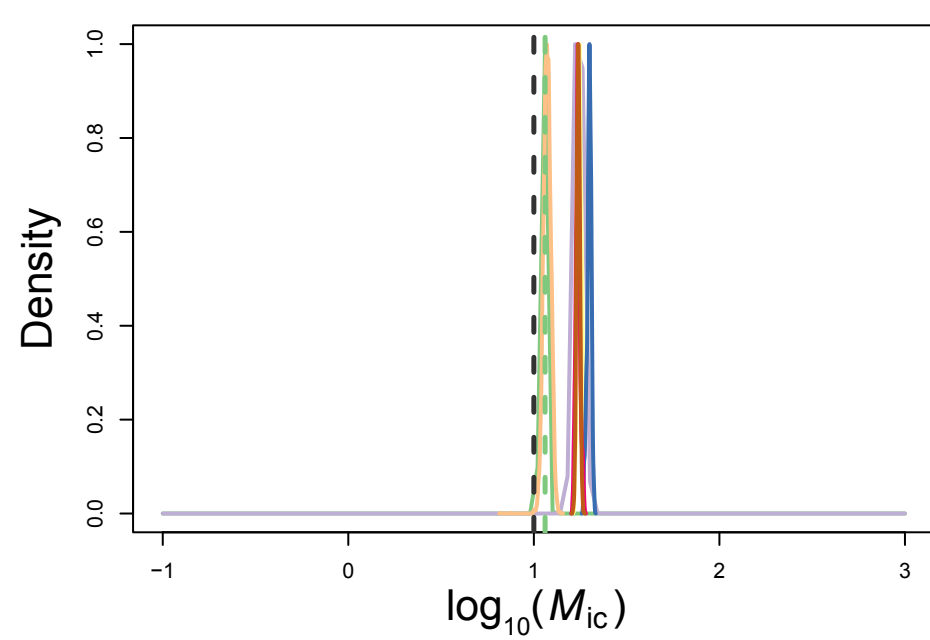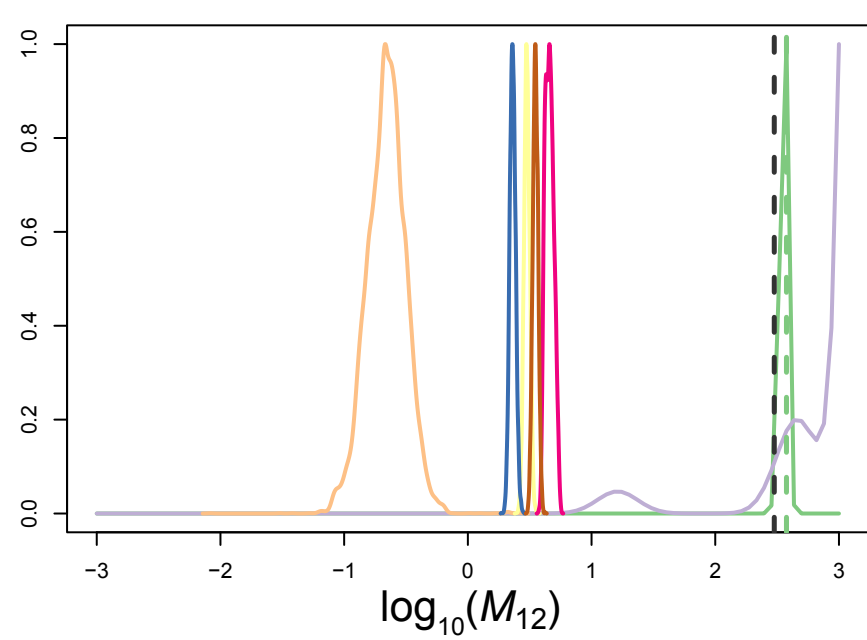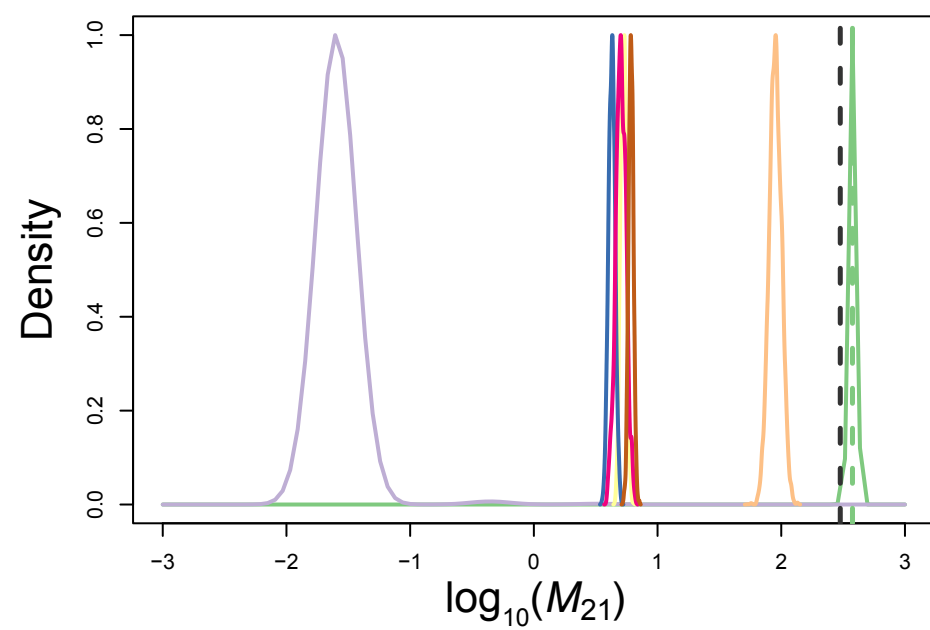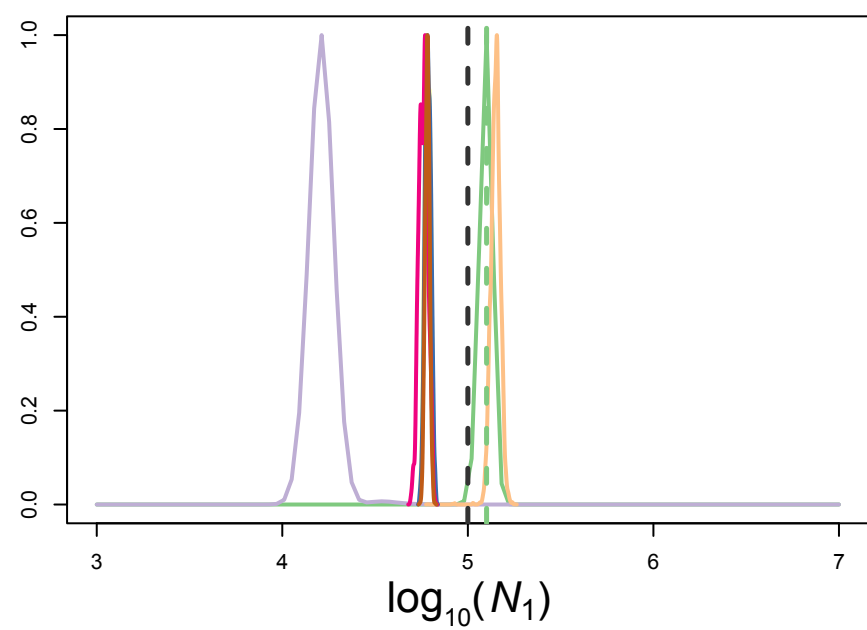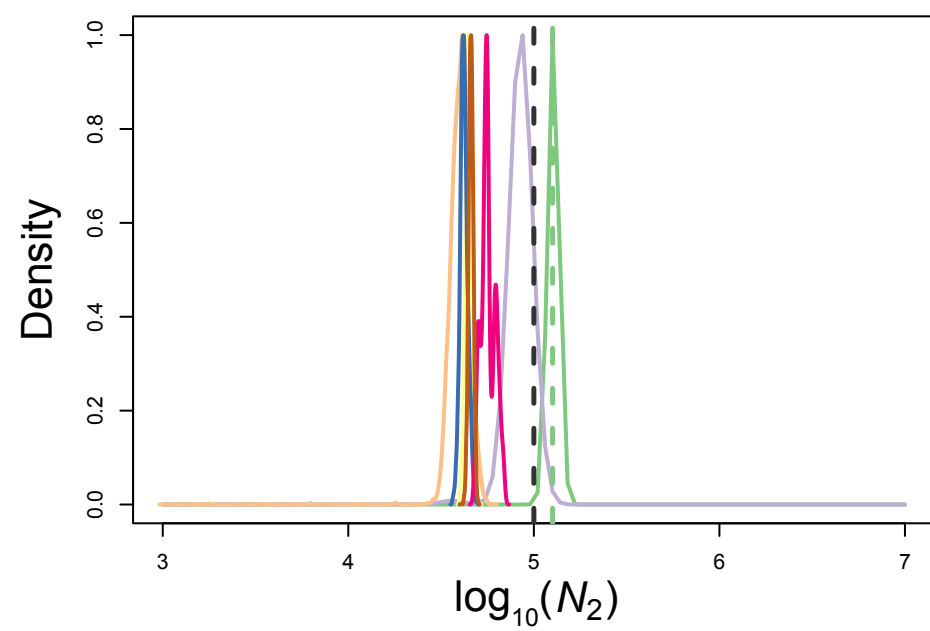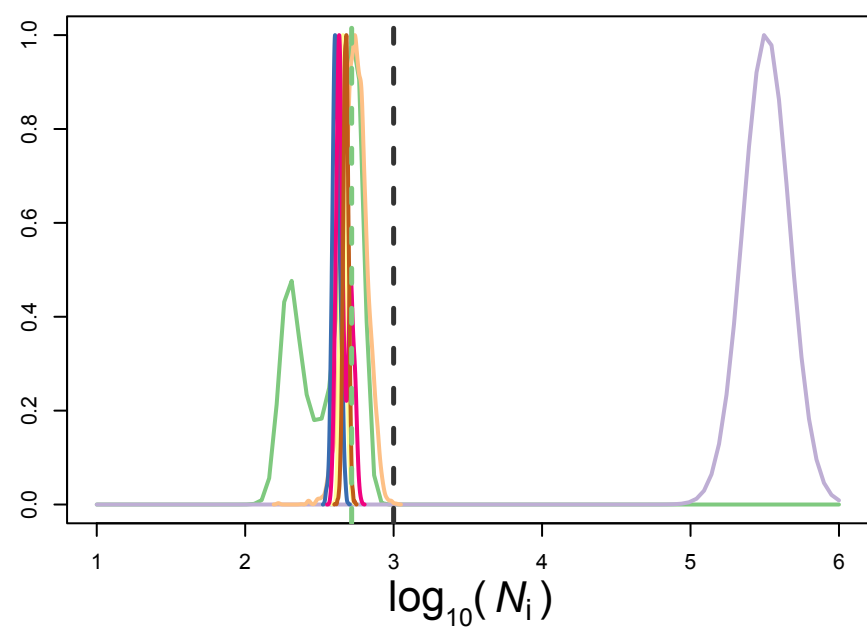

Supplement: Supplementary file 2 — Fig S1‐S16 [file MEN-21-2719-s002.zip › Supplementary Figures/Figure_S2.pdf]

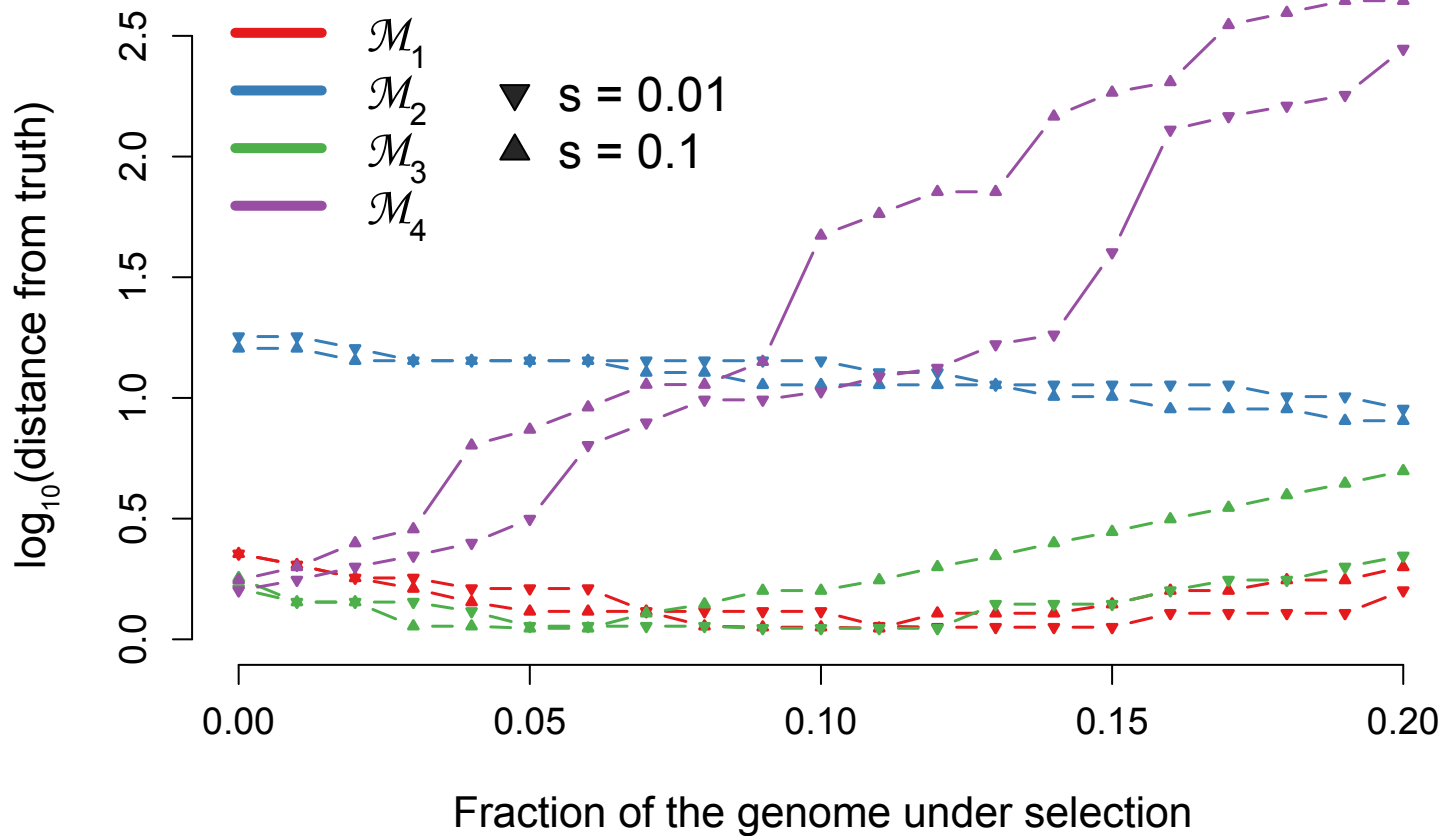

Supplement: Supplementary file 2 — Fig S1‐S16 [file MEN-21-2719-s002.zip › Supplementary Figures/Figure_S3.pdf]

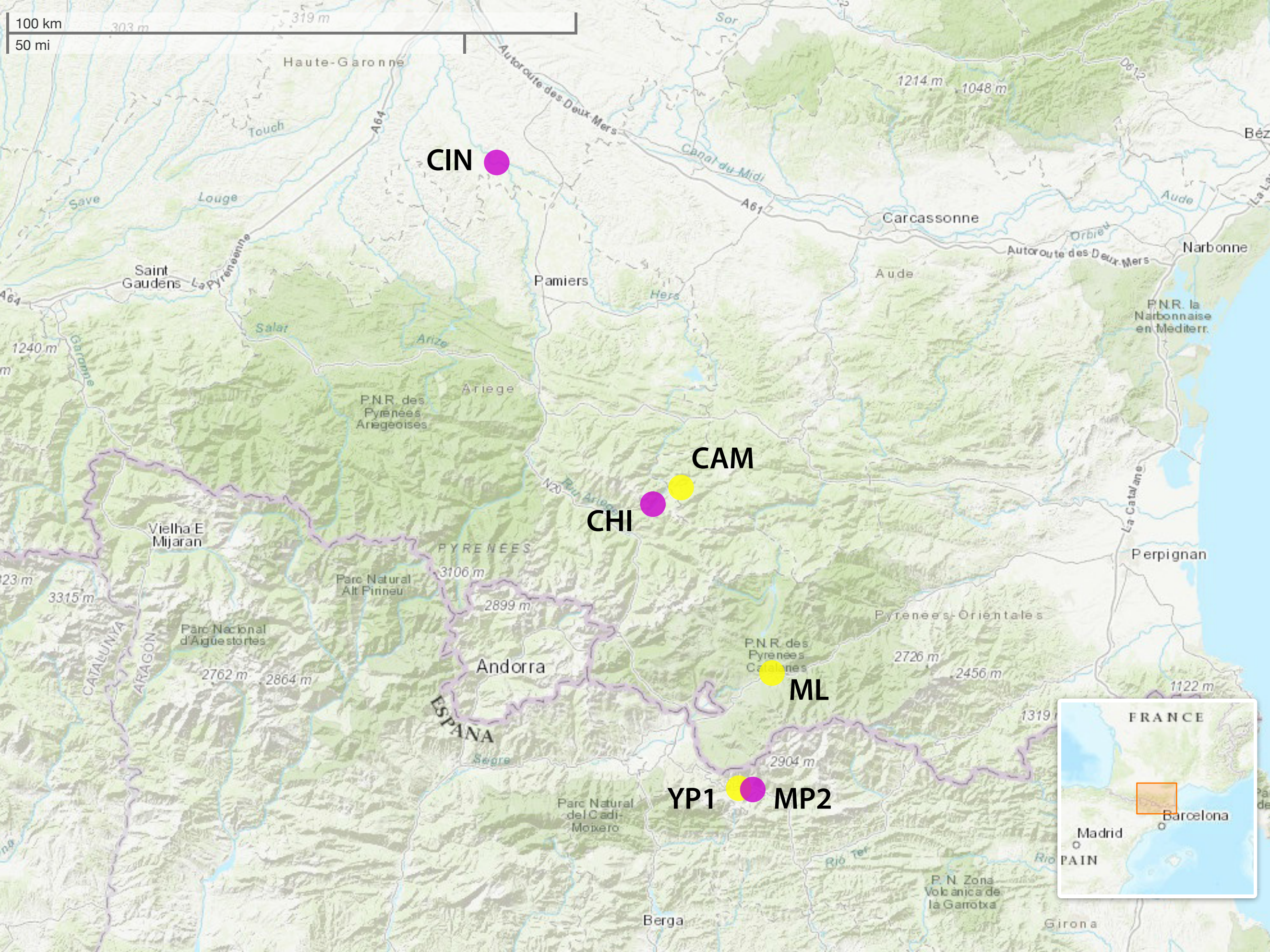

Supplement: Supplementary file 2 — Fig S1‐S16 [file MEN-21-2719-s002.zip › Supplementary Figures/Figure_S4.pdf]

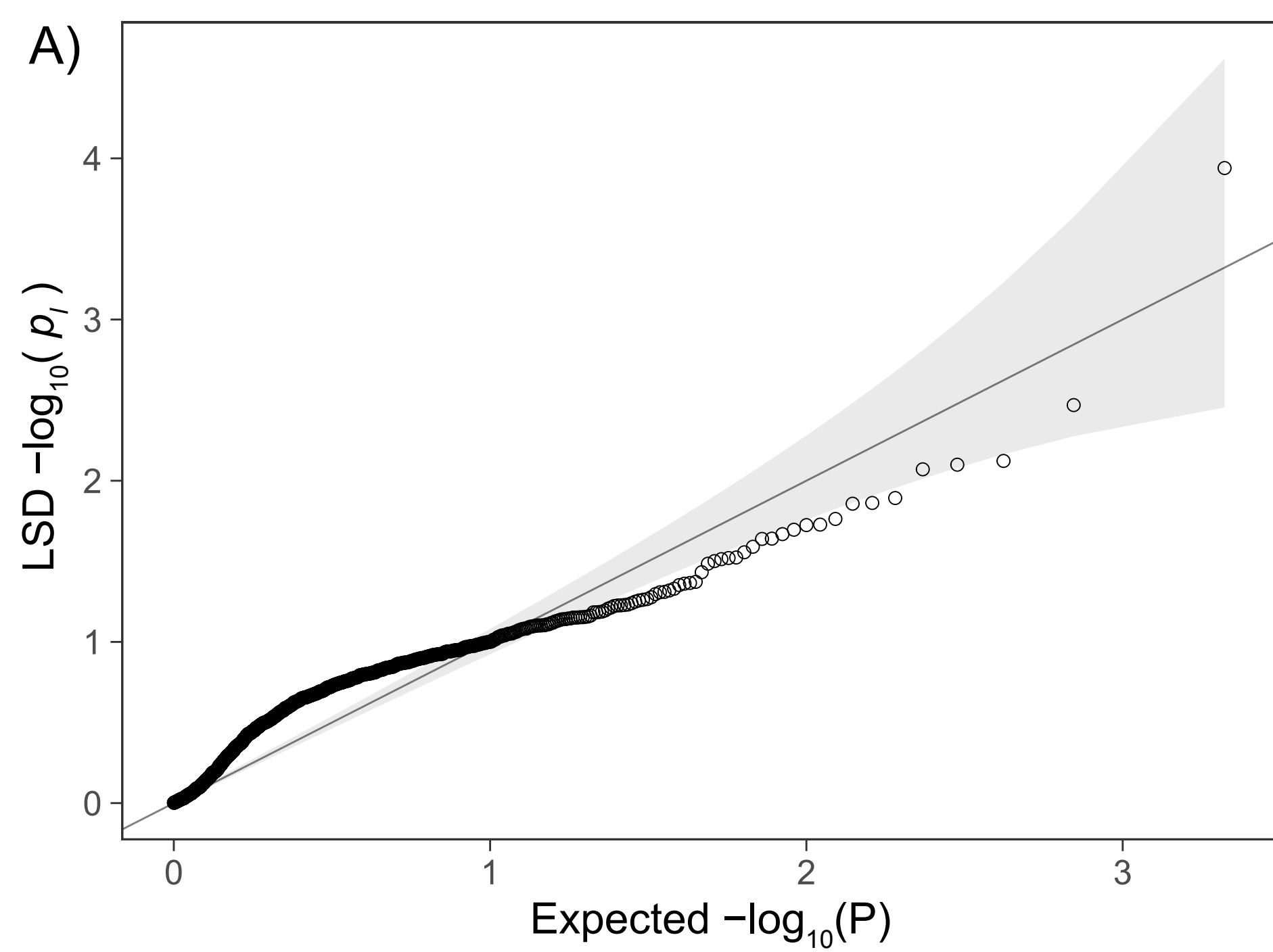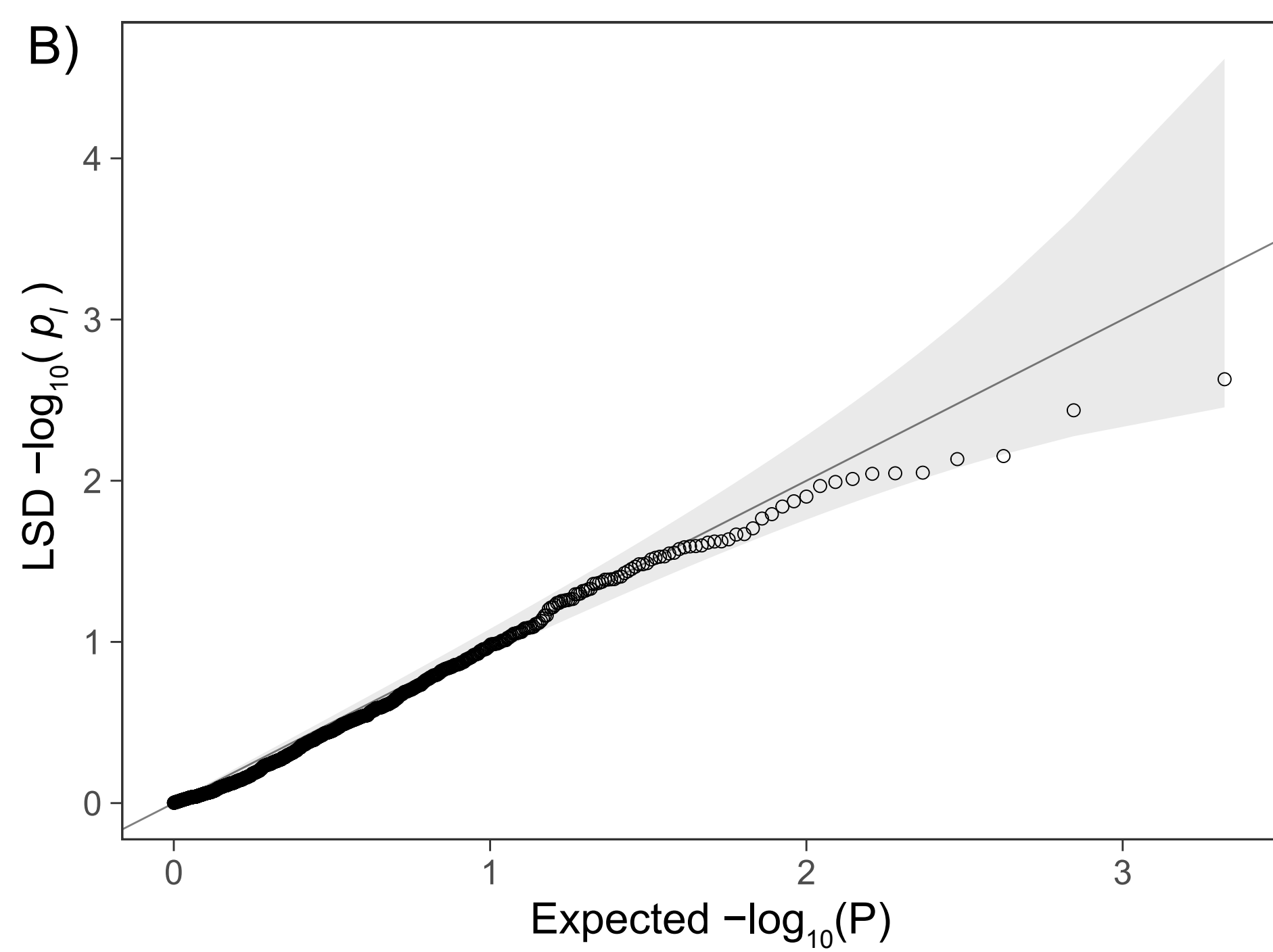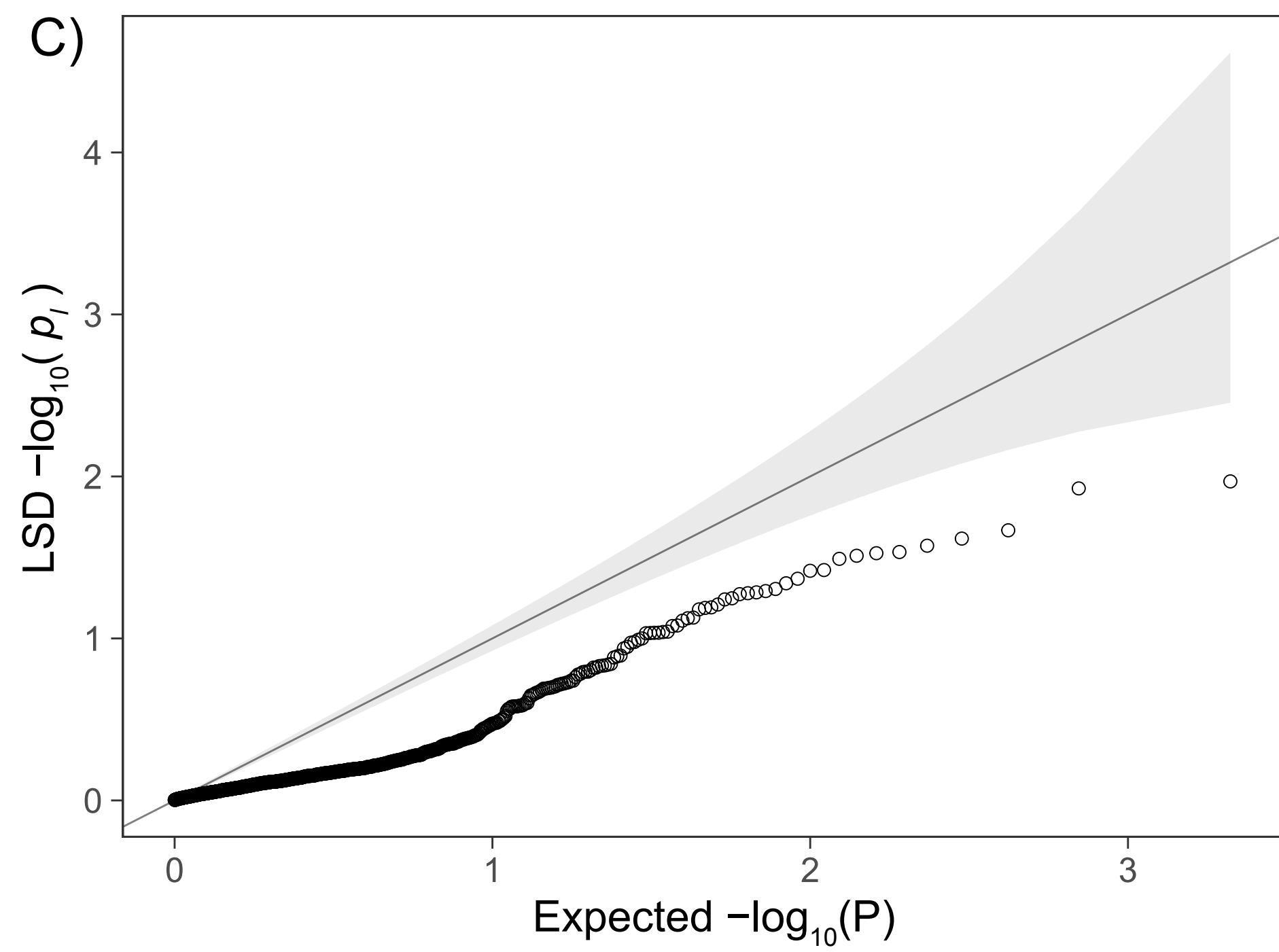

Supplement: Supplementary file 2 — Fig S1‐S16 [file MEN-21-2719-s002.zip › Supplementary Figures/Figure_S5.pdf]

**A)**

Onset of selection

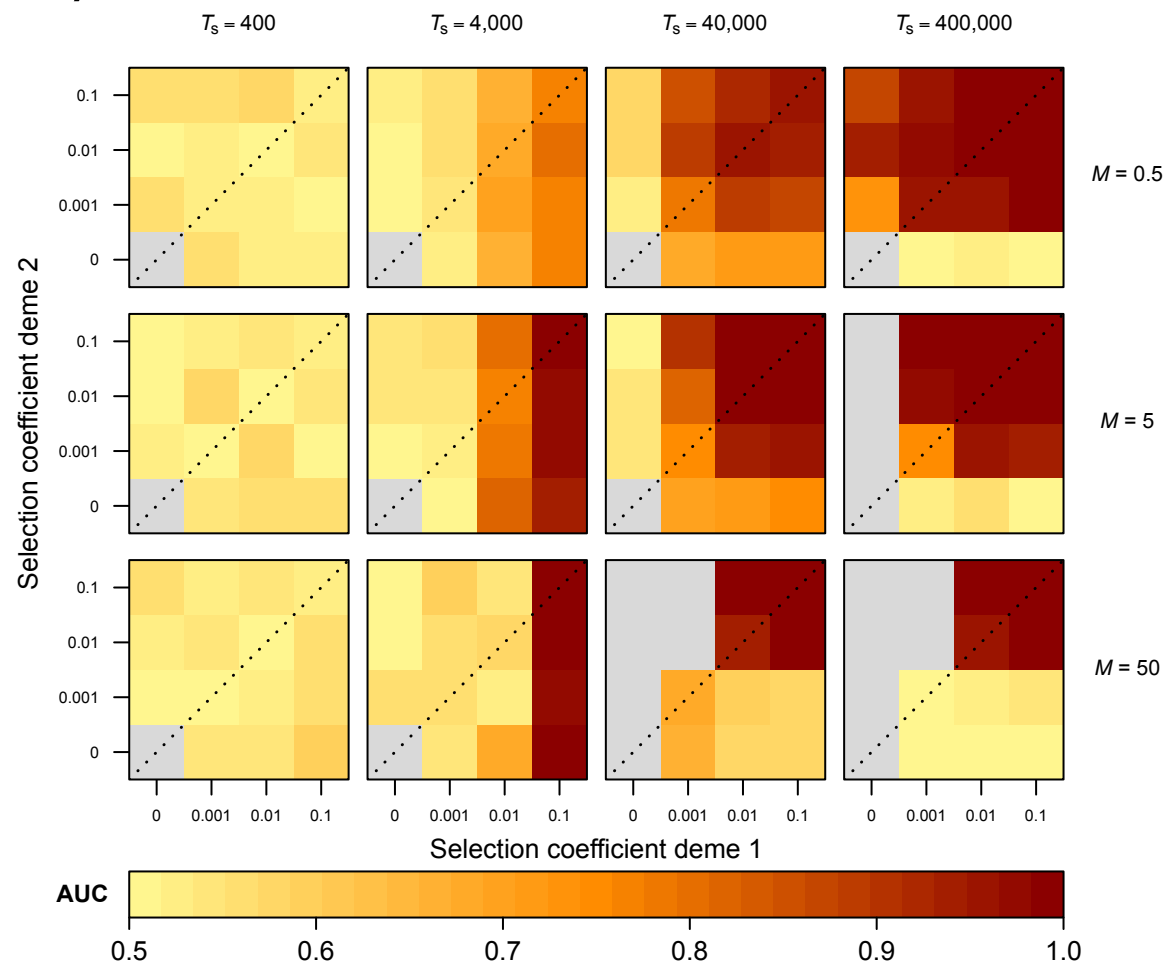**B)**

Onset of selection

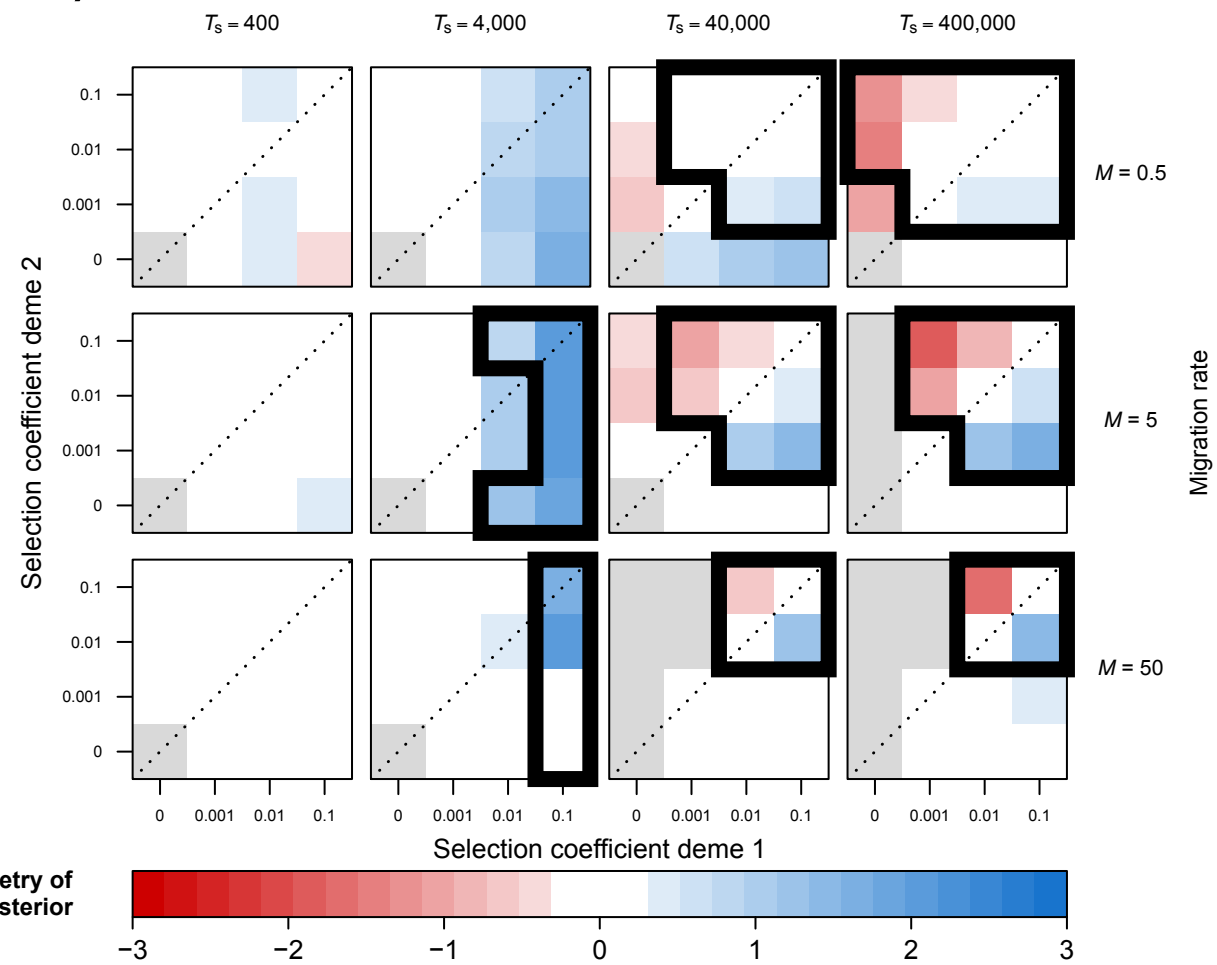

Supplement: Supplementary file 2 — Fig S1‐S16 [file MEN-21-2719-s002.zip › Supplementary Figures/Figure_S6.pdf]

A)

Onset of selection

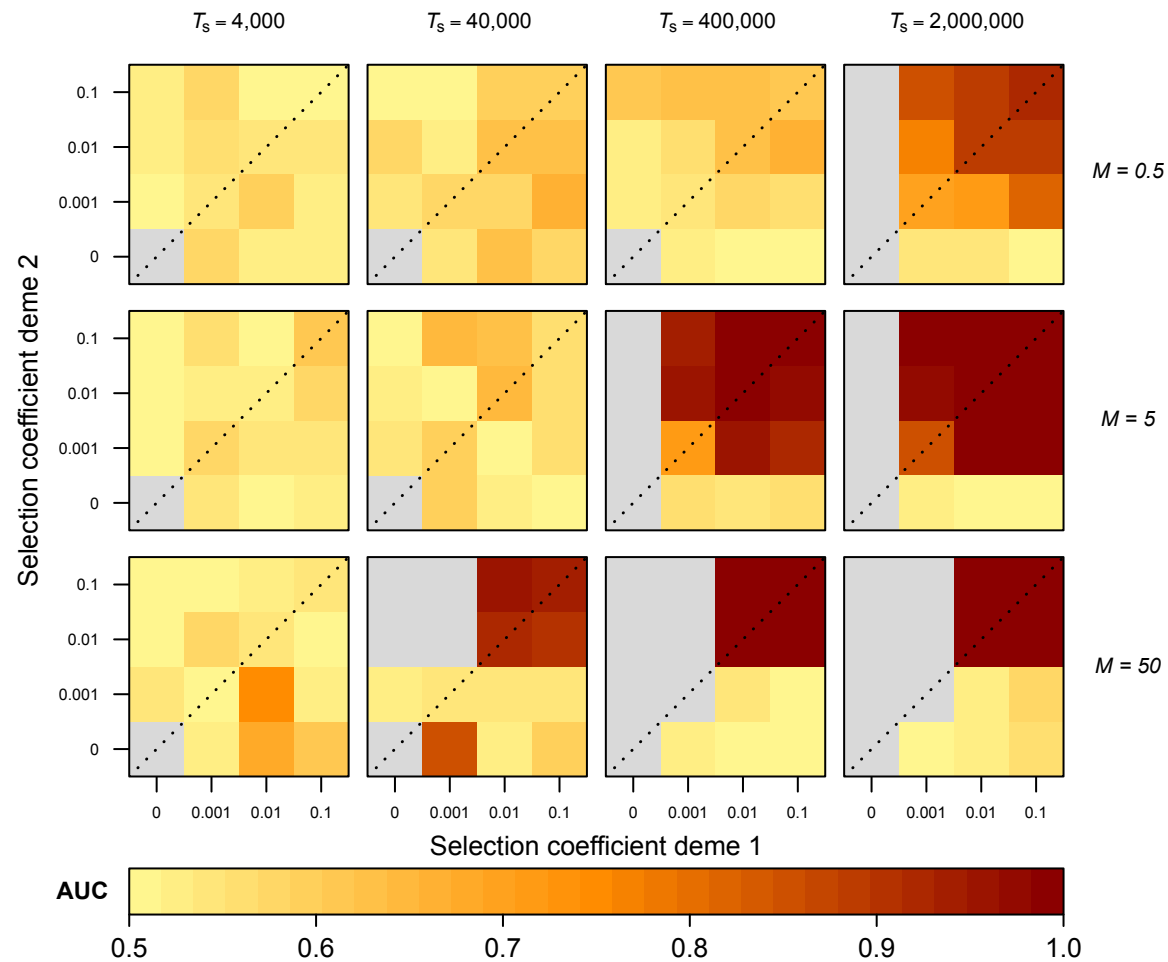

B)

Onset of selection

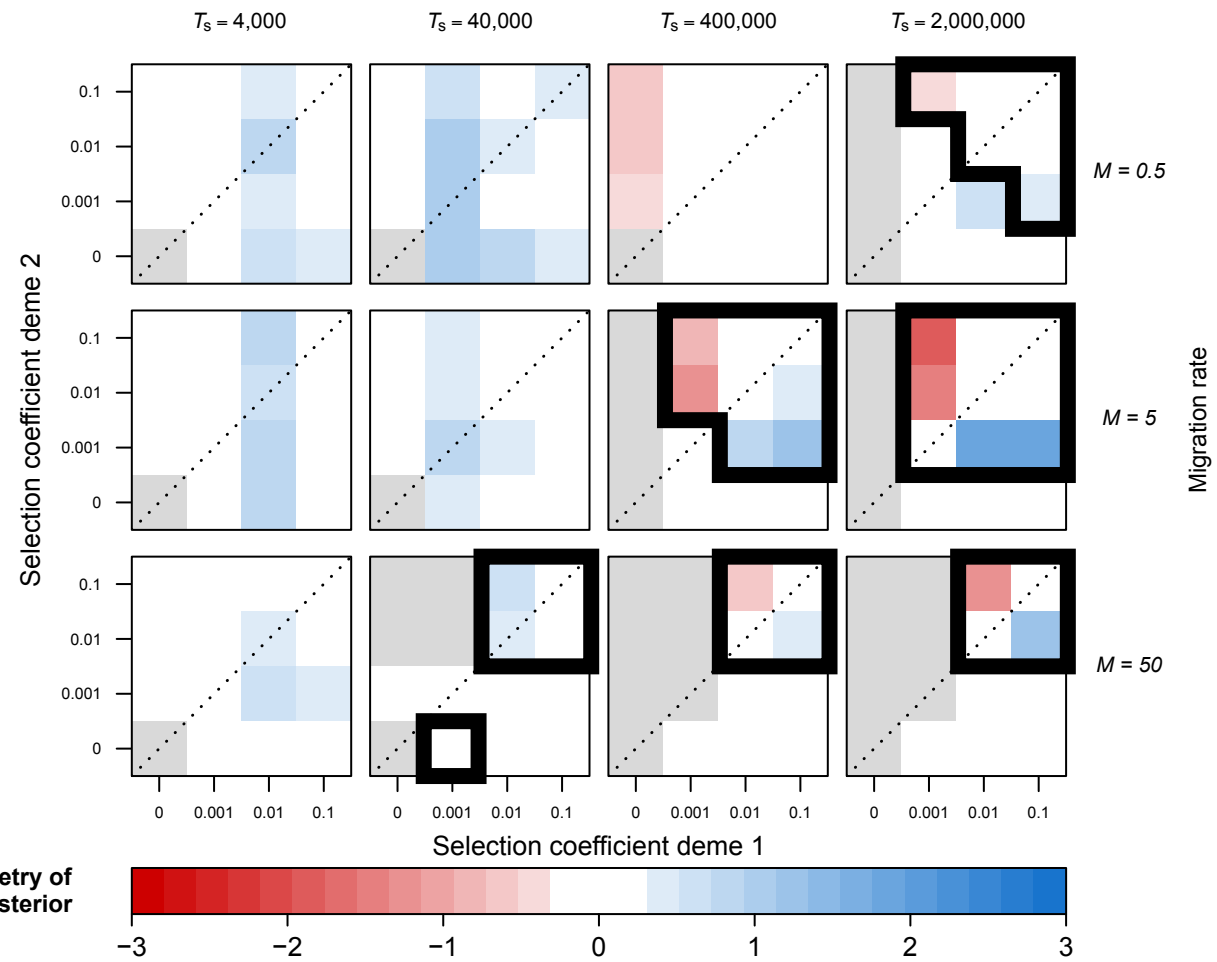

Supplement: Supplementary file 2 — Fig S1‐S16 [file MEN-21-2719-s002.zip › Supplementary Figures/Figure_S7.pdf]

A)

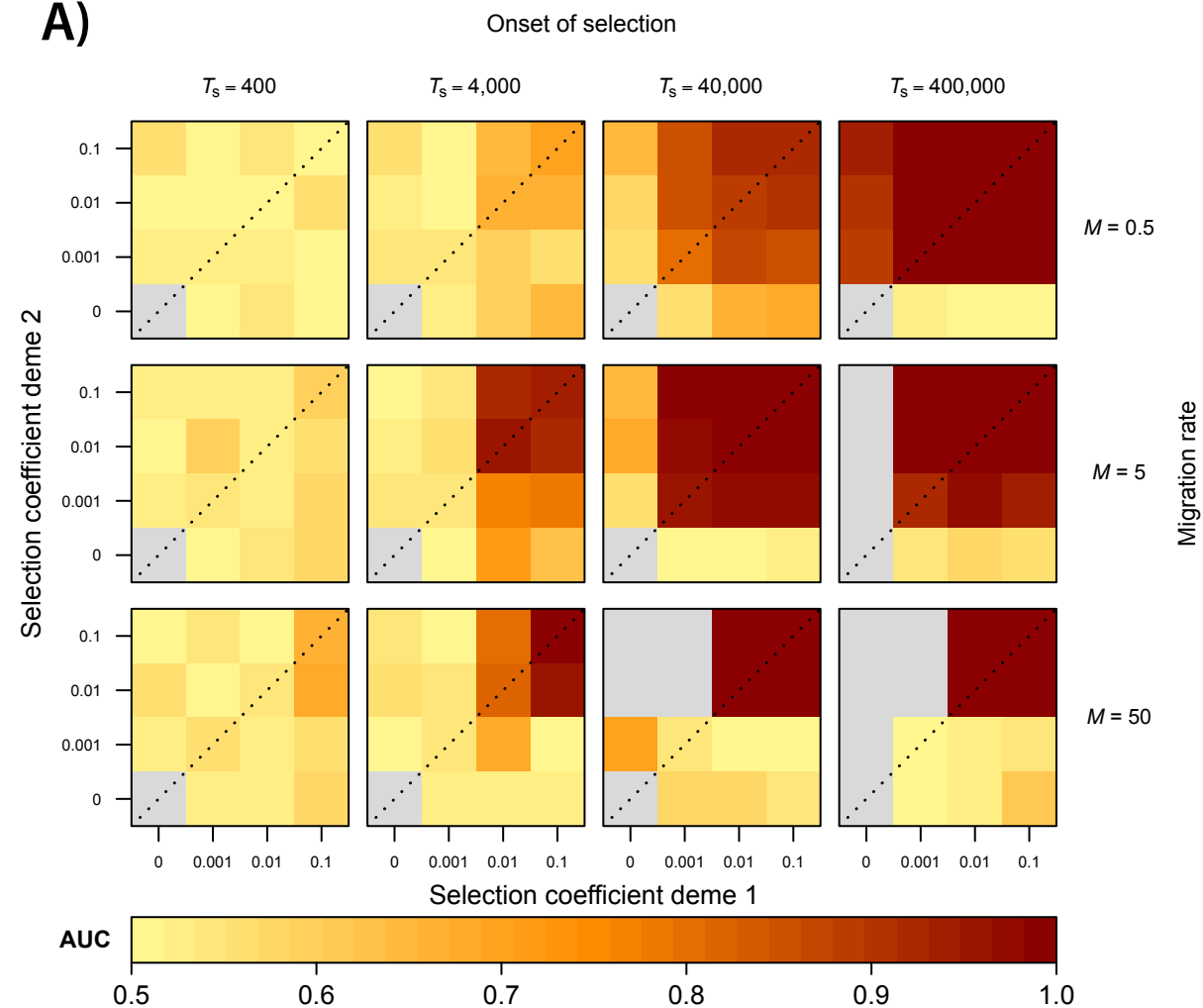

B)

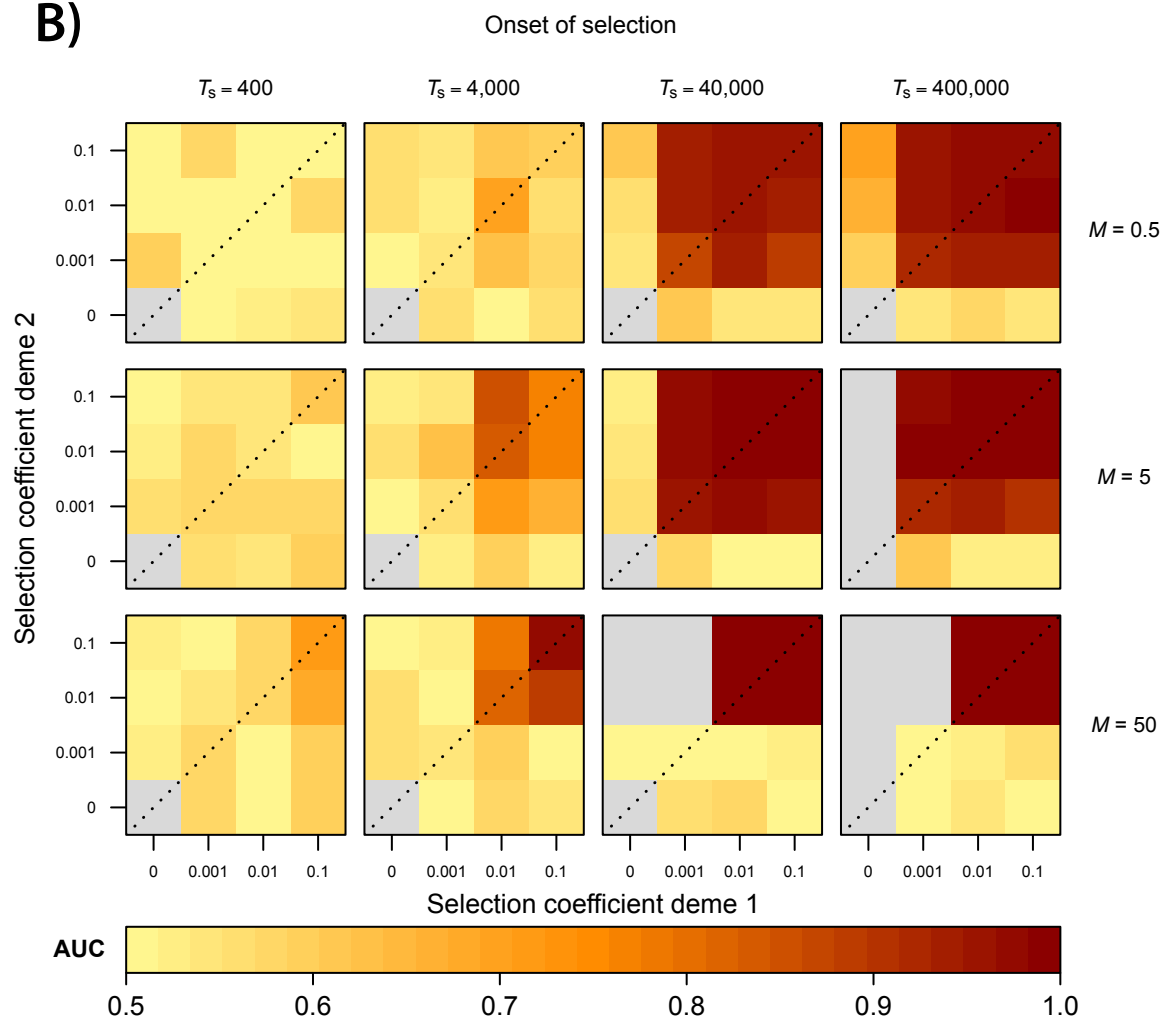

C)

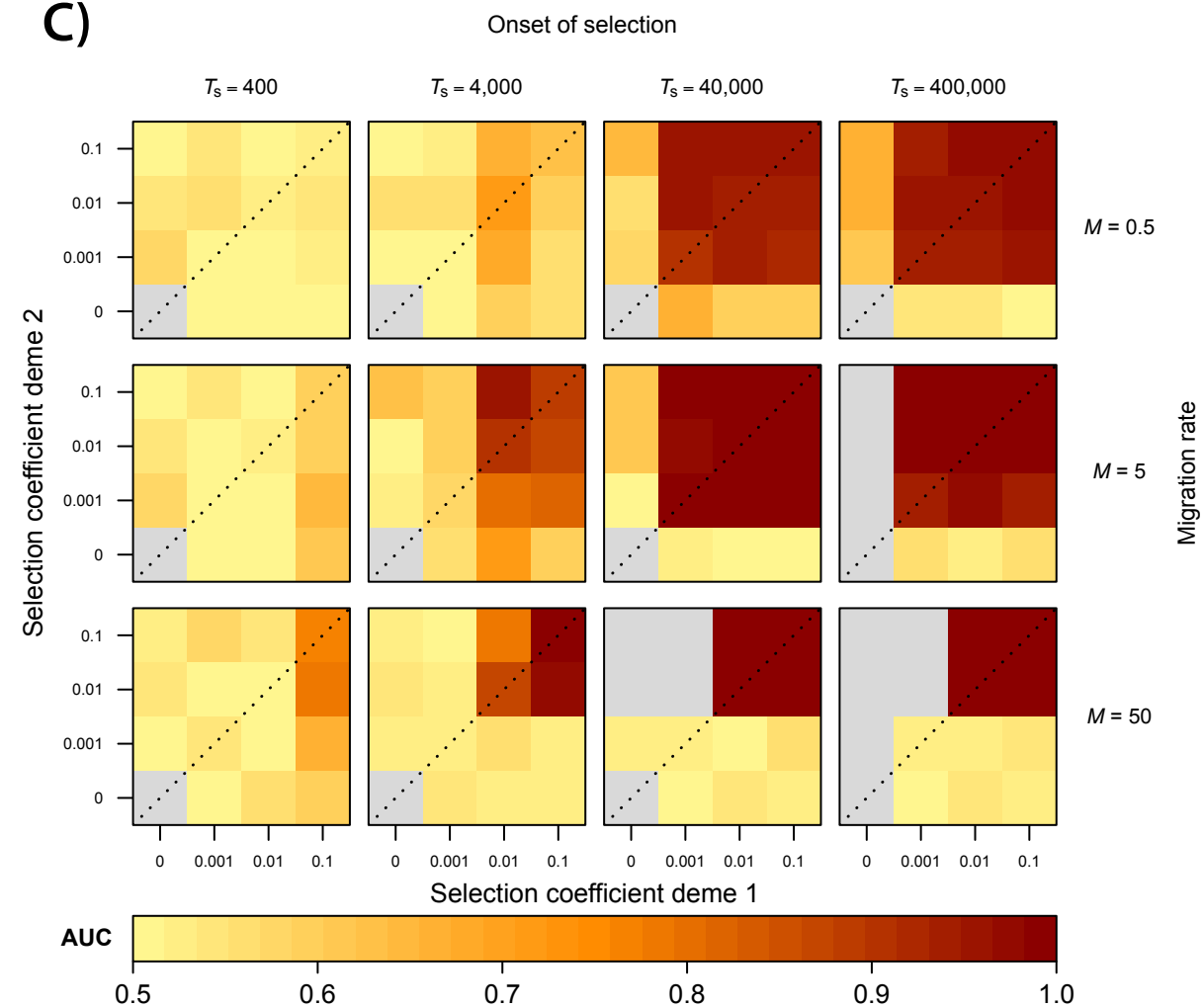

Supplement: Supplementary file 2 — Fig S1‐S16 [file MEN-21-2719-s002.zip › Supplementary Figures/Figure_S9.pdf]
